# A cross-species atlas platform for comparative neuroscience in teleost fish

**DOI:** 10.1038/s41467-026-77879-1

**Published:** 2026-09-25

**Authors:** Sumit Kumar Vohra, Tinatini Tavhelidse-Suck, Simon Knoblich, Ali Seleit, Jonathan Boulanger-Weill, Sydney Chambule, Ariel Aspiras, Cristina Santoriello, Owen Randlett, Joachim Wittbrodt, Jeff W. Lichtman, Mark Fishman, Hans-Christian Hege, Daniel Baum, Florian Engert, Yasuko Isoe

**Affiliations:** 1 Visual and Data-Centric Computing, Zuse Institute Berlin, Takustraße 7, Berlin, Germany; 2 Research group behavioral neuroscience, Leibniz-Institut für Neurobiologie (LIN), Brenneckestraße 6, Magdeburg, Germany; 3 Centre for Organismal Studies (COS) Heidelberg, Heidelberg University, Im Neuenheimer Feld 230, Heidelberg, Germany; 4 Developmental Biology Unit, European Molecular Biology Laboratory (EMBL) Heidelberg, Meyerhofstraße 1, Heidelberg, Germany; 5 Department of Molecular and Cellular Biology, Faculty of Arts and Sciences, Harvard University, Cambridge, Massachusetts USA; 6 Sorbonne Université, INSERM, CNRS, Institut de la Vision, Paris, France; 7 Department of Stem Cell and Regenerative Biology, Faculty of Arts and Sciences, Harvard University, Cambridge, Massachusetts USA; 8 Laboratoire MeLiS, Université Claude Bernard Lyon 1 - CNRS UMR5284 - Inserm U1314, Institut NeuroMyoGène, Faculté de Médecine et de Pharmacie, 8 Avenue Rockefeller, Lyon, France

**Keywords:** Fluorescence imaging, Classification and taxonomy, Data integration, Molecular neuroscience

## Abstract

Uncovering general principles connecting neural circuits to behaviour requires comparative brain analyses across species. To advance these efforts, we developed a standardised workflow and a unified brain atlas platform for the quantitative comparison of neural structures between two teleost larvae, medaka (*Oryzias latipes*) and zebrafish (*Danio rerio*). Crucially, the platform seamlessly integrates user-generated image volumes, facilitating quantitative comparisons of anatomical, transcriptional, and functional data across species and laboratories. Utilising these integrated tools, we identified regional anatomical differences and differentially expressed genes between the species. We further demonstrated functionally distinct telencephalic regions in both species that exhibit divergent responses to social stimuli, suggesting an evolutionary divergence in social processing mechanisms. This web-based, interactive atlas platform promotes data exchange and knowledge sharing within the teleost community. Its scalable design permits effortless multi-group contributions, enabling expansion into a continuously growing, globally accessible database.

## Abstract (web summary)

This presents a scalable interactive brain atlas, which enables quantitative comparison of anatomical, molecular and functional data across zebrafish and medaka larvae, revealing both conserved and divergent features of brain organization and social processing.

## Introduction

While the basic blueprint of the brain is well-conserved across vertebrates, species exhibit diverse brain nuclei even within the same animal class. Utilising different animal models within the same class provides a powerful and efficient means to understand how neural circuits give rise to behaviour, revealing both generally conserved and species-specific mechanisms of neural function. For example, in rodent models, mountain and prairie voles can be easily distinguished with respect to different mating habits, with oxytocinergic neurons playing a critical role in this process^1^. Primates, macaques, marmosets and, indeed, humans all display wildly different levels of performance in a whole range of behavioural tasks^2,3^.

For the analysis of evolutionary neurobiological adaptations, it is useful to have neuroanatomical datasets in hand that allow for the quantitative comparisons of brain regions across species. Our efforts here are aimed at assisting with this challenge. To that end, we parcelled the whole brain into mutually exclusive and comprehensively exhaustive (MECE) regions based on morphological and transcriptional features in both species, thereby providing a spatial map that serves as the basis for subsequent analyses. We believe that this parcellation is the first and critical step for a quantitative and direct cross-species comparison.

Teleost larvae serve as valuable model organisms for elucidating behaviour and the underlying neural circuits in the context of comparative studies for several reasons: (1) their small body size facilitates brain-wide imaging using different modalities^4,5^, (2) their transparency allows for in vivo analysis of brain activity throughout the whole brain at the cellular level^6^, (3) as vertebrates, they allow for extrapolation of general insights to mammals such as mice and humans^7^. Furthermore, teleost provide an excellent opportunity to study brain evolution, since a large number of species (more than 30,000) adapted to very different ecological niches. This allows us to study closely related species that are adapted to different environments, display slightly different behaviours, or have brain modifications^8^.

Zebrafish (*Danio rerio*) larvae have been widely used in the context of system neuroscience; however, this approach has not yet been fully extended to other teleost species^9^, an endeavour necessary for studying behaviours and neural circuits in an explicitly comparative fashion.

Here, we selected medaka fish (*Oryzias latipes*) as another fish species because of its phylogenetic distance from zebrafish – having diverged 250 million years ago – and its adaptation to ecologically different natural environments^10,11^. Notably, medaka exhibit significantly higher tolerance to salinity^12^ and inhabit clearer waters with relatively fast-moving flow^13^, while zebrafish naturally inhabit slow-moving and murky waters^14,15^, yet both species share a wide range of comparable anatomical and behavioural phenotypes.

Current brain atlases for adult^16,17^ and larval medaka fish^18^ lack the resolution and standardisation required for quantitative and systematic studies across teleost fish. To address this issue, we have extended the existing zebrafish brain atlas, Z-brain^19,20^, to seamlessly incorporate larval medaka brain (M-brain). Our cross-species platform, called FishExplorer (https://fishexplorer.zib.de/), enables quantitative and comprehensive comparisons of anatomical brain regions across these fish species. Specifically, we have parcelled each brain into corresponding *mutually exclusive and comprehensively exhaustive* (MECE) regions that encompass the entire brain without overlaps and gaps. This approach helps to quantitatively compare the whole brain across species in terms of volume and gene expression. In addition, we support the open-ended generation of miscellaneous (MISC) masks that complement MECE regions. These masks can serve as repositories and descriptors for any arbitrary subregions that are of specific interest, such as neuronal subpopulations that express specific neurotransmitters, or functionally or transcriptionally distinct subregions (that may span multiple MECE areas), or neuronal populations that are defined by their ontogeny.

In this study, we present FishExplorer (https://fishexplorer.zib.de/), a newly improved and extended atlas platform for visualising and analysing teleost anatomy with the purpose of cross-species comparison. FishExplorer offers the following main features: (1) Web-based platform: The platform is ready to use in the browser without software installation. (2) Visualisation and analysis tool: FishExplorer provides tools for interactive visualisation and quantitative analysis of region masks as well as a Fiji plug-in (NeMO), which greatly facilitates segmentation of brain regions and creation of new masks. (3) Scalability: The platform’s database can be expanded in many ways. In its present form, it allows for the inclusion of new tissue types; future versions will also allow the addition of different species and later developmental stages. (4) Extensive image and knowledge database: FishExplorer provides an extensive base of annotated image data for different species and user knowledge stored in annotations and masks. (5) Community platform: Users can upload and analyse their data utilising all publicly available data in the platform. The users have exclusive access to their private repository, ensuring data security before publication, and they can seamlessly share their data with the community after publication. We believe that FishExplorer can become a central point of access for teleost research data, fostering collaborations between researchers and accelerating scientific discovery.

## Results

### Region descriptors in FishExplorer: MECE and MISC masks

**Fig. 1:**
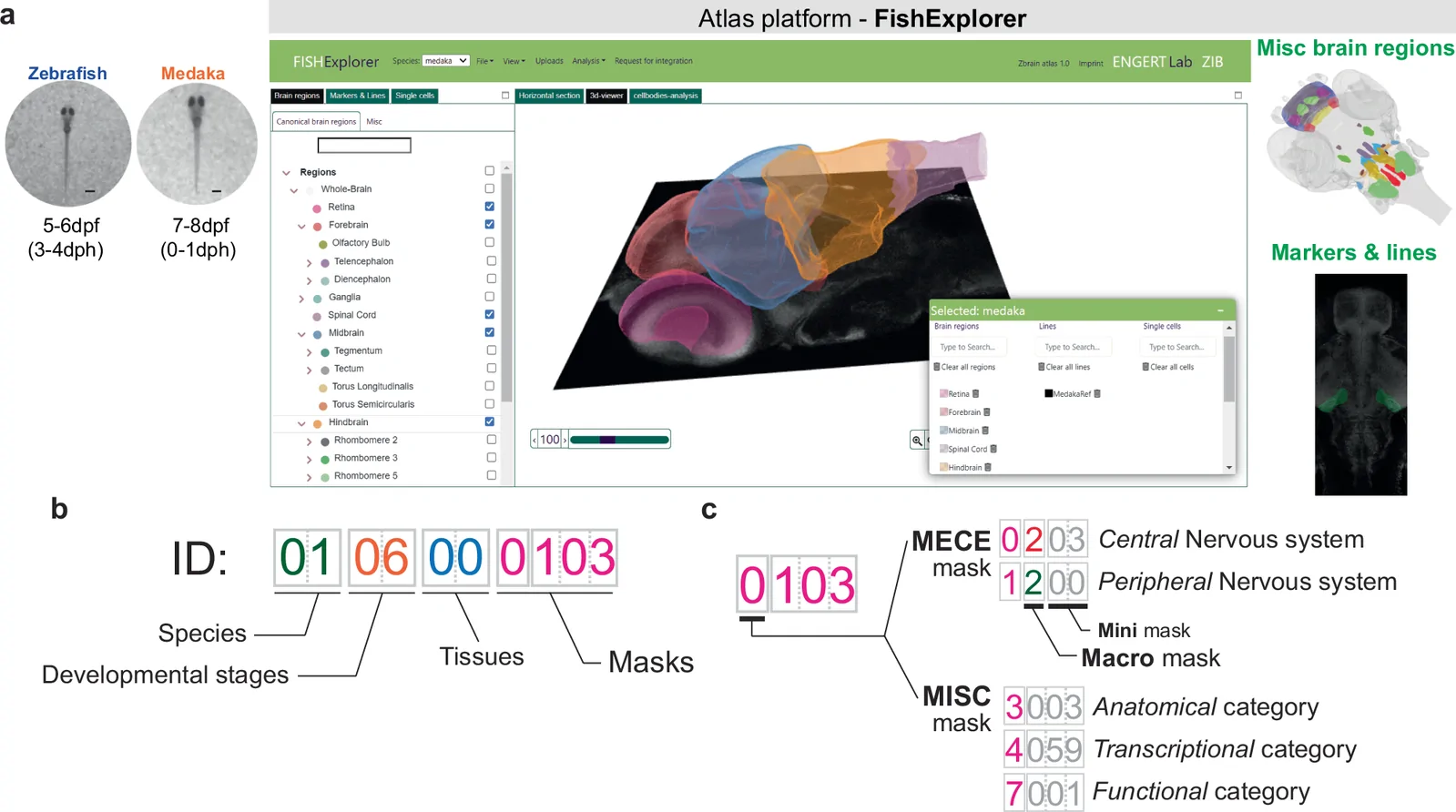
FishExplorer and mask IDs assignments. **a** FishExplorer overview: We extended the existing Z-brain atlas to integrate the finalised medaka dataset and developed the interactive web-based FishExplorer platform (left). Users can switch between species, choose and visualise datasets across different modalities in 2D and 3D (right): MECE and MISC masks can be chosen in the “Brain regions” tab; stained datasets, image stacks of transgenic lines and X-ray dataset can be chosen in the “Markers & Lines” tab. Comparison of whole bodies and heads of teleost larvae, zebrafish (6 days post-fertilisation (dpf)/3 days post-hatch (dph)) and medaka (8 dpf /1 dph). Scale bars: 500 µm. **b** Mask ID assignments: each mask is assigned with a unique identifier, consisting of 10 digits. The two most significant (leftmost) digits, #1 and #2, are reserved for species identifiers. Here we assign “01” for zebrafish and “02” for medaka. Digits #3 and #4 in our current convention are reserved to describe the developmental stage. All of the IDs in this paper have “06” in the third and fourth positions, which means 6 dpf in zebrafish and 1 dph (1 day ‘post hatch’, which corresponds to 6 dpf in zebrafish) in medaka. Digits #5 and #6 are reserved for describing different tissue types, such as “00” for the nervous system. **c** Last 4 digits for mask identifiers: the first digit (#7) codes for MECE masks, taking values between 0 and 2, where “0” indicates the CNS, “1” indicates PNS and “2” is still unassigned. Digit #8 codes for the MECE ‘macro’ mask. The remaining two digits, #9 and #10, are reserved for ‘mini masks’. Values of digit #7 between “3” and “9” are reserved for all MISC masks, with “3” loosely reserved for anatomically defined MISC masks, values “4”, “5” and “6” for transcriptionally defined MISC masks, and values “7”, “8” and “9” reserved for functionally identified MISC masks.

The newly improved and extended FishExplorer (https://fishexplorer.zib.de/) (Fig. 1a), which is a multi-species atlas, offers expertly annotated anatomical masks (‘brain regions’), and fluorescent image volumes labelled with specific stains and transgenic lines (‘markers and lines’) for both zebrafish and medaka. In the interactive website, users can select and visualise multiple anatomical region masks in three dimensions from the list of ‘brain regions’ with arbitrary colours. Also, users can select and visualise imaging stacks in two dimensions and overlay. Users can switch the dataset between zebrafish and medaka with one click, and the corresponding visualisation will show up.

The brain region masks fall into two categories, MECE (Mutually Exclusive and Comprehensively Exhaustive) and MISC (Miscellaneous). MECE masks are hierarchically organised to provide a comprehensive and non-overlapping representation of brain regions: six ‘macro masks’ divide the brain into its major regions. Each macro region contains nested ‘mini masks’ that exhaustively and exclusively tile each macro region. This means that the 113 mutually exclusive mini masks form a 113 dimensional ‘vector’, with the mini masks as an ortho-normal basis set. This structure, leveraging linear algebra, enables whole brain analysis of structure, transcription and function (see below). The MISC masks complement the MECE structure, providing flexibility for additional annotations: They can overlap and form a complementary vector. Their properties are less strictly defined and therefore offer a high degree of flexibility in terms of capturing knowledge about function, transcription, ontogeny, etc. This design allows for flexible expansion, modification and deletion to accommodate diverse research needs.

To ensure systematic organisation and future scalability, we’ve implemented a comprehensive ID system for mask identification (Fig. 1b): each ID consists of 10 digits, accommodating up to 10 billion unique identifiers, which should be sufficient even if we include more species and developmental stages (see “**Methods**”). The last 4 digits are reserved for all of our region masks (MECE and MISC) (Fig. 1c) (see “**Methods**”). The first digit (#7) codes for MECE masks, where “0” indicates the CNS, and “1” indicates PNS. Digit #8 codes for the MECE ‘macro’ mask, with only “1” - “5” currently assigned. The remaining two digits, #9 and #10, are reserved for ‘mini masks’. Values of digit #7 between “3” and “9” are reserved for all MISC masks, with “3” loosely reserved for anatomically defined MISC masks, values “4”, “5” and “6” for transcriptionally defined MISC masks, and values “7”, “8” and “9” reserved for functionally identified MISC masks. Together, this allows for a total of up to 7000 MISC masks to be incorporated. For comparison, our current version contains ~ 200 MISC masks for zebrafish and ~ 80 MISC masks for medaka, numbers we expect to grow rapidly as the community engages and uses the platform.

The following describes in detail how MECE and MISC masks were identified, generated and incorporated into the atlas platform.

We first needed to select zebrafish and medaka fish larvae at comparable stages of development. We chose zebrafish larvae at 6 days post-fertilisation (dpf) and medaka at 1-day post-hatch (dph) (which is 8dpf) (Fig. 1a left) for the following reasons. First, the development of the brain and other organs is comparable at these stages^11^. Second, whereas medaka start swimming immediately after chorion exit at 7 dpf, zebrafish hatch already at 3 dpf but need another few days to show comparable swimming dynamics to medaka at 8 dpf.

Next, we imported existing zebrafish MECE and MISC masks from the Z-brain, an existing zebrafish brain atlas^19,20^, into the FishExplorer. The generation of MECE masks for medaka larvae involved a three-step process:

1. Reference brain generation: We created a reference brain for medaka larvae by staining, imaging and averaging four 8 dpf individuals expressing DsRed under a HuC promoter. These individuals were additionally stained with Hoechst dye and a total-ERK antibody^19^ (Fig. 2a and Supplementary Fig. 1).

**Fig. 2:**
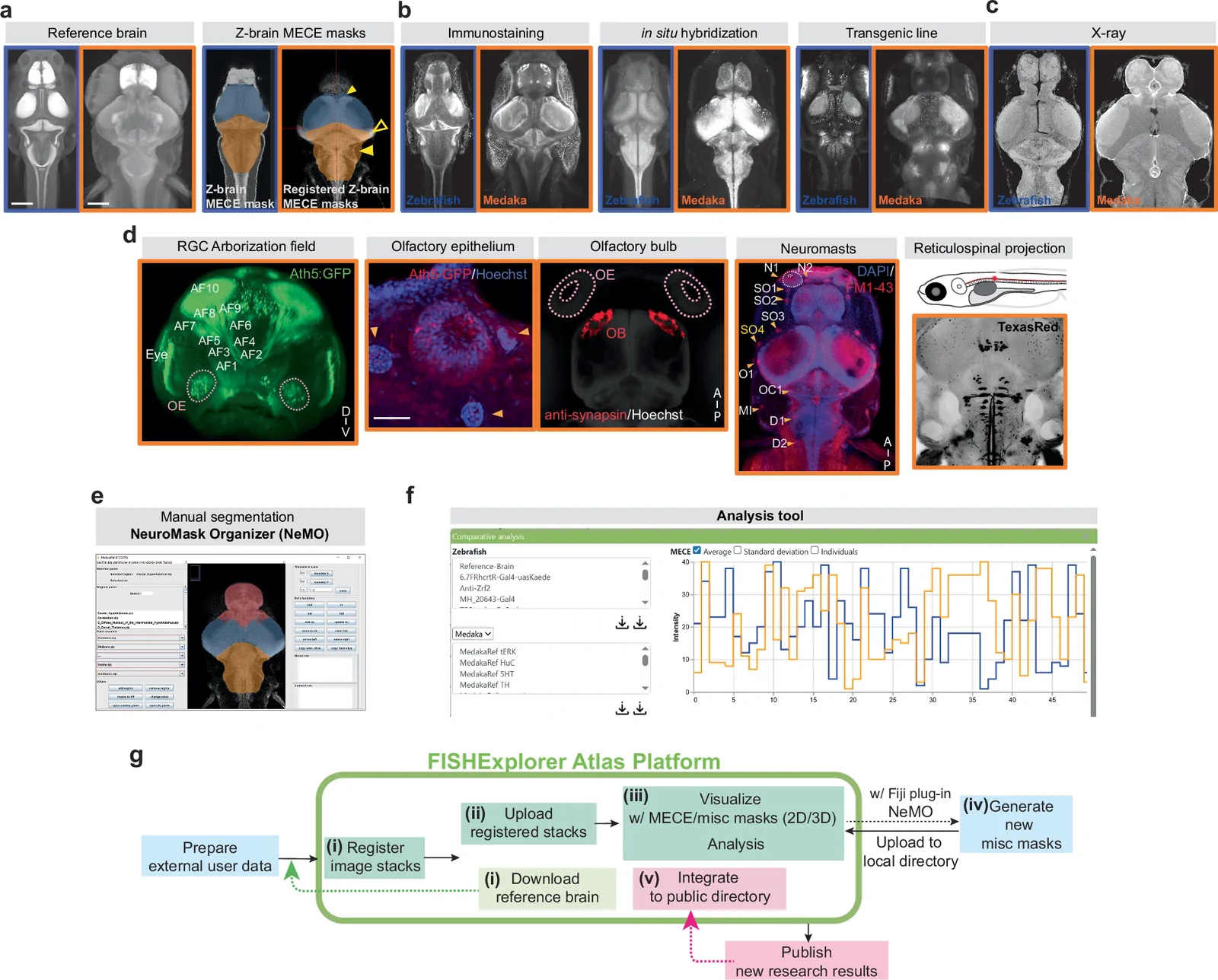
Workflow for creating an atlas for larval teleost fish and analysing user data. **a**–**d** Workflow for creation and validation of the M-brain atlas. **a** Reference brain stacks (anti-tERK) were prepared for medaka and zebrafish (left), and MECE masks from the Z-Brain atlas were registered to the medaka reference brain (right). Maximum projections of registered imaging stacks are shown. Yellow filled and hollow arrowheads indicate over- and under-registration, respectively. **b** Registration was validated using immunostaining (anti-synapsin), in situ hybridisation (*snap25a*) and transgenic lines (Tg(Ath5:GFP)). **c** X-ray imaging was used to confirm anatomical structures. **d** Sensorimotor pathways were used as additional anatomical landmarks. Retinal ganglion cell (RGC) arborisation visualised in Tg(Ath5:GFP) medaka. OE, olfactory epithelium. D, dorsal; V, ventral. Olfactory epithelium and neuromasts (yellow arrowheads) in Tg(Ath5:GFP) medaka and olfactory bulb compartments visualised by anti-synapsin immunostaining. A, anterior; P, posterior. Neuromasts visualised by FM1-43 staining (orange arrowheads). Reticulospinal projections visualised by Texas Red dye backfill. See Supplementary Fig. 6 for registration details. Scale bars: 150 µm in a and 30 µm in d2. **e** The Fiji plug-in Neuro Mask Organiser (NeMO) was used to define and segment medaka MECE masks (see Supplementary Fig. 2). **f** “Analysis” functionality in FishExplorer. **g** Workflow for analysing user data. Users register image stacks using downloadable reference stacks and Python scripts, then upload registered stacks to their private FishExplorer accounts for visualisation with existing MECE and MISC masks. New MISC masks can be generated using NeMO and uploaded to private accounts. Upon publication, users may request the addition of their image stacks and MISC masks to the public database. Light blue boxes indicate activities performed outside the platform; green boxes indicate activities within FishExplorer; pink boxes indicate post-publication data integration.
2. Initial mask registration: We utilised the Advanced Normalisation Tools (ANTs)^21^ to register the Z-brain MECE masks to the new medaka reference brain. This process produced a set of coarse preliminary medaka MECE regions (Fig.2a, right). However, while the overall quality of this initial registration was adequate for large region masks, over- and under-estimations could be observed at the boundaries, and most of the small brain regions were not accurately registered.
3. Expert refinement: All medaka MECE regions were refined using local information from the three colour channels of the reference brain and marker-gene expression patterns (Fig. 2b, d). To facilitate this refinement, we developed a custom Fiji plug-in, called Neuro Mask Organiser (NeMO), for segmenting brain regions, which we are making freely available to the community (see **Materials and Methods**) (Fig. 2e and Supplementary Fig. 2). Specifically, we implemented a semi-automated technique that combines manual labelling with a machine learning approach: A 2D U-Net network^22^ was trained to segment the large regions in medaka (Supplementary Fig. 3,"**Methods**”). The deep learning-based predicted masks were then fused, and the final region masks were generated using the NeMO plug-in. To ensure the quality of small anatomical regions, we validated their location and size using expression patterns (see Supplementary Table 1, Fig. 2b, d and Supplementary Fig. 4, labelling methods in Supplementary Table 4 for both species). Finally, we generated an X-ray dataset as an orthogonal approach to fluorescence imaging, which we used to identify neuromasts and also proved useful for the visualisation of all non-neuronal tissues (Fig. 2c and Supplementary Fig. 5). In the FishExplorer platform, we developed a cross-species “Analysis” functionality that will facilitate quantitative comparison (Fig. 2f).

### Data integration workflow and community engagement to FishExplorer

The FishExplorer is designed to encourage interactive engagement and serve as a universally accessible repository for published data across many aspects of teleost fish research. In the following, we outline the process for users to engage with the platform and upload their own data volumes (Fig. 2g).

First, users download the reference brain and register their dataset to that volume. A tutorial of how to do registration is available at the website (FishExplorer: https://fishexplorer.zib.de/). Registered data volumes then need to be uploaded to the user’s ‘private local’ repository (Supplementary Fig. 6d), which then allows for the use and application of the FishExplorer’s visualisation and analysis tools to their uploaded data (Fig. 2g). If needed, users can draw new MISC masks with the Fiji plug-in NeMO (a tutorial is available, see Supplementary Fig. 2). Upon publication in a peer-reviewed journal, final results will be transferred and integrated into the FishExplorer’s ‘public’ repository making them accessible to the community (Fig. 2g and Supplementary Fig. 6f).

### Cross species comparison and volumetric analysis

**Fig. 3:**
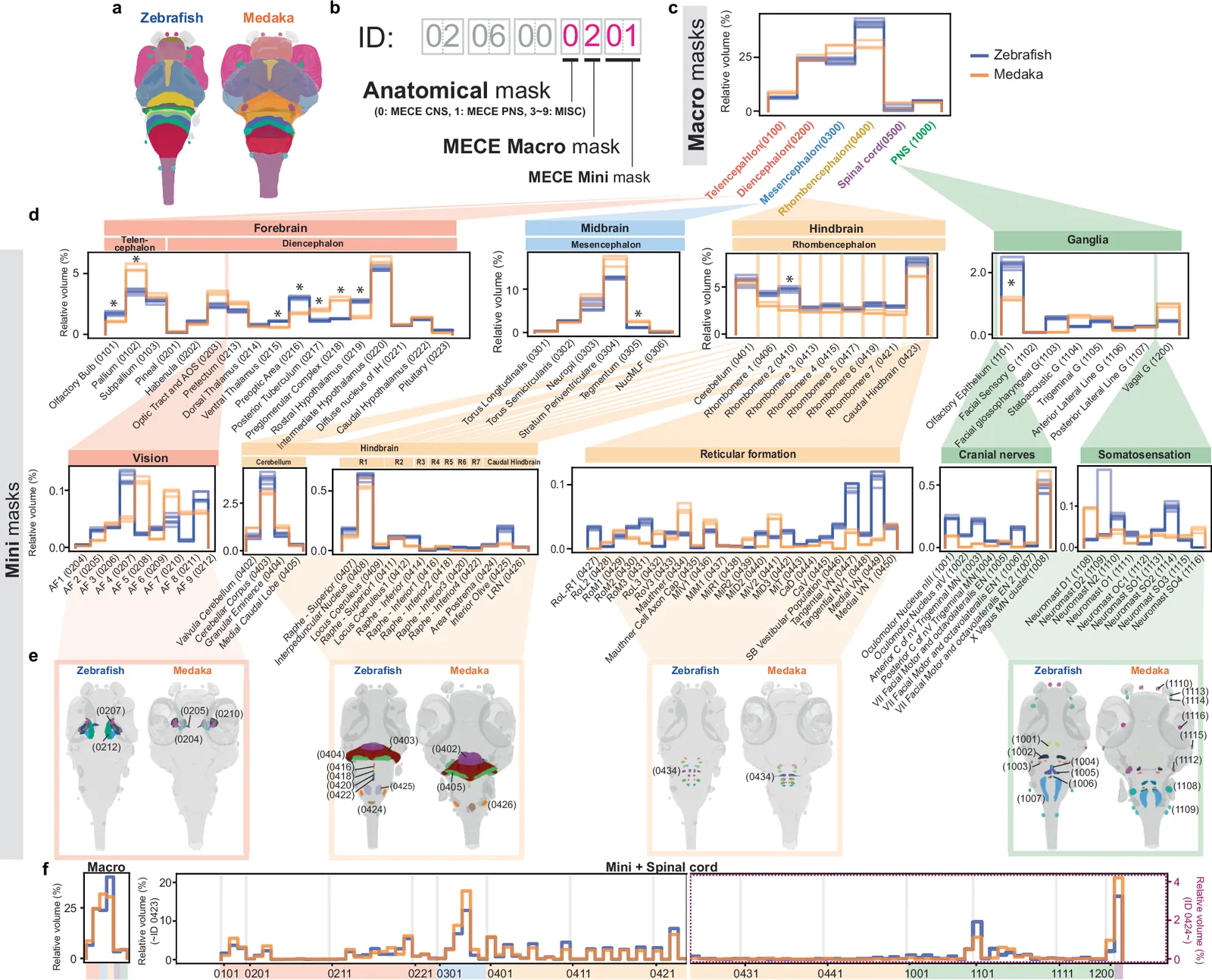
Volumetric comparison of MECE region masks in zebrafish and medaka larvae. **a** 3D representation of MECE region masks in zebrafish (left) and medaka (right). **b** MECE mask IDs. The first digit indicates the CNS (0) or PNS (1), the second indicates the corresponding macro mask, and the last two digits identify mini masks. **c**, **d** Relative volumes of MECE masks in zebrafish and medaka, shown as Manhattan plots (zebrafish, *n* = 7; medaka, *n* = 3). Numbers following mask names indicate MECE mask IDs. **c** Macro masks: telencephalon (0100), diencephalon (0200), mesencephalon (0300), rhombencephalon (0400), spinal cord (0500) and ganglia (1000). **d** Nested mini masks. AOS, accessory optic system; IH, intermediate hypothalamus; AF, arborisation field; LRN, lateral reticular nucleus; R1, rhombomere 1 (R2–R7 similarly); G, ganglia; SB, spinal backfill; VN, vestibular nucleus; C, cluster; MN, motor neurons; EN, efferent neurons. Asterisks indicate masks showing both statistical significance (FDR-*q* < 0.05, two-sided Mann–Whitney *U* test) and a substantial biological difference (fold change > 1.5 or < 0.67). Exact *P* and FDR-*q* values, *U* statistics, rank-biserial effect sizes and 95% confidence intervals are provided in the Source Data file. Masks comprising < 1% of whole-brain volume were excluded from statistical analysis. (**e**) Selected MECE mini masks from (**d**) in the reticular formation and neuromasts. Reticular formation: M, Mauthner cell; RoM, rostral Mauthner cell clusters; MiM, middle cells; MiV, ventral cells; MiD, dorsal cells; MiT, T-shaped axon cells; MiR, rostral cells; MeM, medial mesencephalic projection; MeLm, lateral-medial mesencephalic projection; MeLr, rostral mesencephalic projection; MeLc, caudal mesencephalic projection; RoV, ventral rhombencephalic projection; RoL, lateral rhombencephalic projection; CaD, dorsal caudal cells; CaV, ventral caudal cells; NucMLF, nucleus of the medial longitudinal fasciculus; V, vestibular clusters. Neuromasts: N, nasal; SO, supraorbital; O, optic; OC, occipital; MI, middle; D, dorsal. **f** Volumetric vectors summarising the six macro masks, 113 mini masks and spinal cord for brain-wide comparison between species. The rightmost box corresponds to the right vertical axis.

We defined the MECE masks in both zebrafish and medaka based on the conserved brain areas across vertebrates (Fig. 3a). MECE macro masks identify the telencephalon (ID: 0100), the diencephalon (ID: 0200), the mesencephalon (ID: 0300), the rhombencephalon (ID: 0400), the spinal cord (ID: 0500) and the Ganglia (ID: 1000) (Fig. 3b, c). MECE mini masks are nested within MECE macro masks. Their individual identities and macro mask assignments are colour-coded and outlined in Fig. 3d, e.

We use the following convention for ID assignment: the ID value follows a rough rostral-to-caudal rule, i.e., the most rostral regions were assigned the smallest values, which increase as one moves towards the tail of the fish. For example, the olfactory bulb has ID 0101, and the caudal hindbrain has ID 0423. The “0” in the first place indicates CNS and a “1” PNS, and the following three digits again follow a rostro-caudal ordering rule. For example, the olfactory epithelium and the vagal ganglia, which are part of the PNS, have ID 1101 and ID 1200, respectively.

To leverage and test the capacity of FishExplorer, we first performed a volumetric comparison of MECE regions between zebrafish and medaka. To this end, we plotted the MECE volumes of zebrafish and medaka over their unique identifiers in the form of ‘Manhattan plots’ (Fig. 3c,d and Supplementary Table 1). This approach allows direct comparison of relative brain-region volumes and revealed that the midbrain (ID: 0300), especially the tectum stratum periventricular (ID: 0304), is larger in medaka, whereas the hindbrain (ID: 0400) is larger in zebrafish (Fig. 3c). Given the limited sample size (zebrafish, *n* = 7; medaka, *n* = 3), we highlighted regions showing both statistical significance (FDR-adjusted *P* < 0.05, exact two-sided Mann–Whitney *U* test) and a substantial difference in magnitude (fold change > 1.5 or < 0.67). Exact *P-*values, FDR-adjusted *P-*values, *U* statistics, rank-biserial effect sizes and 95% confidence intervals are provided in the Source Data file.

Almost all MECE mini masks were identified in both zebrafish and medaka. The only exceptions were a medaka-specific pair of neuromasts above the midbrain (neuromast SO4, ID: 1116) and a zebrafish-specific reticulospinal cluster (MiD2, ID: 0439) in the hindbrain (Supplementary Table 1). Many MECE mini masks showed substantial divergence in relative volume between the two species. Figure 3f summarises the six macro masks and 113 mini masks as volumetric vectors, enabling brain-wide quantitative comparison between species. Together, these analyses demonstrate the utility of FishExplorer for detailed quantitative comparisons of brain structure across species.

### Cross species comparison of labelled neural subclusters through MECE masks

**Fig. 4:**
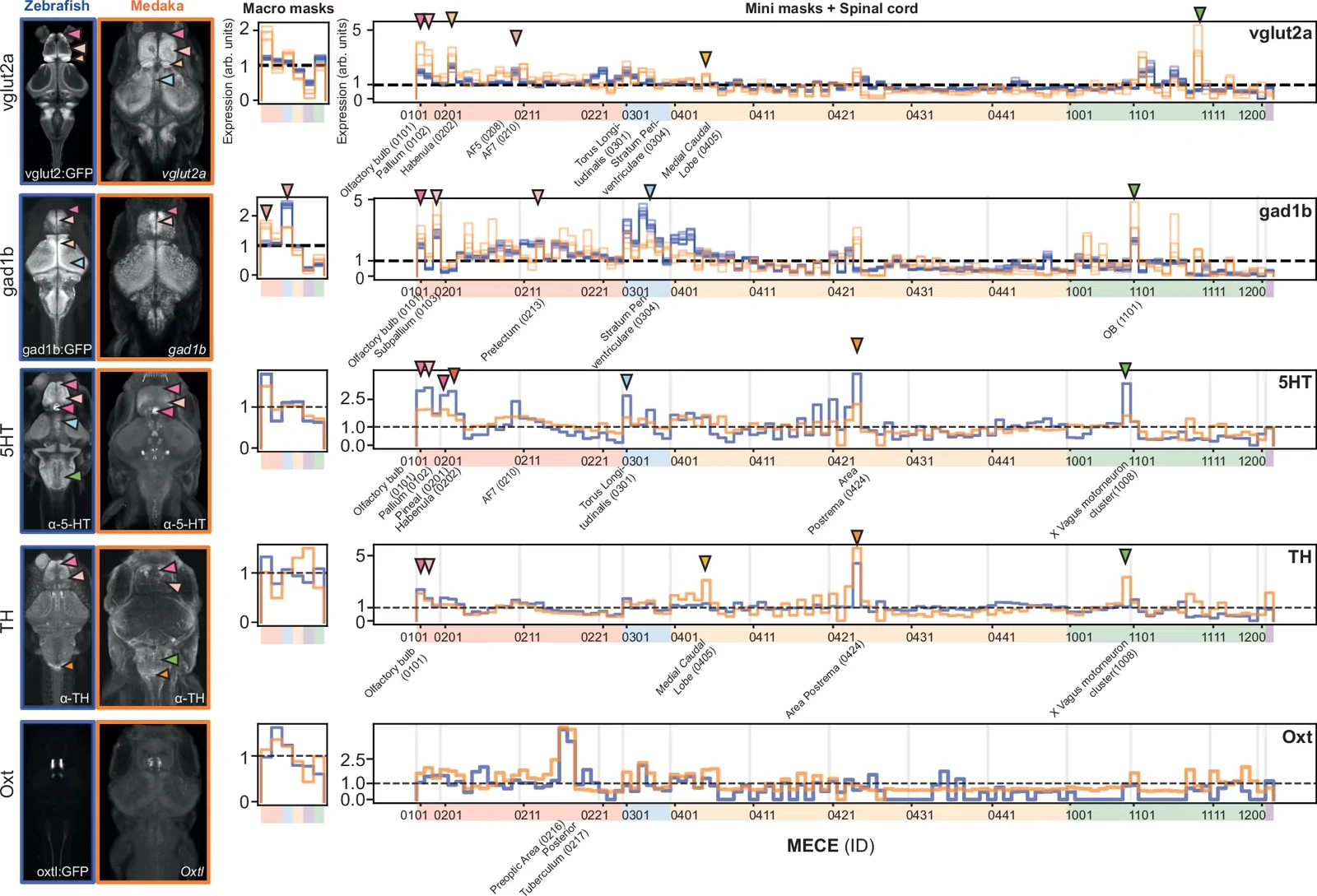
Comparison of selectively labelled subclasses of neuronal populations. Expression of basic neurotransmitters and neuropeptides (Left). From the top to the bottom, maximum projection of imaging stacks of *vglut2a, gad1b*, 5-HT, TH, and *OXT* are shown. 5-HT: 5-hydroxytryptamine, TH: tyrosine hydroxylase, OXT: Oxytocin, *vglut2a*: vesicular glutamate transporter 2a, *gad1b*: glutamate decarboxylase 1b. Maximum projections of registered imaging stacks (generated outside of the platform) are shown on the left. Regions that display strong expression and/or diverged expressions between species are highlighted with coloured arrowheads that correspond to those in the right panel, and the name of the masks are shown below plots. (Right) Manhattan plot of intensities of gene expression in zebrafish (blue) and medaka (orange) across MECE macro masks (left) and MECE mini (+ spinal cord) masks (right). Individual lines in *vglut2a* and *gad1b* indicate individual fish. Zebrafish (*vglut2a*): *n* = 8, zebrafish (*gad1b*): *n* = 10, medaka (*vglut2a*): *n* = 5, medaka (*gad1b*): *n* = 4. For the 5HT, TH, and Oxt analyses, average image stacks from the Z-Brain atlas^19^ were used. Because the original individual datasets are not publicly available, biological variability could not be estimated, and error bars are not shown for these markers. Source data are provided as a Source Data file.

The expression patterns of neurotransmitters, neuropeptides or other transcriptional properties that help to classify cell types are all useful features for identifying homologies across brain regions. To test the effectiveness of our approach, we combined staining of brain tissue (using immunohistochemistry and in situ hybridisation) with our quantitative ‘Manhattan plot’ strategy. To that end, we selected the following six major neurotransmitters: serotonin (5-HT)^23^, dopamine (TH)^24^, noradrenaline (TH)^25^, oxytocin (Oxt)^26^, glutamate (vglut2a), and GABA (gad1b). For 5HT, TH, and Oxt, we analysed the corresponding average image stacks from the Z-Brain atlas^19^. We plotted the fluorescence values normalised by the median value across all regions for both species (Fig. 4). We note that dopamine and noradrenaline expression are analysed jointly through staining for tyrosine hydroxylase (TH), an enzyme that is critical for the biosynthesis of both transmitters^24^.

By normalising with the mean signal value across all MECE regions, we found that the expression patterns of most genes are similar in zebrafish and medaka, but some distinct differences between the two species were detected (all marked by coloured arrowheads). Glutamatergic signals in the habenula (ID: 0202) and the arborisation field 7 (AF7, ID: 0210) are stronger in zebrafish than in medaka. The lower level of 5HT expression in the forebrain of medaka compared to zebrafish (Fig. 4, third row, IDs: 0101, 0102, 0201, 0202) is likely explained by a difference in the quality of the antibody staining (see **Discussion**). This indicates possible deficiencies and the need for careful calibration of staining, imaging, and analysis.

### Cross species comparison of labelled neural subcluster within MISC masks

To enable the integration of specific cell-type classes, transcriptionally defined regions or functionally distinct areas, which might not readily comply with the rigid and fixed MECE framework, we introduced the second category of miscellaneous (MISC) masks. These MISC masks complement the MECE regions and serve as preserved descriptors for arbitrary subregions of particular interest. Notably, MISC masks do not have to comply with the MECE principle and can therefore overlap with each other. MISC masks can be generated by the user, imported into the FishExplorer for analysis and permanently integrated into the public repository upon publication (Fig. 2e). The definition of MISC masks is based on the respective research questions and can also depend on experimental restrictions. MISC masks are useful to quantitatively describe and annotate any region of interest, and they can serve to extract and quantify the label intensity in the same regions in any experimental context. We anticipate that the number of MISC masks in both species will grow continuously through contributions from the teleost community, as new information on fish neuroanatomy, new cell type specifications and new functionally identified regions are discovered at a rapid rate.

**Fig. 5:**
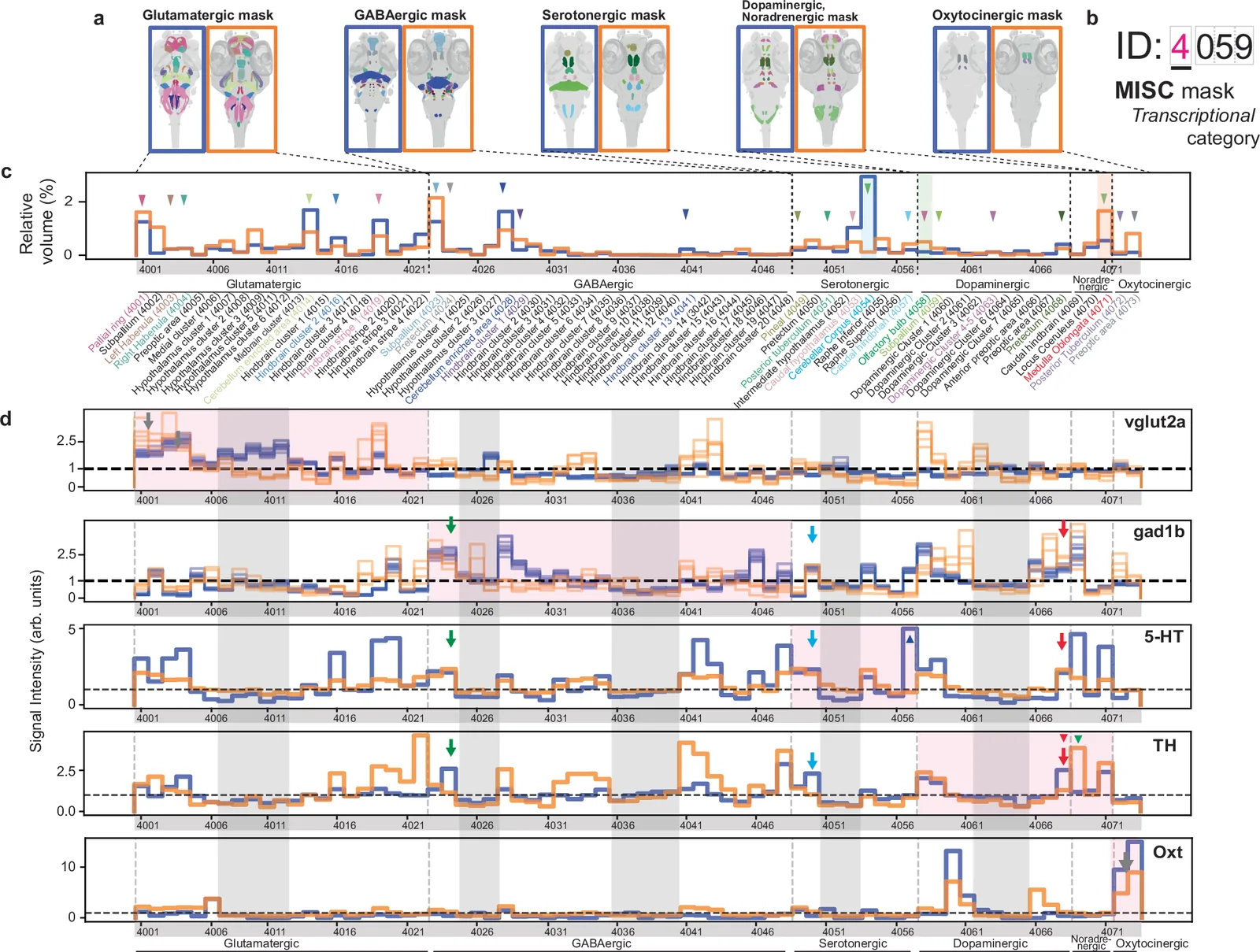
Comparison of selectively labelled subclasses of neuronal populations using MISC masks. **a** Miscellaneous (MISC) masks of brain regions expressing neurotransmitters. MISC masks were generated based on the location of the cell bodies of neurons. Blue outline: zebrafish; Orange outline: medaka. **b** MISC masks IDs. The leftmost digit (#7) ‘4’ indicates MISC masks that are defined by transcription. **c** Volumetric comparison of MISC masks. Blue line: zebrafish; orange line: medaka. Coloured arrowheads and the name below the plot correspond to the MISC masks in (**a**). **d** Manhattan plots of signal intensity of neurotransmitters across MISC masks. Average intensity signals of neurotransmitters in each mask was calculated and normalised by the average value in the whole brain. Pink shades indicate the MISC masks corresponding to the neurotransmitters. Grey shades indicate the MISC masks that show low levels of signals, which we think happened because of a lack of staining or imaging noise. Green, blue and red arrows indicate that the pretectum MISC masks that include GABAergic, serotonergic and dopaminergic neurons, or neurons that co-express these three neurotransmitters. Blue, red and green arrowheads indicate MISC masks that show divergent expression between zebrafish and medaka, mentioned in the text. Individual lines in *vglut2a* and *gad1b* indicate individual fish. Zebrafish (*vglut2a*): *n* = 8, zebrafish (*gad1b*): *n* = 10, medaka (*vglut2a*): *n* = 5, medaka (*gad1b*): *n *= 4. For the 5HT, TH, and Oxt analyses, average image stacks from the Z-Brain atlas^19^ were used. Because the original individual datasets are not publicly available, biological variability could not be estimated, and error bars are not shown for these markers. Source data are provided as a Source Data file.

First, we imported ~ 200 existing zebrafish MISC masks from the Z-Brain Atlas^19^, and generated 73 new MISC masks in medaka using expert knowledge and the NeMO tool (Fig. 5a, see Supplementary Fig. 2). MISC masks are identified by the digits “3” - “9” in the fourth least significant digit of our ID, which corresponds to #7 (Figs. 1b, c, 5b). For example, digit value “4” at position #7 indicates that these MISC masks were generated using a *transcriptional* label, and the remaining three digits are incremented following a rough rule based on the previously identified neurotransmitter (IDs 4001 − 4022 describe the glutamatergic cluster, IDs 4023 − 4048 describe the GABAergic cluster, IDs 4049 − 4057 describe the serotonergic cluster, IDs 4058 − 4068 describe the dopaminergic cluster and IDs 4069 − 4071 describe the three noradrenergic clusters). We further distinguish between the two *primary* neurotransmitters Glutamate and GABA and four *modulatory* neurotransmitters, which in our case are Serotonin (5-HT), Dopamine (TH), Noradrenaline (TH), and Oxytocin (OXT). We note that dopamine and noradrenaline are stained together by a single antibody against tyrosine hydroxylase, and the separation is accomplished by past assignment taken from the literature and by expert annotations. Figure 5a shows 73 MISC masks common to both species, and roughly grouped into five classes identified by their association with a known neurotransmitter type, distributed over the whole brain of zebrafish (blue) and medaka (orange). Primary neurotransmitters are shown up to ID 4048, and modulatory neurotransmitters are shown from ID 4049 to 4073. All six classes are divided by vertical dashed lines in Fig. 5c, d.

We first used the MISC masks to compare the relative volumes between zebrafish and medaka for these particular clusters (Fig. 5c). Coloured arrowheads point to MISC regions corresponding to the similarly coloured areas in the ‘fish heads’ on top (Fig. 5a). Several of these highlight the divergence in relative brain region volumes between zebrafish and medaka. MISC masks that differ more than twofold in size are highlighted by a shaded background. For example, the medaka brain has a much smaller serotonergic mask in the cerebellar corpus (ID 4054), a much larger dopaminergic mask in the olfactory bulb (ID 4058) and also a larger noradrenergic mask in the medulla oblongata (ID 4071) (Fig. 5a, b, MISC region labels highlighted in red, green and blue, respectively).

Second, we used the MISC approach to quantitatively compare transcriptional expression patterns within and across the respective medaka and zebrafish masks (Fig. 5d).

We note that the assignment of the neurotransmitter identities to these MISC regions was based on ‘judgement calls’ by the annotator who originally generated the label, and is often based only on a subjective prevalence of the particular neurotransmitter type, and it often depended on a particular staining or transgenic approach.

The lack of consistently high expression in each predicted group of MISC regions (areas shaded in pink) highlights the propensity for false negatives in the respective labels. Specifically, a low value for a MISC region in a ‘pink’ category indicates a mismatch between the expected ‘ground truth’ established by previous experts and the reality of low label intensity in our measurement. Such a discrepancy suggests and exposes potential flaws in the specific staining protocols, or indicates complications in the normalisation and image analysis procedures that turn a brain-wide fluorescent label into a quantitative metric for a biological parameter. For example, all masks between ID 4036 and 4040 show very little signal. This is likely to be explained by the tiny volume sizes of these regions (Fig. 5c), again highlighting the sensitivity of the automated extraction protocol to imaging noise, background fluctuations, and sparse labelling, and emphasising the need for caution when applying these analysis methods. Masks ID 4007 to 4012 within the glutamatergic cluster also show surprisingly little staining. We believe this is due to their far ventral location in the hypothalamus, which compromises fluorescent imaging in fish that are mounted upright under the objective. IDs 4025 − 4027 in the GABAergic cluster, IDs 4051 − 4053 in the 5-HT cluster and IDs 4063 − 5065 in the TH cluster are also found in the ventral hypothalamus and are similarly compromised. The labels of all masks showing such an unexpected lack of staining are greyed out in Fig. 5d. We note that such a ‘mismatch’ between predicted and actual staining results can be used as a sensitive and useful control for a wide range of staining methods, providing a realistic estimate of the true reliability of each protocol.

The frequent peaks outside of the expected regions are not so surprising. The notion that certain brain regions express only one neurotransmitter and no others, is clearly false in the vast majority of cases. While some regions may be biased towards one particular neurotransmitter, several transmitters are usually co-expressed in each region. This is certainly the case for glutamate and GABA. Therefore, we expect that clusters that are assigned to a particular neurotransmitter MISC group (shaded in pink in Fig. 5d) will also contain signals for other transmitter types (see corresponding unshaded areas).

We find that the three modulatory clusters all contain significant GABAergic labelling (with the exception of OXT), while they are largely devoid of glutamatergic signals (Fig. 5c, top two rows, ID 4049 onwards). This suggests that these modulatory regions contain little glutamate staining, possibly because excitatory drive is provided by long-range projections from other regions, while inhibitory balance is provided by local inhibitory interneurons located nearby within the same region. Thus, comparing expression levels within a column, i.e., the relative labelling of a given MISC region for different stains, can reveal the mixed expression of different transmitters in each region of interest.

For example, within the pretectum three distinct, largely overlapping, MISC regions had previously been identified - ‘Pretectum GABAergic cluster ID 4024’, ‘Pretectum serotonergic cluster, ID 4050, and ‘Pretectum dopaminergic cluster, ID 4068 (green, blue, and red arrows respectively, see also Supplementary Fig. 7) - which, when applied to the appropriate labelled fluorescent stacks, extracted the majority of correct labels. As expected, the oxytocinergic masks extracted very strong and selective signals from a fish labelled by in situ hybridisation of the oxytocin ligand in both species. The significant OXT signal found in the dopaminergic cluster is explained by its significant overlap with oxytocinergic masks in the diencephalon.

Next, we used this approach to compare zebrafish and medaka. Comparing blue and orange MISC masks, it is clear that many regions show significant divergence between the species. However, caution needs to be exercised in interpreting these differences, as they do not necessarily reflect a difference in the underlying biology. As mentioned above, absolute staining levels are often confounded by experimental context (**see Discussion**). In the following, we state some observations that are probably worth pursuing. First, the serotonergic mask in the caudal hindbrain is five times larger in zebrafish than in medaka (Fig. 5d, third row, ID: 4057). Second, the dopaminergic signal intensity in the pretectum is twice as strong in zebrafish as in medaka (Fig. 5d, fourth row, red arrowhead, ID: 4068). Third, the noradrenergic caudal mask in the hindbrain is three times larger in medaka (Fig. 5d, fourth row, green arrowhead, ID: 4069).

In general, we view such data to be most useful in the generation and preliminary validation of specific hypotheses. Their specific strength lies in their ability to provide a general and brain-wide overview of the concerted and distributed expression of a wide range of different peptides, neurotransmitters and expression patterns, allowing these patterns to be analysed across different species, mutants or developmental stages (see **Discussion**).

### Comparison of neural activity patterns

**Fig. 6:**
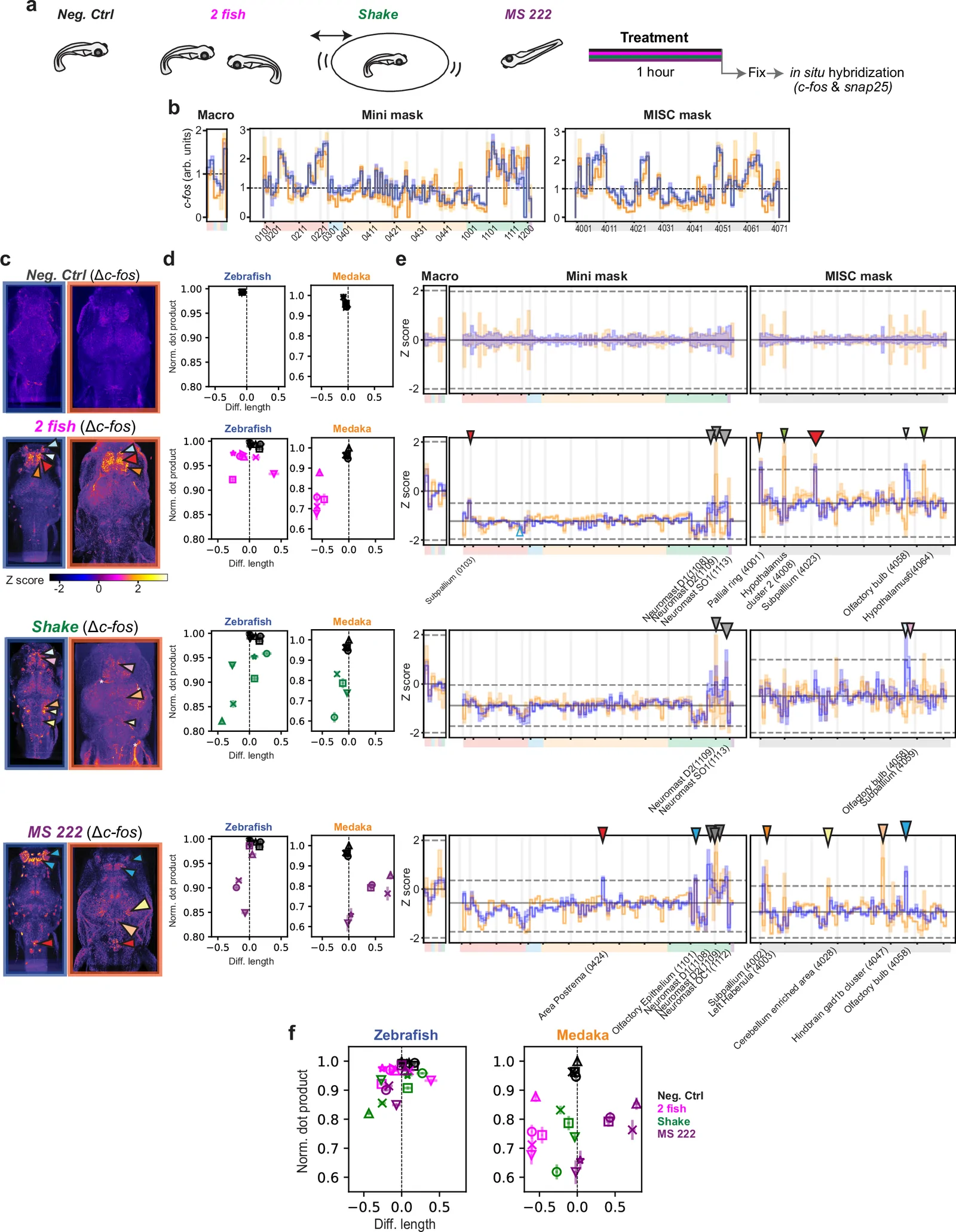
Comparative analysis of functional response properties in the teleost brain. **a** Experimental design to analyze functional divergence in the brain across several treatments. After one hour of treatment, either ‘2 fish’, ‘shake’, ‘MS222’ or ‘negative control’, fish larvae were fixed, and in situ hybridisation was performed for *c-fos* and *snap25a* genes. ‘2 fish’ experiment, zebrafish: *n* = 6, medaka: *n* = 5; ‘shake’ experiment, zebrafish: *n* = 6, medaka: *n* = 4; ‘MS222’ experiment, zebrafish: *n* = 7, medaka: *n* = 6, Neg. ctrl., zebrafish: *n* = 5, medaka = 5. **b** Visualisation of neural activity with *c-fos* staining of negative control. Manhattan plots of *c-fos* signals across MECE macro masks, MECE mini masks and MISC masks are shown. Blue line: zebrafish; orange line: medaka. *c-fos* signals were normalised by the expression of pan neuronal gene (*snap25a*), then normalised across brain regions by being divided by the average of *c-fos* signals across the whole brain. Standard deviations were displayed in shades. **c** Visualisation of divergent neural activity from negative control. Mock trials (first row), 2-fish treatment (second row), shaking treatment (third row), MS222 treatment (fourth row) are shown. Maximum projections of raw data (generated outside of the platform) are shown on the left. OB: olfactory bulb. H: habenula. HT: Hypothalamus. SPe: Striatum Periventriculare. NuMLF: Nucleus medial longitudinal fissure. OE: the olfactory epithelium. **d** ‘Phase space population’ metrics. The vertical axis indicates the ‘dot product’ and the horizontal axis indicates the ‘differential size’ of vectors between negative control and vectors of either mock trials (black, first row), 2-fish treatment (magenta, second row), shaking treatment (green, third row), MS222 treatment (purple, fourth row). Different shapes of markers show different individuals. **e** Divergent neural activity in each mask is plotted in z-score. Blue line: zebrafish; orange line: medaka. Dotted lines indicate either + 2 or − 2 standard deviations. Significantly high or low divergent activity are highlighted with arrowheads, and corresponding areas are indicated in (**c**). **f** The quantitative comparison of global neural activity patterns with ‘phase space population’ metrics across multiple treatments and species. Black: negative controls, magenta: 2-fish treatment, green: shaking, purple: MS222 treatment. Source data are provided as a Source Data file.

To leverage the MECE and MISC masks in a neurophysiological context, we used *c-fos* staining to compare temporally averaged neural activity when animals were stimulated by different sensory modalities. Fish were either subjected to a conspecific stimulus by having them swim in pairs, placed on a shaker for sixty minutes to drive somatosensory and vestibular inputs, or they were subjected to full anaesthesia by immersion in 100 µg/ml of tricaine methanesulfonate (MS222). For each condition, we separated a similarly sized ‘sibling control’ that was allowed to just swim spontaneously for one hour (negative control) (Fig. 6a and Supplementary Fig. 8).

After these respective treatments, all animals were fixed in paraformaldehyde, and activated cells were visualised by the expression of the immediate early gene, *c-fos*, by in situ hybridisation and normalised by the expression of the pan-neuronal gene, *snap25a* (Fig. 6a). When we compared *c-fos* expression in negative controls across zebrafish and medaka (Fig. 6b), we found that they were remarkably similar. We also confirmed that the variance between individuals within the same species was low.

To distinguish between global intensity changes from differential activity modulation, where concerted excitatory and inhibitory effects could result in distinct activation patterns with no net change in the brain-wide average intensity, we used the following approach. First, we calculated two global MECE mini-mask vectors, one for the ‘negative control’ and one for the ‘treatment’ fish, where each of the 104 elements contains the *c-fos* average (normalised by *snap25a*) of each mini MECE region. Each of these two vectors represents the concerted activity of all regions as an activity point in a 104-dimensional phase space, similar to the common representation of phase space in an activity population vector of 104 different spiking neurons.

The normalised ‘dot product’ of the ‘negative control’ vector with the ‘treatment’ vector gives a quantitative metric for the similarity between the two activity patterns, while the difference in the absolute length of the two vectors gives an indication of the global change in activity. To visualise these two - independent - possible changes in activity across the fish brain, we plot the ‘dot product’ on the vertical axis and the ‘differential length’ on the horizontal axis as a pairwise comparison for each combination of negative control and treatment.

To illustrate this process, we first show a ‘mock’ trial where we randomly divide the 8 negative control animals into two groups and assign one to the control and the other to the treatment (Fig. 6 first row). We found that the normalised ‘dot product’ in the ‘mock’ trial was close to 1, and the ‘differential length’ was close to 0 (Fig. 6d first row), confirming the validity and reliability of the approach. We also checked the differential expression in each element of the vectors in the ‘mock’ trial by calculating the z-score (Fig. 6e first row), which gives us a sense of variance across all regions.

Next, we applied this approach to the different stimulation protocols. In the ‘2-fish social stimulus’ protocol (Fig. 6c–e), the negative ‘differential length’ in the treatment group (magenta points are shifted to the left) shows that the social context significantly reduces global activity in medaka but not in zebrafish (Fig. 6d). Also, the more significant drop in the ‘dot product’ in medaka suggests that neural activity in this species is more affected by other fish swimming nearby.

Detailed inspection of the Manhattan plot (Fig. 6e) reveals that social stimulation leads to strong activation of the telencephalon in both species, including the olfactory bulb (ID 4058, green arrowheads) and the subpallium (ID 0103, 4023 red arrowheads). The prevalence of activation in olfactory regions (IDs 4058, 4059) suggests that olfactory conspecifics may play a prominent role in these social interactions – a phenomenon that has previously been confirmed by several studies^27,28^.

Other regions that are suppressed by the social stimulus are indicated by empty arrowheads pointing to values below a z-score of − 2. Such strong inhibition, possibly induced by the social context, could be triggered by the specific activation of inhibitory populations. Indeed, we find that in both zebrafish and medaka, GABAergic neurons in the subpallium (ID: 4023, wide red arrowhead) are strongly activated in this context. This is in line with a previous study that identified GABAergic neurons in the same region that were shown to be activated by social olfactory stimuli, and that target and suppress a neuronal population in the hypothalamus^27^. The general activation of inhibitory neurons may also explain the strong reduction we see in several other hypothalamic regions (IDs 4008, 4064, light-blue hollow arrowheads). We note that medaka show very similar activity phenotypes, suggesting that the same circuits may be involved in both, distantly related, teleost species.

Vibration stimuli are delivered by placing the animals on a standard laboratory ‘shaker’ for 60 min, causing enhanced stimulation in visual, somatosensory and vestibular pathways, in addition to inducing moderate levels of stress. Using our ‘phase space population’ metric, we quantified stimulus-related activity across all mini MECE regions in zebrafish and medaka.

As predicted, we found a wide variance in ‘differential length’ in zebrafish, indicating that net global activity can either go up or down in individual animals. Medaka, on the other hand, shows slight but consistent suppression (Fig. 6d), which might suggest some form of habituation in this particular context. Nonetheless, the distinctly larger drop in the ‘dot product’ in medaka suggests a larger variance in neural activity across all regions. Zebrafish showed a strong activation of the neuromast (IDs: 1109, 1113, grey arrowheads) that is milder in medaka, which may indicate a difference in flow sensing algorithms^29^ between the two species. All these differences motivate future studies to investigate potential differences in the sensitivity of these two modalities between species. This activation in neuromasts is consistent with the need for constant flow sensing and corrective swimming manoeuvres throughout treatment.

MS222 is a general anaesthetic used in teleost fish, and its application should reduce activity across the brain. In line with this expectation, in zebrafish, we find a global reduction in all MECE and MISC regions (Fig. 6c, d), with the exception of the olfactory system (IDs 0101, 1101, 4058, blue arrowheads), which is likely caused by a direct activation through the compound (Fig. 6e). We also see a specific activation of the area postrema (AP) in zebrafish (ID: 0424, red arrowhead), suggesting that even under general anaesthesia this noradrenergic nucleus is still activated^19^. We note that such selective activation of the AP by an anaesthetic compound may confound behavioural and neurophysiological studies related to these neuroactive compounds. Interestingly, we detected activation in neuromasts (IDs: 1108, 1109, 1112, grey arrowheads), but we didn’t detect activation in AP in medaka.

The quantitative comparison of global neural activity patterns across multiple treatments and species afforded by our ‘phase space population’ metric (Fig. 6f) allows for the generation of several testable hypotheses. The fact that evoked brain-wide neural activity patterns showed more variance in medaka than in zebrafish (suggests that these species may process sensory stimuli in a distinctly different fashion, a phenomenon whose elucidation will require much detailed investigation in the future.

### Comparison of the telencephalon in teleost larvae

The FishExplorer allowed us to analyse larval teleost brains and address questions in evolutionary diversity. One of the brain areas with the highest diversity in vertebrates is the telencephalon^30^. The mammalian telencephalon has been extensively studied; it includes the amygdala, hippocampus and neocortex. In adult teleost species, previous studies unveiled a large diversity of anatomical regions within the telencephalon^31,32^. However, the precise function and homology of these regions in teleosts in general, and in teleost larvae in particular, is still largely unknown.

**Fig. 7:**
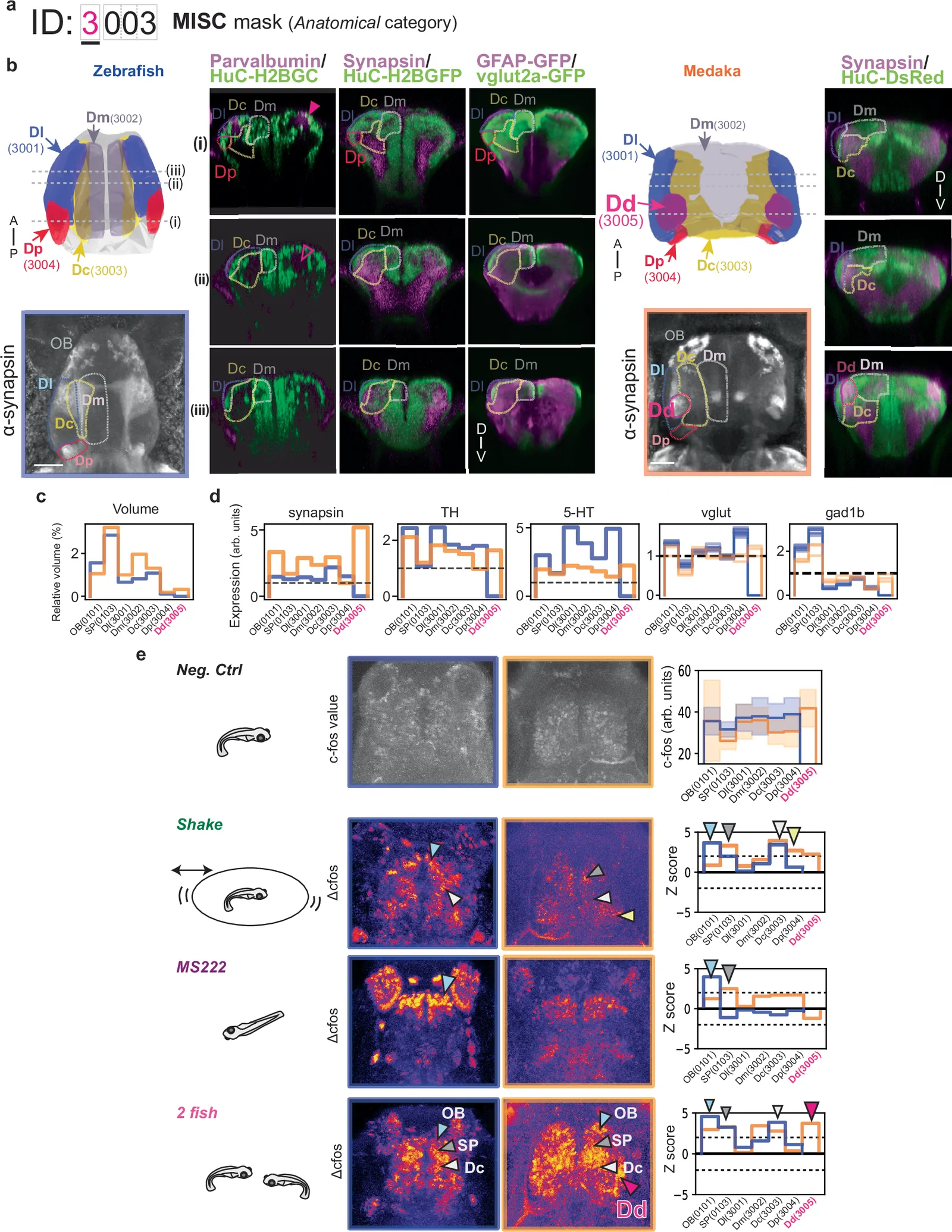
Comparative analysis of functional response properties in the telencephalon. **a** MISC masks IDs. The leftmost digit (#7) ‘3’ indicates MISC masks that are defined anatomically. **b** MISC masks of putative pallial subregions. Their boundaries are based primarily on topography and informed by immunostaining and transgenic marker expression. Maximal projections of the anti-synapsin immunostaining in the horizontal view are shown at the bottom left of the panel. Optical transverse sections of the telencephalon at the dotted line positions are shown on the right side. Positions of the sections are indicated by grey dotted lines in the left top 3 d visualisation. In zebrafish, we found anti-parvalbumin signals in Dl (indicated by a pink filled arrowhead) and Dc (indicated by a pink empty arrowhead) (See also Supplementary Fig. 7) (left column). Also, anti-synapsin signals (middle column) and GFAP-positive signals (right column) help us to define the subdivision of the pallium. HuC-H2BGC: Tg(HuC promoter-H2BGCaMP7f). Scale bar: 100 µm. Dl: the lateral pallium (ID: 3001), Dm: the medial pallium (ID: 3002), Dc: the central pallium (ID: 3003), Dp: the posterior pallium (ID: 3004), and Dd: the dorsal pallium (ID: 3005). D: dorsal, V: ventral. **c** Volumetric comparison of MISC masks. **d** Cross-species comparison of gene expression. Zebrafish (*vglut2a*): *n* = 8, zebrafish (*gad1b*): *n* = 10, medaka (*vglut2a*): *n* = 5, medaka (*gad1b*): *n *= 4. **e** *c-fos* expression analysis. Divergent neural activity by the treatments (shaking (second row), MS222 (third row) and 2 fish (fourth row) stimuli) across MISC masks in the telencephalon. Z-scores are plotted on the right. Blue line: zebrafish; orange line: medaka. Dotted lines indicate the 2 and −2 standard deviations. Divergent activity from the negative control are highlighted with coloured arrowheads. In the 2 fish stimuli, the olfactory bulb (OB, white arrowhead) and Dc (grey arrowhead) in zebrafish get activated, while in medaka Dm, Dc and Dd (magenta arrowhead) get activated. Source data are provided as a Source Data file.

To illustrate and characterise the diversity of the telencephalon in zebrafish and medaka larvae, we generated new MISC masks for the sub-compartments in the telencephalon and performed *c-fos* analysis across species (Fig. 7). The IDs of the new MISC masks defined in the telencephalon contain ‘3’ in the seventh digit (Fig. 7a). Because only a limited number of molecular markers are currently available to define pallial subdivisions, the boundaries of the putative Dm, Dc, and Dl regions are based primarily on topography and should be considered tentative. Labelling synapse density using a presynaptic marker, synapsin, which is ubiquitously expressed in all vertebrates, suggests different compartment patterns of computation in the telencephalon between the two species (Fig. 7b). We found that medaka displays stronger synapsin-positive signals in the dorsal area of the pallium (Dd, ID: 3005), which is consistent with the expression pattern in the adult medaka pallium^32^ (Supplementary Fig. 9). Also, strong synapsin signals was detected in Dc (ID: 3003) in zebrafish.

Furthermore, in zebrafish, we found that parvalbumin expresses in the cell bodies of the lateral area of the pallium (Dl) and axon fibres in the central part of the pallium (Dc), which is consistent in the anti-parvalbumin staining in adult zebrafish^33^. We also performed anti-PV staining in medaka larvae. Although strong PV immunoreactivity was detected in the cerebellum^34^ and skeletal muscles^35,36^, no PV-positive cells were observed in the pallium (Supplementary Fig. 7c). We also observed that expression patterns of other neurotransmitters are diverse across the MISC masks in the telencephalon; strong dopaminergic signals were detected in the lateral part of the pallium (Dl) in both species and Dd in medaka. Strong serotonergic signals were observed in Dl and the posterior part of the pallium (Dp) in zebrafish, whereas Dl and Dd in medaka; in both fishes, the pallial compartments show stronger glutamatergic signals than in the subpallium; in the end, the GABAergic signals are stronger in the olfactory bulb and the subpallium than in the pallial compartments in both species.

Finally, we observed *c-fos* expression in the telencephalon under different stimuli and compared neural activity across the MISC masks in the telencephalon in both species (Fig. 7b). We found that neural activity in negative control groups is similar across species and regions (Fig. 7c).

Shaking stimuli in medaka induced enhanced activity in many areas, such as the subpallium (grey arrowhead, ID: 0103), Dc (white arrowhead, ID: 3003) and Dp (yellow arrowhead, ID: 3004) (Fig. 7d). MS222 immersion led to strong activation in the zebrafish olfactory bulb (blue arrowhead, ID: 0101), whereas the subpallium (grey arrowhead, ID: 0103) was activated in medaka (Fig. 7e). For social stimuli, we found very strong signals in the telencephalon of both species. While zebrafish showed stronger activity in the olfactory bulb (blue arrowhead, ID: 0101), the subpallium (grey arrowhead, ID: 0103) and the Dc (white arrowhead, ID: 3003), the Dm and Dd (magenta arrowhead, ID: 3005) regions dominate in medaka, which suggests distinctly divergent processing of social stimuli across these two species.

## Discussion

In the current paper, we presented the newly developed cross-species comparative atlas platform and a general approach for analysing the brain regions of two evolutionarily diverged species of the same animal class, the zebrafish and the medaka. Using the platform, we show as proof of principle that several neural subclusters in the diencephalon and telencephalon exhibit volumetric and transcriptional differences between the species. Furthermore, we show that the MECE approach successfully enables the quantitative dissection of functionally distinct correlates.

In general, it is challenging to define, map, and compare homologous brain regions across species. In this paper, medaka MECE masks are defined based on literature and expert knowledge (Supplementary Table 1, 2, 3). There are two major approaches to consider and generate brain region MECE masks: one is based on anatomy and function (the columnar model), and the other is based on gene expression (the neuromeric model)^37–39^. Our MECE masks are originally based on the columnar model, though we tried to integrate transcriptomic information as much as possible. For example, in our atlas, the thalamic regions are categorised only into dorsal and ventral thalamus, whereas other atlases subdivide these regions differently. Amongst others, they define the “eminentia thalamus”, which includes and overlaps with our habenula and ventral thalamus. However, the raphe and clusters of the cranial nerves follow strictly a neuromeric logic. The readers who are more focused on development and transcription are recommended to check mapzebrain (https://mapzebrain.org/atlas/2d) as an alternative atlas resource, and in principle, MISC masks for the numeric model can be integrated into our atlas platform by reference brain registration. We believe that having two complementary approaches and a variety of different platforms that prioritise different aspects is useful for a diverse population of potential users.

The atlas-based platform can be extended by users by integrating their own dataset. This uploaded and integrated data can be analysed with the integrated visualisation and analysis tools. The platform, therefore, has the potential to become a community platform that acts as both a data and analysis portal, facilitating collaboration between researchers and accelerating the progress of knowledge. However, accurate registration of user acquired data to the reference brain is non-trivial and requires the careful execution of several steps. First, the image quality of the user data needs to be high with respect to signal to noise, contrast, and homogeneity of staining. Second, the uploaded data needs to match the reference brain with respect to size, developmental stage and orientation reasonably well. Third, the registration algorithm used to align user volumes to the reference brain needs to be selected carefully. Different programs exist, such as Computational Morphometry Toolkit (CMTK), Advanced Normalisation Tools (ANTS) and BigStream (https://github.com/JaneliaSciComp/bigstream), and many of them are still improving. Which particular one is being selected depends largely on the preference and expertise of the user. Here we offer a detailed recipe and tutorial for ANTS, which includes some benchmarking and quality cheques, but the choice of algorithm and software is ultimately up to the user. We acknowledge that this is the first release of our interactive cross-species platform, and we anticipate initial obstacles and problems with its smooth operation, where we will rely heavily on user feedback to improve, facilitate and optimise the performance.

Several larval zebrafish brain atlases have been published in the last decades. These include mapzebrain (https://mapzebrain.org/atlas/2d), ZebrafishBrain (https://zebrafishucl.org/zebrafishbrain)^19^, and the ZebrafishBrainBrowser (http://vis.arc.vt.edu/projects/zbb/) who have served the community well and were published by leading zebrafish research teams in Germany^40^, England^41^ and the US^42^. Our platform expands and extends these efforts by adding three critical components. First, it allows for quantitative and qualitative cross species comparison. Second, it allows for user-data to be uploaded, validated and integrated into the platform, which permits continuous scaling and expansion. Third, our ‘Manhattan Plot’ approach permits a systematic, rigorous and detailed comparison of anatomical features as well as transcriptional and functional activity across species and across defined brain regions. We believe these parallel efforts will benefit the fish community by storing and comparing the knowledge we acquire during the process of making and using the atlases.

MECE masks are not routinely updated by the community. Curation and updating of MECE regions require a substantial reorganisation of the database and will likely require the involvement of a ‘consortium’ of experts. This process should be accompanied by a conference proceeding or a publication. On the other hand, the process of updating MISC masks does not require much curation and quality control beyond publication in a peer-reviewed journal. Once this has happened, all newly published MISC masks are automatically transferred to the publicly accessible part of the FishExplorer. In the future, we will extend the atlas with older fish brains and/or new fish species, where we expect to segment new MECE brain regions that we could not find for larval zebrafish and medaka. MISC regions will be added continuously across species and developmental stages. Their incorporation into the platform is straightforward since they do not need to comply with any physiological or topological constraints. On the other hand, there is no guarantee that all parts of the brain or body develop at the same rate across species, and thus that there may be no such timepoint where everything is ‘matched’. This is an important caveat for this type of developmental comparative work in general. In order to clarify this, we could use the actual time point, such as 2 weeks after fertilisation, instead of an ambiguous term, such as “old juvenile”. The main challenge will be to guarantee appropriate quality control before formal incorporation of the Atlas. For example, it is difficult to keep all experimental variables exactly constant. Differences found between specimens should therefore be viewed more as qualitative suggestions rather than quantitative proof, and need to be followed up by explicit and quantitative testing in targeted experiments, which will be contingent on publication in peer reviewed journals.

FishExplorer allows the quantification of relative volumes, as well as gene expression profiles across the whole brain. The relative volumetric comparison across MECE regions allowed us to speculate on evolutionary mechanisms that might have led to brain specifications depending on the natural environments in which the species have evolved. We hypothesised that the more sensory modalities are used, the larger the brain regions corresponding to that modality have evolved to be, and vice versa. For example, some teleost species exhibit volumetric diversity in brain regions related to the sensory modality the species utilises^43^.

We used Manhattan plots to visualise the signal intensities in the image stacks, which is useful to extract the species difference. For example, the strong signals in the serotonergic mask in the caudal hindbrain in zebrafish (Fig. 5d, third row, ID: 4057) might suggest that serotonergic signalling in motor modulation may be more prevalent in zebrafish than in medaka. And the higher dopaminergic signal intensity in the pretectum in zebrafish than in medaka (Fig. 5d, fourth row, red arrowhead, ID: 4068) could reflect the modulation of visual signals under tighter dopaminergic control in zebrafish. Moreover, the stronger signals in noradrenergic caudal mask in the hindbrain in medaka (Fig. 5d, fourth row, green arrowhead, ID: 4069) suggest a possibly enhanced role of glial processing, which is known to be under the influence of noradrenergic inputs from nearby hindbrain regions^44^. This visualisation is very effective, however, users should be aware that it is very sensitive to staining bias, such as autofluorescence. It is essential to apply a well-validated normalisation strategy. In our neurotransmitter staining result, some of the samples were taken from previous reports, which made it harder to normalise the signals between species. In addition, some of the staining signals were detected in the different neurotransmitter MISC masks, since these masks are not mutually exclusive and allow for arbitrary overlap.

*c-fos* analysis showed that the overall expression was very similar between zebrafish and medaka in the negative controls, which is interesting given the large evolutionary distance between the two species. A few brain areas were more activated by external stimuli compared to the negative controls. The social stimuli revealed a conserved role of the telencephalon in both species; activation in the GABAergic cluster in the subpallium induces inhibition of broad areas across the whole brain, which aligns with previous studies^27,45^. The vibration assay showed a strong activation in the hindbrain glutamatergic neurons in zebrafish, which was consistent with previously reported activation of the octaval nerve by the zebrafish auditory system^46^. With MS222 immersion stimuli, we found that the olfactory epithelium and olfactory bulb are activated, which is consistent with previous findings that this area is activated by chemical sensation^19,26,47^. Also, the activation in the neuromast indicates that neuromasts are sensitive to these chemical substances^48^. In medaka, we found activation in a broader area of the brain, which might indicate a different chemical sensation than in zebrafish. For example, one possible explanation about the different results in MS222 exposure is that MS222 might be less effective in medaka, or, alternatively, that the optimal concentration of MS222 for medaka is different from that for zebrafish. Since comparing anaesthetic levels across species are difficult, we need to be careful when adjusting the amount of MS222 when using it as an anaesthetic.

Our interactive brain atlas platform provides tools for users to generate new MISC region masks, allowing unrestricted specification of any particular region of interest. Here, we have created masks for the telencephalon, which has received particular attention in gene profiling and functional analysis. A recent paper showed that there is an epigenetically distinct region in the dorsal pallium (Dd) of medaka larvae^32^, which had not been confirmed to exist also in zebrafish larvae, although juvenile and adult zebrafish have distinct compartments in the dorsal pallium based on the function and connectivity^33,49,50^. PV has been used as a marker of Dl and Dc in adult zebrafish, and we detected PV expression in the corresponding pallial regions of larval zebrafish. In contrast, PV expression was not detected in the larval medaka pallium. This finding highlights substantial interspecies differences in gene expression and cellular composition within the teleost pallium. In fact, multiple other teleost species exhibit a distinct anatomical compartment in the dorsal area in the pallium^51–53^. Therefore, from a comparative and evolutionary perspective, a Dd-like domain may also be present in zebrafish, including at larval stages, although this will require further characterisation. Therefore, the putative Dm, Dc, and Dl masks presented in this study are based primarily on topographical features and should be regarded as tentative anatomical assignments. The current pallial masks represent tentative anatomical assignments based on the best available evidence and may be refined as additional molecular, developmental, and connectivity data become available. One advantage of the MISC framework is that it is designed to accommodate such refinements while preserving the quantitative analyses based on the atlas.

This significant morphological change most likely be due to post-hatch neurogenesis in the telencephalon^54^. Here, we compared the gene expression and neural activity in different contexts in the telencephalic compartment of larval medaka and zebrafish, and we found that the medaka Dd region has a unique expression pattern of marker genes. Furthermore, *c-fos* analysis of regions within the telencephalon suggested that the Dd regions in medaka are differentially activated compared to other pallial regions, suggesting a possible functional significance of this region. Cross-species analysis of more complex behaviours will require a detailed description of the adult and later developmental stages in both species, necessitating an extension of our atlas platform to developmental stages at older ages.

The study of evolutionary diversity and principles of behavioural mechanisms using comparative approaches in neuroscience and neuroethology has recently received increased attention with the advances in imaging technologies, genetics and genomics. Our interactive multi-species community atlas has the capability to accommodate, with moderate effort, datasets collected by research groups studying zebrafish and medaka neuroscience around the world. Currently, the core and integral service we offer relates to visualisation and depository services for zebrafish and medaka brains, but we also share the recipes and protocols for registration to reference brains, and mask generation for other species and developmental stages (see ‘Cross-species analysis’ tutorial in the FishExplorer). At the next step, we plan to extend our platform to other teleost fish, such as Danionella and killifish, and to add further developmental stages till the adult stage to facilitate the study of developmental aspects in addition to interspecies comparisons.

Finally, we plan to provide and implement a direct bridge from the FishExplorer to whole brain annotated EM volumes that are expected to be published in the near future, thereby allowing the integration of connectomic circuit analysis with the transcriptional and functional information provided by our platform.

## Methods

### Animal experiments and ethics

All experiments followed institution IACUC protocols as determined by the Harvard University Faculty of Arts and Sciences standing committee on the use of animals in research and teaching. The animal experimentation protocols, 25-03 and 22-04 were submitted and approved by this institution’s animal care and use committee (IACUC). This institution has Animal Welfare Assurances on file with the Office for Laboratory Animal Welfare (OLAW), D16-00358 (A3593-01).

We followed the ARRIVE guideline 2.0. Sample size is described in the Figure legends and method section. Inclusion and exclusion criteria: when the registered image stacks did not align well with the reference brain, we did not include those stacks for further analysis. Randomisation: for the analysis of *c-fos* staining experiments, fish samples were imaged in random order with the same imaging parameters. Blinding/masking, outcome measures: For quantitative analysis of the gene expression, we masked the label of genes until the result interpretation.

### Fish maintenance

All medaka fish and zebrafish transgenic lines used in this research were raised in methylene blue water for a few days and then in filtered fish facility water on a 14 h light, 10 h dark cycle at 28 °C. We kept the density of embryos around 30 fish per petri dish. Under 28 °C incubation, medaka hatch at 6-7 days post-fertilisation (dpf), while zebrafish hatch at 2 dpf^55^. We used 0- or 1-day post-hatch larvae for medaka and 5–7 dpf larvae for zebrafish in the experiments.

### Transgenic lines

The medaka Tg(vglut:GFP) line was generated in the Higashijima lab and provided by NIBB. The medaka Tg(Ath5:GFP) line was generated by the Wittbrodt lab^56^. The medaka Tg(HuC:loxp-DsRed-loxp-GFP) in the ST3 mutant background was generated by crossing medaka Tg(HuC:loxp-DsRed-loxp-GFP)^57^ with ST3 mutants^58^. The medaka Tg (ST3-eyesBlack) was established by crossing ST3 mutants and wildtype (drR), then embryos were screened for the absence of autofluorescence around the head, but with pigment cells in the eyes. The zebrafish *nacre* mutant^19^ was used for immunostaining and in situ hybridisation.

### Immunochemistry and in situ hybridisation

We followed the immunostaining procedure from the previous study^19^. Information on the primary antibodies can be found in Supplementary Table 4. Secondary antibodies conjugated to Alexa fluorophores (Life Technologies) were diluted 1:250. Hoechst dye was diluted 1:1000.

In situ hybridisation experiments were performed according to previously published procedures^59,60^. In brief, after fixing fish larvae in 4% PFA/PBS overnight, the larvae were dehydrated with 100% methanol. Then we washed with ethanol and performed permeabilisation with ethanol/xylol solution. Then we rehydrated with a sequence of ethanol/H_2_O with 0.1% Tween-20. Then we permeabilized larvae with 80% acetone/H_2_O at − 20C for 30 min and washed larvae with PBST (0.1% Tween-20, PBS). We pre-hybridised the larvae with a prewarmed hybridisation buffer for 4 hours and incubated them with HCR probe for 60 hours at 37 C. Then, the larvae were washed with a 5 x SCCT buffer and incubated with an amplification buffer for 30 min. After that, larvae were mixed with hairpin that heated up at 95 C for 90 seconds, and incubated overnight at RT. After washing with 5 x SCCT, we cleared the larvae with a series of glycerol and stored at − 20C. We always stained with *snap25a*, which was used as a whole-neuron marker for the registration. We ordered HCR-RNA bundles from Molecular Instruments, Inc.

### Backfill labelling

We first embedded larvae into 2% agarose gel sideways down. After scooping out the gel above the fish, Texas Red crystals were injected into the floor plate of larvae with a tungsten needle. We removed the larvae from the gels immediately after injection and placed them in the fish water. The next day, we fixed the larvae in 4% PFA with Hoechst (1:1000) overnight. After washing with PBS-Tx, we embedded the fish larvae in agarose (2% in PBS) and performed imaging under a confocal microscope (Zeiss LSM780). We identified the Mauthner cells by their shape and other structures, such as the nucleus of the medial longitudinal fasciculus (NucMLF), RoM cells and MiV cells by their position relative to the Mauthner cells located in rhombomere 4^61^.

### FM1-34 staining

We placed the fish larvae in FM1-34 (3 µM) solution for 30 sec. Then the fish were transferred to fish water for 10 min and washed several times. We fixed the larvae overnight in 4% PFA with Hoechst dye (1:1000). After washing with PBS-Tx, we embedded the fish larvae in agarose (2% in PBS) and performed imaging under a confocal microscope (Zeiss LSM780).

### Confocal imaging

Samples whose subpopulation are labelled were imaged with confocal microscopy (Zeiss LSM780). We used a 10x objective, and the spatial resolution of the image stacks was 0.59 µm x 0.59 µm x 2 µm or 0.59 µm x 0.59 µm x 1.5 µm.

### X-ray imaging

Medaka (8dpf) and zebrafish (7dpf) samples were stained to enable both electron microscopy and X-ray imaging following a protocol modified from a previous study^58^. Unless noted, all steps were performed at room temperature (RT). One day after hatching the larva was anaesthetised and embedded in agarose. We replaced the water by a dissection solution (64 mM NaCl, 2.9 mM KCl, 10 mM HEPES, 10 mM glucose, 164 mM sucrose, 1.2 mM MgCl2, 2.1 mM CaCl2, pH 7.5) supplemented with 0.02% tricaine^62^. We then cut small slits in the agarose to expose the eyes, performed bi-lateral enucleations and tail resection to enhance ultrastructural preservation and heavy metal staining. We used a custom-made hook that was carefully inserted behind the eyes to prevent brain damage. The larva was immediately transferred to a fixation solution at 4 °C (2.5% glutaraldehyde, 0.1 M cacodylate buffer supplemented with 4.0% mannitol at pH7.4; cacodylate buffer: 0.3 M sodium cacodylate, 6 mM CaCl2, pH7.4). To enhance the fixation, the tissue was rapidly microwaved (cat. no. 36700, Ted Pella, equipped with power controller, steady-temperature water recirculator and cold spot) in the fixative solution (this step lasted < 5 min after initial transfer into fixative). The microwaving sequence was performed as in a previous study^63^: at power level 1 (100 W) for 1 min on, 1 min off, 1 min on; then set to power level 3 (300 W) and fixed for 20 s on, 20 s off, 20 s on, three times in succession. Fixation was then continued overnight at 4 °C in the same solution. The following day, the sample was washed again in 0.5x cacodylate buffer (3 exchanges, 30 min each before being osmotized (2% OsO4 in 0.5x cacodylate buffer, 60 min). After a quick wash (< 1 min) in 0.5x cacodylate buffer, the sample was reduced in 2.5% potassium ferrocyanide in 0.5x cacodylate buffer for 60 min, then washed with filtered H2O (3 exchanges, 30 min each) and then incubated with 1% (w/v) thiocarbohydrazide (TCH) in filtered H2O (and filtered with a 0.22um syringe filter before use) for 20 min to enhance staining^64^. As TCH dissolves poorly in water, the solution was heated at 60 °C for ~ 90 min with occasional shaking before filtering and then placed at RT for 5 min before the incubation step. The sample was then washed with filtered H2O (3 exchanges, 30 min each) before the second osmication (2% OsO4 in filtered H2O, 60 min) and then washed again (3 exchanges, 30 min each). Then, en-bloc staining was performed overnight using 1% uranyl acetate in filtered water at 4 °C. The solution was sonicated for 90 min and filtered with a 0.22 μm syringe filter before use. Steps involving uranyl acetate were performed in the dark. The following day, the samples were washed with filtered H2O (3 exchanges, 30 min each). Next, the samples were dehydrated in serial dilutions of ethanol (25%, 50%, 75%, 90%, 100%, 100% for 10 min each step), then in propylene oxide (PO) (100%, 100%, 30 min each step). Infiltration was performed using LX112 epoxy resin with BDMA (21212, Ladd) in serial PO dilutions steps, each lasting 4 h (25% resin/75% PO, 50% resin/50% PO, 75% resin/25% PO, 100% resin, 100% resin). Samples were mounted in fresh resin in a mouse brain support tissue^63^ with the head exposed to facilitate cutting by avoiding the sample to sink to the bottom of the resin molds. Mouse tissue was fixed using standard procedures^65^ then cut into 2-3 mm wide cubes, which were pierced using a puncher (0.75 mm, 57395, EMS) to insert the larva. The cubes were stained along with fish samples using the protocol described above, except that the uranyl acetate overnight step was performed at RT. The samples were then cured with support tissue during 3 days at 60 °C. For all steps, a rotator was used. Aqueous solutions were prepared with water passed through a purification system (Arium 611 VF, Sartorius Stedim Biotech). The protocol lasted 5 consecutive days, including surgery, fixation, staining, and resin embedding, followed by 3 days of resin curing. The block was finally trimmed to expose the head of the fish for X-ray imaging (Xradia 520 Versa, ZEISS). Imaging was performed at 40 kV, 76 uA, 20X optical magnification, 0.35 µm isometric voxel resolution and lasted 96 hours (100 s per image).

To register the brain masks using the X-ray imaging dataset, we performed the annotation using the Fiji plug-in BigWarp^66^. Since the X-ray imaging dataset covers larger areas of the fish, such as gills and other organs, we registered the masks from the reference brain onto the X-ray data.

### Brain registration

After the imaging step, all imaging volumes were registered to the reference brain using the Advanced Normalisation Tools (ANTs)^21^. We performed the registration with rigid [-m GC, -c 300 x 300 x 300 × 0, 1e-8,10, -f 12 x 8 x 4 × 2, -s 4 x 3 x 2 × 1], affine [-m GC, -c 300 x 300 x 300 × 0, 1e-8,10, -f 12 x 8 x 4 × 2, -s 4 x 3 x 2 × 1] and SyN (diffeomorphic symmetric) [-m CC, -c 300 x 300 x 300 × 0, 1e-8,10, -f 12 x 8 x 4 x 2 x 1, -s 4 x 3 x 2 x 1 x 0] transformations, with the specified parameters. The registrations were carried out on a high-performance cluster with 300 GB memory and 150 CPU cores.

The tutorial with a detailed description of the parameters and step-by-step instructions for the registration with the Python script will be available on the FishExplorer website (https://fishexplorer.zib.de/tutorial/image-registration.html).

For the immunostained samples, we always stained with Hoechst dye and used the Hoechst reference brain stacks for registration. To register HCR-stained samples with the reference brain, we imaged a few stacks of *snap25a-* and Hoechst-stained brains and made a *snap25a* channel as the reference brain. HCR-stained samples always had *snap25a* staining to register other staining channels to the reference brain. After registration, we created the average stacks that were used to determine the region masks.

### Segmentation of brain regions with morphological inter-slice interpolation and deep learning

Manual segmentation of brain regions requires a lot of time and expert effort. Our aim here is to reduce the effort of manual segmentation by using deep learning models and morphological inter-slice interpolation. To this end, we used the following dataset and methodology.

We recruited the Hoechst dye-stained medaka reference brain to perform the segmentation experiments. The original size of the reference stack was 1401 x 2045 x 278 voxels. We upsampled the stacks to 2045 x 2045 x 278 voxels to obtain an isotropic voxel size. For performing our experiment, we downsampled the stack to 512 x 512 x 278 voxels to increase the receptive field of the network. We applied the segmentation to 21 large brain regions. To test our method, the expert manually performed complete labelling of 8 regions (Supplementary Fig. 3) using our Fiji plug-in, NeMO. The remaining 13 regions were initially left unlabelled and were later labelled based on experimental results of these 8 regions.

First, we tested how much manual labelling was sufficient to train the network model. From the 8 fully labelled binary-segmented brain regions, we generated training data with 20%, 25%, 30% and 35% of them using the deep learning panel of the Fiji plug-in, NeMO. We did not keep any data for validation, as this would have required an additional 10–15% of segmented data. The major drawback of not using validation data would be overfitting the model, but our goal is to reduce manual effort, not to prevent overfitting. To increase the amount of training data, we applied data augmentation. Specifically, we applied random shifts with a range of [− 50, 50] pixels, rotation of [− 5, 5] degrees, random flips, shear of 0.06 degrees in counterclockwise direction, zoom with a range of [0.94, 1.06], i.e., 94% zoom-in and 106% zoom-out, respectively. We generated 10,000 training images and 500 test images for each subset using data augmentation. The test images were augmented in order to have more images to test the network model. Note that both the images and the annotations were augmented in the same way.

We trained a 2D-UNet network^22^ on the augmented training data using an ADAM optimiser with a learning rate of 0.0001. We used binary cross-entropy as a loss function. The hyperparameter batch size was set to 2, and the step per epoch was set to 100. We trained the network for 50 epochs and assessed the quality of the models using the dice score. Supplementary Fig. 3**a** compares the dice score across all the subsets. We selected the model with the lowest percentage of splits and a dice score > 80%. 20% of manual labelling was sufficient to predict the complete label fields for the forebrain, midbrain, retina, cerebellum and periventricular stratum, whereas telencephalon and neuropil required 25% and hindbrain 30%, respectively. Based on these results, the remaining 13 regions were then partially segmented by experts up to 20% and experiments were performed on these partially segmented regions to obtain the full label fields (Supplementary Fig. 4).

Next, we incorporated the morphological inter-slice interpolation method^63^ into the NeMO plug-in and performed a comparison with the deep learning method. Supplementary Table 5 provides a quantitative comparison of the results obtained with the 2D U-Net and the morphological inter-slice interpolation method applied across several brain regions. We used intersection over union (IOU) as an evaluation metric. The results of both methods are very similar. Both methods work well when there is a single connected component in the image and produce more errors when the number of connected components changes from one to many. Although the morphological inter-slice interpolation method is computationally less expensive as compared to 2D U-net, it always expects a labelled stack as input. On the other hand, the deep learning method can be used to predict regions in new datasets without the need for labelled slices.

### Segmentation of brain region masks with Fiji plug-in, Neuro Mask Organiser (NeMO)

For the segmentation of the brain regions in medaka, we have implemented a tool, Neuro Mask Organiser (NeMO), which can be integrated as a plug-in in the ImageJ/Fiji platform^67^. The plug-in is available on GitHub (https://github.com/zibneuro/fishexplorer/tree/master/NeMO). The main motivation for using this custom plug-in instead of state-of-the-art segmentation editors, such as those available in Amira^68^ or VAST^69^, is that we wanted to provide in a simple open-source tool exactly the few specific functions needed to solve the task. These include copying masks to the left/right brain hemisphere, comparing masks with registered zebrafish masks, and finding overlaps between regions, to enable fast and interactive ground-truth labelling. The new masks/regions of interest (ROIs) are labelled using the polygon and free hand selection tools of ImageJ. The plug-in (Supplementary Fig. 2) consists of several panels: (1) The region panel displays a list of brain regions (indicated by red arrowhead). Users can select/deselect a brain region by clicking on an item in the list. The panel also displays the name of the currently selected region and the region of interest in a slice. (2) The colour channel panel (indicated by a yellow arrowhead). To label a selected region, users often need the boundaries of adjacent regions. This is accomplished by loading adjacent regions as a coloured channel. (3) Other panels (indicated by a purple arrowhead). This allows users to open two important sub-panels: the overlap panel, which provides a list of overlaps between the selected region and the loaded regions in the colour channel panel. It also provides helper functions to fix the overlaps and a deep-learning panel that allows users to create the training dataset and fuse the model predictions. Users can choose different options for creating a training dataset, e.g., training on whole images or image sections, percentage of labelled data, split strategy, etc. (4) ROI functions (indicated by a green arrowhead). They enable users to perform Boolean operations between a selected ROI and the currently labelled ROI. It also enables users to copy a selected ROI to the other hemisphere or to the next/previous slice. (5) The ROI panel (indicated by a blue arrowhead). It consists of two sublists, namely labelled ROIs for a selected region in the current slice and predicted ROIs in the current slice.

We defined MECE masks and MISC masks based on cell localisation by Hoechst staining and gene expression visualised by immunostaining, in situ hybridisation and transgenic lines. Please see Supplementary Table 1, 2, 3 for more details.

### IDs of MECE and MISC masks

All zebrafish MECE masks and most of the MISC masks are available in the Z-brain atlas (https://fishexplorer.zib.de/downloads). The masks for serotonergic and oxytocinergic MISC masks of zebrafish were generated in the same way as the medaka MISC masks by using the Fiji plug-in NeMO.

We’ve implemented a comprehensive ID system for mask identification: each ID consists of 10 digits, accommodating up to 10 billion unique identifiers, which should be sufficient even if we include more species and developmental stages. We reserve the two most significant (leftmost) digits, #1 and #2, to identify up to 100 different species. Here we assign “01” for zebrafish and “02” for medaka. If future development requires the inclusion of more than a hundred species (currently ~ 30,000 different teleost species have been described), the ID can be extended to 11, 12 or even 13 digits. Digits #3 and #4 in our current convention are reserved to describe the developmental stage. We anticipate that teleost development can be well captured within 100 distinct stages. We have chosen to define the first 21 stages in close analogy to the ‘days post fertilisation’ convention currently used in the zebrafish field. Since our atlas currently describes only one developmental stage in zebrafish (6 dpf) and medaka (1 day ‘post hatch’, which corresponds to 6 dpf in zebrafish), all of our IDs have “06” in the third and fourth positions. Digits #5 and #6 are reserved for describing different tissue types, such as “00” for the nervous system, “01” for epithelial tissue, “02” for vasculature, and so on, allowing any and all body tissue to be incorporated.

The last 4 digits are reserved for all of our region masks (MECE and MISC). The first digit (#7) codes for MECE masks, taking values between 0 and 2, where “0” indicates the CNS, “1” indicates PNS and “2” is still unassigned. Digit #8 codes for the MECE ‘macro’ mask, allowing for up to 10 macro regions, with only “1” - “5” currently assigned. The remaining two digits, #9 and #10, are reserved for ‘mini masks’, allowing up to 100 ‘mini’ masks to be assigned to each ‘macro’ region. Values of digit #7 between “3” and “9” are reserved for all MISC masks, with “3” loosely reserved for anatomically defined MISC masks, values “4”, “5” and “6” for transcriptionally defined MISC masks, and values “7”, “8” and “9” reserved for functionally identified MISC masks. Together, this allows for a total of up to 7000 MISC masks to be incorporated. For comparison, our current version contains ~ 200 MISC masks for zebrafish and ~80 MISC masks for medaka, numbers we expect to grow rapidly as the community engages and uses the platform.

### Quantification of signal intensity and size of brain region

Quantitative comparison of gene expression between species allows us to assess differences in the function of neural circuits and brain regions. One difficulty in comparing signal intensity was normalising and comparing the signals across regions located at different depths and between different species. Since slices close to the microscope objective tend to have higher signals than those farther away, we first pre-processed the image stacks to obtain the signals in the nuclei of interest. The zebrafish stacks were “ANDed” with the “Elavl3-H2BRFP” stacks, whereas medaka stacks were “ANDed” with the “Hoechst” stacks. Next, to normalise the signals across different planes, we first applied the (histogram-based matching technique) Fiji “bleach correction” plugin^70,71^ to correct for exponential dimming with increasing imaging depth. The average value of gene signals in each MECE mask was then divided by the average value of all the MECE masks. To quantify the size of the MECE and MISC masks, numbers of pixels inside the masks were divided by the total number of pixels within all the MECE macro masks (masks of “Eyes” are excluded).

The 5HT, TH, and Oxt datasets were obtained from the Z-Brain atlas^19^. These datasets consist of averaged image stacks, and the original individual imaging datasets are not publicly available. Therefore, biological variability could not be estimated, and error bars are shown only for analyses based on original datasets (e.g., glutamate and GABA).

### Statistics and reproducibility

No statistical method was used to predetermine sample size. Sample sizes were chosen based on the availability of technically successful biological replicates and the reproducibility of staining and imaging across individuals. The study was designed primarily to establish and demonstrate a cross-species atlas platform rather than to test biological differences in gene expression, neurotransmitter expression, or *c-fos* activity; these analyses are therefore presented descriptively and were not subjected to inferential statistical testing.

Pan-neuronal or nuclear reference staining was included to assess staining and imaging quality and to enable anatomical registration and signal normalisation. Samples in which the corresponding pan-neuronal or nuclear reference signal was insufficient for reliable registration or normalisation were excluded from quantitative analysis. Exclusion was based on the quality of the reference signal rather than on the experimental signal of interest or the resulting quantitative measurements.

For the *c-fos* experiments, samples were randomised across experimental conditions, and sample identities were blinded during staining and imaging. Experimental conditions were revealed after image acquisition and before quantitative analysis. Other experiments were not randomised or blinded. Experimental staining and imaging were performed across independent biological samples, and the reported anatomical, gene-expression, neurotransmitter-expression and activity patterns were reproducibly observed across successfully stained and imaged samples.

The 5HT, TH and Oxt datasets were obtained as averaged image stacks from the previously published Z-Brain atlas. The original individual imaging datasets are not publicly available; therefore, biological variability and sample-level reproducibility could not be independently assessed for these datasets in the present study.

For volumetric comparisons of corresponding brain regions between zebrafish and medaka, relative volume was calculated as the number of pixels within each MECE or MISC mask divided by the total number of pixels contained within all MECE macro masks, excluding the eyes. Differences in relative volume between species were assessed using exact two-sided Mann–Whitney *U* tests (zebrafish, *n* = 7; medaka, *n* = 3). Degrees of freedom are not applicable to the Mann–Whitney *U* test. Statistical comparisons were performed only for brain regions representing > 1% of the total brain volume in at least one species. *P-*values were adjusted for multiple comparisons using the Benjamini–Hochberg false discovery rate (FDR) procedure, with statistical significance defined as FDR-adjusted *P* < 0.05. Effect sizes were quantified using rank-biserial correlation (*r*rb), with 95% confidence intervals calculated using a normal approximation via Fisher transformation. Given the limited sample size, we additionally required a fold change > 1.5 or < 0.67 as a criterion for highlighting differences of substantial magnitude. Fold change was calculated as the mean relative volume in medaka divided by that in zebrafish. Exact *P-*values, FDR-adjusted *P-*values, *U* statistics, effect sizes, 95% confidence intervals and individual measurements are provided in the Source Data file.

### c-fos experiments and analysis

For the social stimuli assay, two fish larvae were placed in a petri dish at room temperature with light on. For the MS222 assay, 100 µg/ml of MS222 (Sigma-Aldrich) was dissolved in filtered fish water buffered to pH 7-8. W Samples were collected 40 min after treatment and immediately fixed with 4% PFA/PBS overnight. For the vibration assay, the dish containing fish larvae was placed on to the shaker (SCILOGEX, SCI-0180-S) at the speed of 60 rpm for 1 hour. We note that 30 minutes of stimulation might yield a slightly better signal to noise ratio since the time constant of *c-fos* is on the order of 30 minutes.

Then HCR experiments were then performed with *c-fos* and *snap25a* probes as described above. *snap25a* was used to normalise and quantify the *c-fos* signal as it is widely expressed in the nervous system.

To analyse the neural activity across samples in each treatment, we first created a *snap25a* channel for the reference brain. After registering the image stacks to the reference brain by aligning to this *snap25a* channel, we calculated the average value of *c-fos* and *snap25a* staining across all MECE and MISC masks. For each brain region, we calculated the normalised *c-fos* expression as the ratio of *c-fos* signal intensity to *snap25a* signal intensity to account for regional differences in neuronal density. To enable cross-region comparisons, we computed z-scores for each region by standardising the *c-fos/snap25a* ratios across all brain regions within each individual.

The image stacks were generated by averaging each pixel across samples and then displaying the maximum projected image. The lookup table was chosen so that the highest and lowest values matched the maximum and minimum of the lookup table. For the *c-fos* analysis in the telencephalon, the same analysis was performed using the MISC masks created for the telencephalon.

### Reporting summary

Further information on research design is available in the Nature Portfolio Reporting Summary linked to this article.

## Supplementary information

Supplementary Information

Reporting Summary

Transparent Peer Review file

## Source data

Source Data

## Data Availability

The FishExplorer platform provides access to medaka reference brain stacks and atlas imaging data, zebrafish reference and atlas imaging data, MECE and MISC masks, gene-expression imaging datasets, and neurotransmitter imaging datasets. The *c-fos* image data are available through the international open-access repository Dryad (DOI: 10.5061/dryad.zw3r228hw). FishExplorer also provides a tutorial describing the image-registration parameters and workflow, together with the Python script required for registration. Source data are provided in this paper.

## Code availability

The Fiji-based plug-in Neuro Mask Organiser (NeMO) is available on GitHub at https://github.com/zibneuro/fishexplorer/tree/master/NeMO.
