# Supplementary material for: A cross-species atlas platform for comparative neuroscience in teleost fish
Source: Nat Commun. 2026 Sep 25;17:11114. doi: 10.1038/s41467-026-77879-1 (PMC13696724; doi:10.1038/s41467-026-77879-1)
Supplement: Supplementary file 1 [file 41467_2026_77879_MOESM1_ESM.pdf]

A cross-species atlas platform for comparative neuroscience in teleost fish

Sumit Kumar Vohra[1,2], Tinatini Tavhelidse-Suck[3], Simon Knoblich[4], Ali Seleit[4], Jonathan Boulanger-Weill[5,6], Sydney Chambule[7], Ariel Aspiras[7], Cristina Santoriello[7], Owen Randlett[8], Joachim Wittbrodt[3], Jeff W. Lichtman[5], Mark Fishman[7], Hans-Christian Hege[1], Daniel Baum[1], Florian Engert[5], Yasuko Isoe[5*]

Affiliations

1. Visual and Data-Centric Computing, Zuse Institute Berlin, Takustraße 7, 14195 Berlin, Germany

2. Research group behavioral neuroscience, Leibniz-Institut für Neurobiologie (LIN), Brenneckestraße 6, 39118 Magdeburg, Germany

3. Centre for Organismal Studies (COS) Heidelberg, Heidelberg University, Im Neuenheimer Feld 230, 69120 Heidelberg, Germany

4. Developmental Biology Unit, European Molecular Biology Laboratory (EMBL) Heidelberg, Meyerhofstraße 1, 69117 Heidelberg, Germany

5. Department of Molecular and Cellular Biology, Faculty of Arts and Sciences, Harvard University, Cambridge, Massachusetts, 02138, USA

6. Sorbonne Université, INSERM, CNRS, Institut de la Vision, F-75012 Paris, France

7. Department of Stem Cell and Regenerative Biology, Faculty of Arts and Sciences, Harvard University, Cambridge, Massachusetts, 02138, USA

8. Laboratoire MeLiS, Université Claude Bernard Lyon 1 - CNRS UMR5284 - Inserm U1314, Institut NeuroMyoGène, Faculté de Médecine et de Pharmacie, 8 Avenue Rockefeller, 69008 Lyon, France

*Correspondence: Yasuko Isoe (yasuko_isoe@fas.harvard.edu)

Supplementary Information

Supplementary Figures 1 - 9

Supplementary Tables 1 - 5

Supplementary Figures

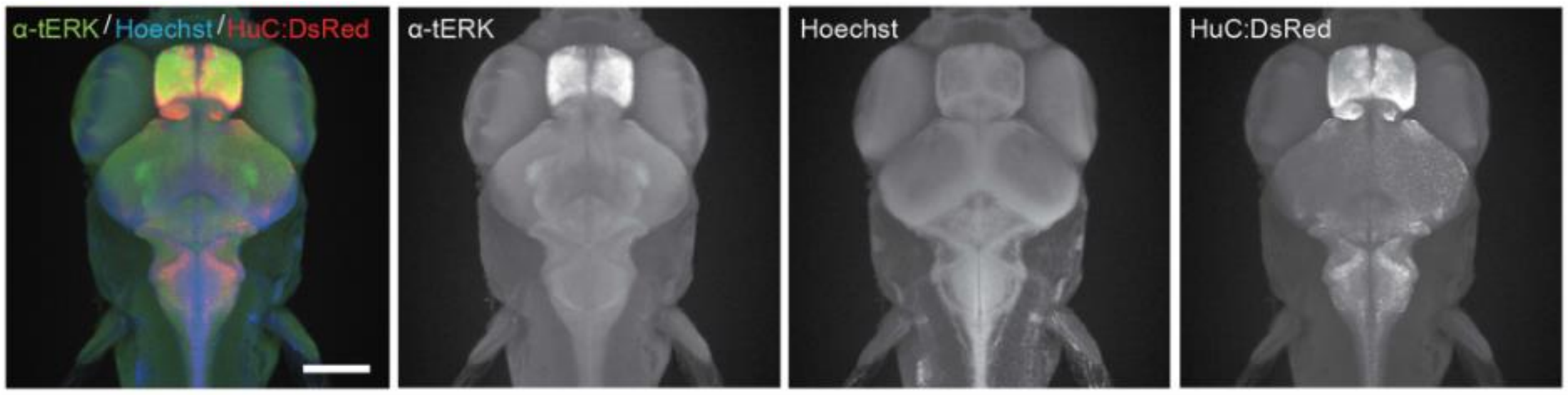

Supplementary Figure 1. Medaka reference brain

Reference brain stacks. We have three channels in the medaka reference brain: (1) tERK channel: total extracellular signal-regulated kinase (tERK) is stained in all cell bodies, which is also used for the Z-brain reference and is used to register neural activity with phospho-ERK (pERK)/tERK staining [19]; (2) Hoechst channel: All nuclei are labelled by Hoechst staining [71]; (3) HuC promoter channel: The HuC (*elav*) promoter is driven pan-neuronally and is commonly used for GCaMP-based calcium imaging to analyse *in vivo* neural activity [6]. The larvae of Tg (HuC:DsRed, red) line were stained with anti-tERK immunohistochemistry (green) and Hoechst dye (blue). Scale bar: 125µm. Maximum projections of the imaging stacks are shown.

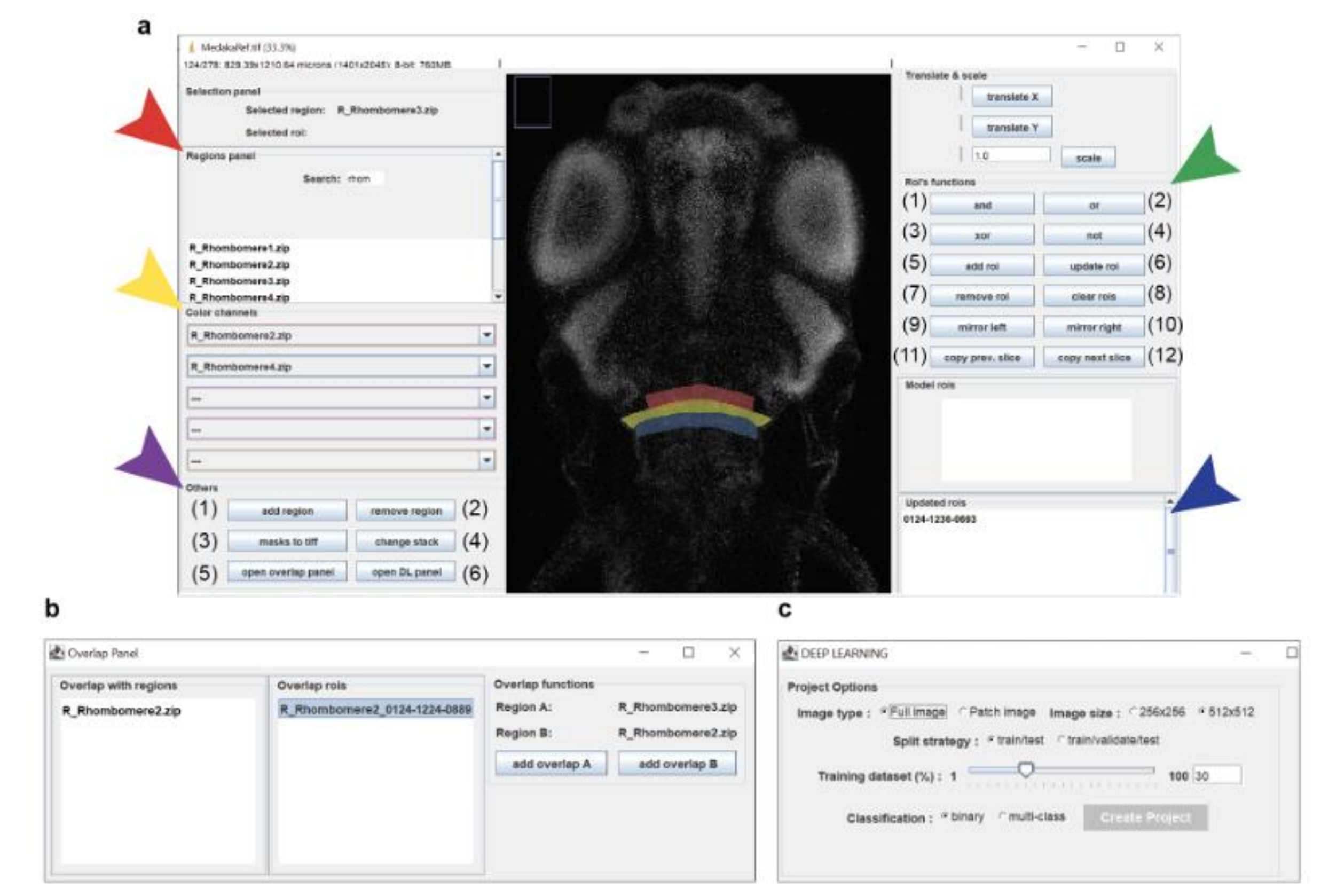

Supplementary Figure 2. Neuro Mask Organizer (NeMO) - a new interactive Fiji tool for editing masks

(**a**) The Fiji plug-in has several utility functions, including comparing masks with registered regions and fixing overlaps between regions. By visualising many adjacent region masks, new masks can be drawn and added. The red arrowhead shows the list of the masks to be modified, the yellow arrowhead shows the list of masks in different colours. The purple arrowhead shows the function buttons (1) “add region”, (2) “remove region”, (3) “masks to tiff”, (4) “change stack”, (5) “open overlap panel”, and (6) “open DL panel”. The green arrowhead indicates the functions (1) “and”, (2) “or”, (3) “xor”, (4) “not”, (5) “add roi”, (6) “update roi”, (7) “remove roi”, (8) “clear rois”, (9) “mirror left”, (10) “mirror right”, (11) “copy prev. Slice” and (12) “copy next slice”. And the blue arrowhead indicates regions of interest (ROIs), which are displayed in yellow.
(**b**) The overlap panel provides utility functions to fix overlaps between regions.
(**c**) The deep learning (DL) panel allows the user to create a training dataset based on different parameters, including classification strategy (binary/multi-class), split strategy (train/test vs. train/validate/test), and training on whole images or patches. The parameters chosen and the rationale behind are described in the **Section Materials and Methods**.

a

Regions	Thickness	20%		25%		30%		35%	
		Test loss	Test dice score	Test loss	Test dice score	Test loss	Test dice score	Test loss	Test dice score
Forebrain	219	0.0217	0.9052	0.0215	0.9016	0.0141	0.9221	0.0218	0.9051
Midbrain	179	0.0096	0.9523	0.0161	0.9486	0.0099	0.9452	0.0145	0.9388
Hindbrain	201	0.0341	0.6970	0.0351	0.6924	0.0108	0.9395	0.0143	0.9371
Retina	181	0.0149	0.9428	0.0173	0.9297	0.0164	0.9382	0.0178	0.9270
Cerbellum	182	0.0046	0.8928	0.0039	0.8995	0.0035	0.8818	0.0044	0.8858
Telencephalon	157	0.0136	0.6545	0.0142	0.8595	0.0059	0.8835	0.0076	0.8871
Stratum Periventriculare	176	0.0143	0.9320	0.0279	0.9079	0.0150	0.9250	0.0172	0.9251
Neuropil	131	0.0181	0.6618	0.0090	0.8791	0.0055	0.8951	0.0063	0.8847

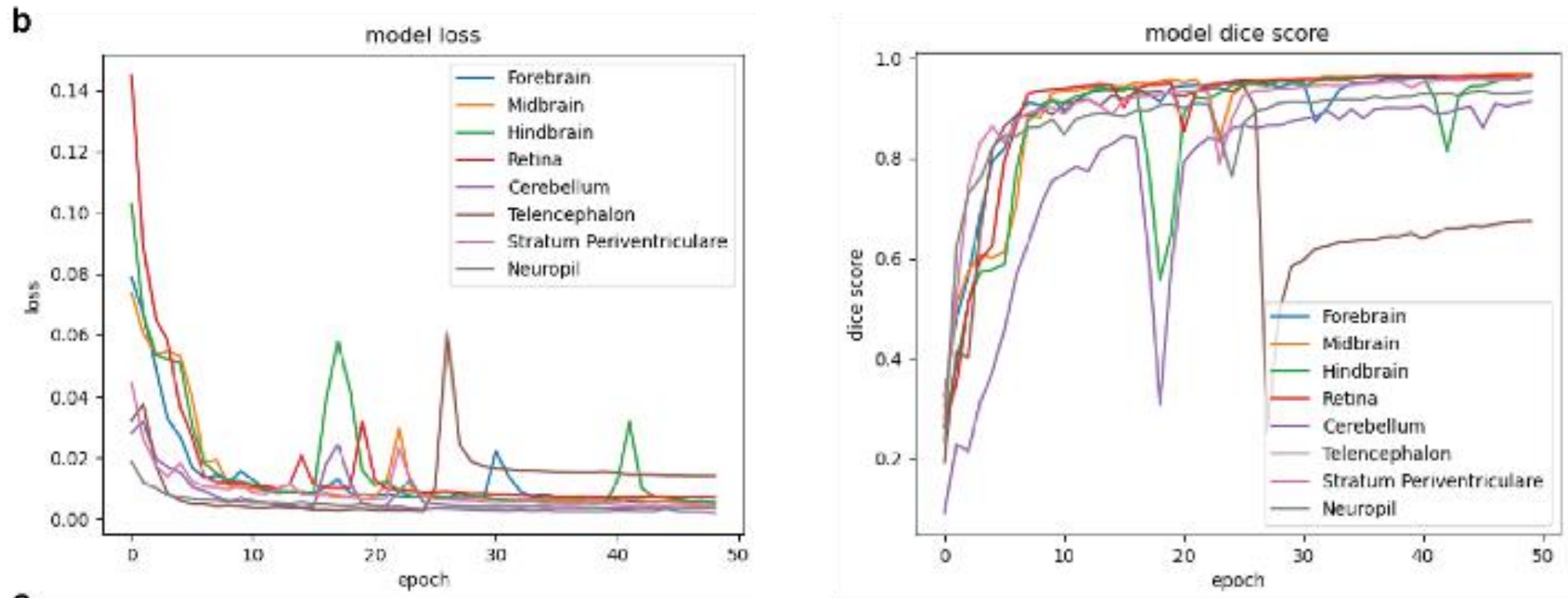

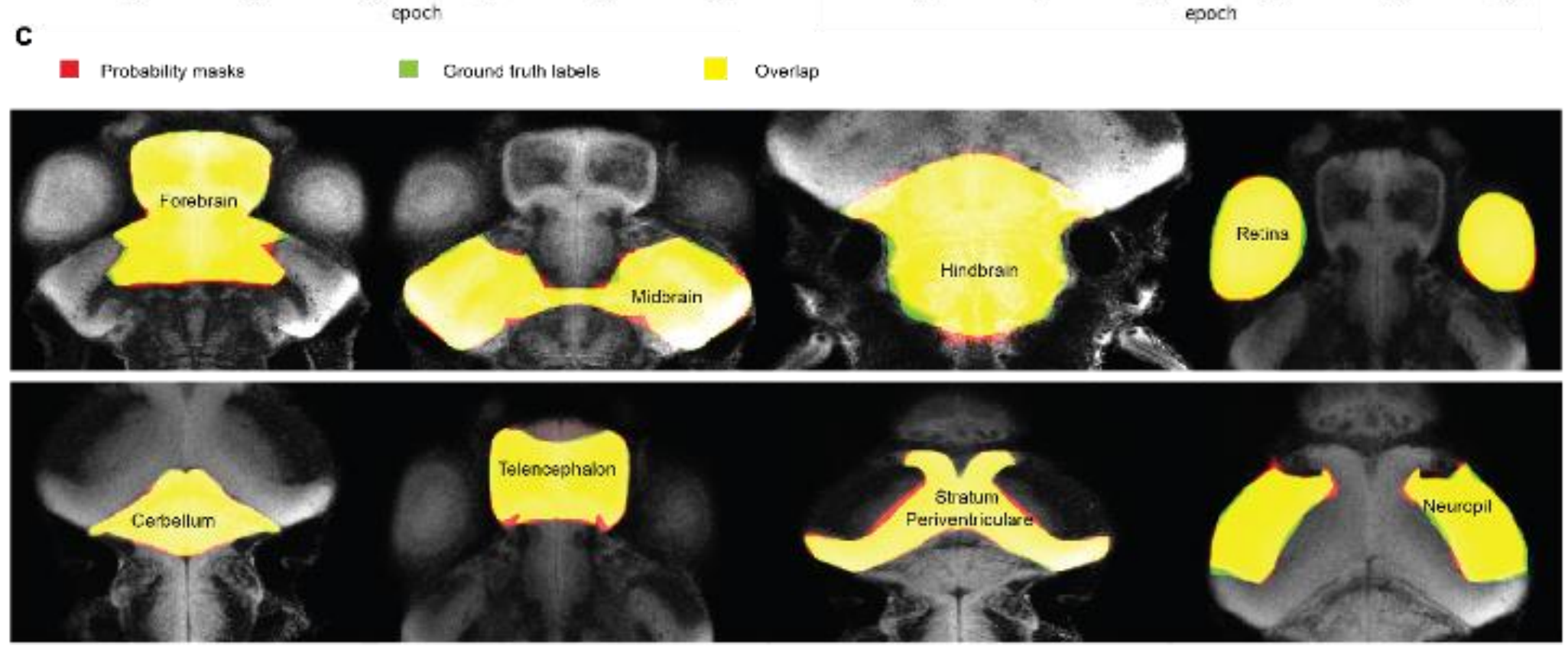

d

Regions	Thickness
Cerebellar Corpus	107
Caudal Hypothalamus	63
Caudal Hindbrain	152
Diencephalon	219
Pallium	123
Rhombomere 1	196
Rhombomere 2	149
Rhombomere 3	166
Rhombomere 4	168
Rhombomere 5	164
Rhombomere 6	178
Rhombomere 7	166
Spinal Cord	99

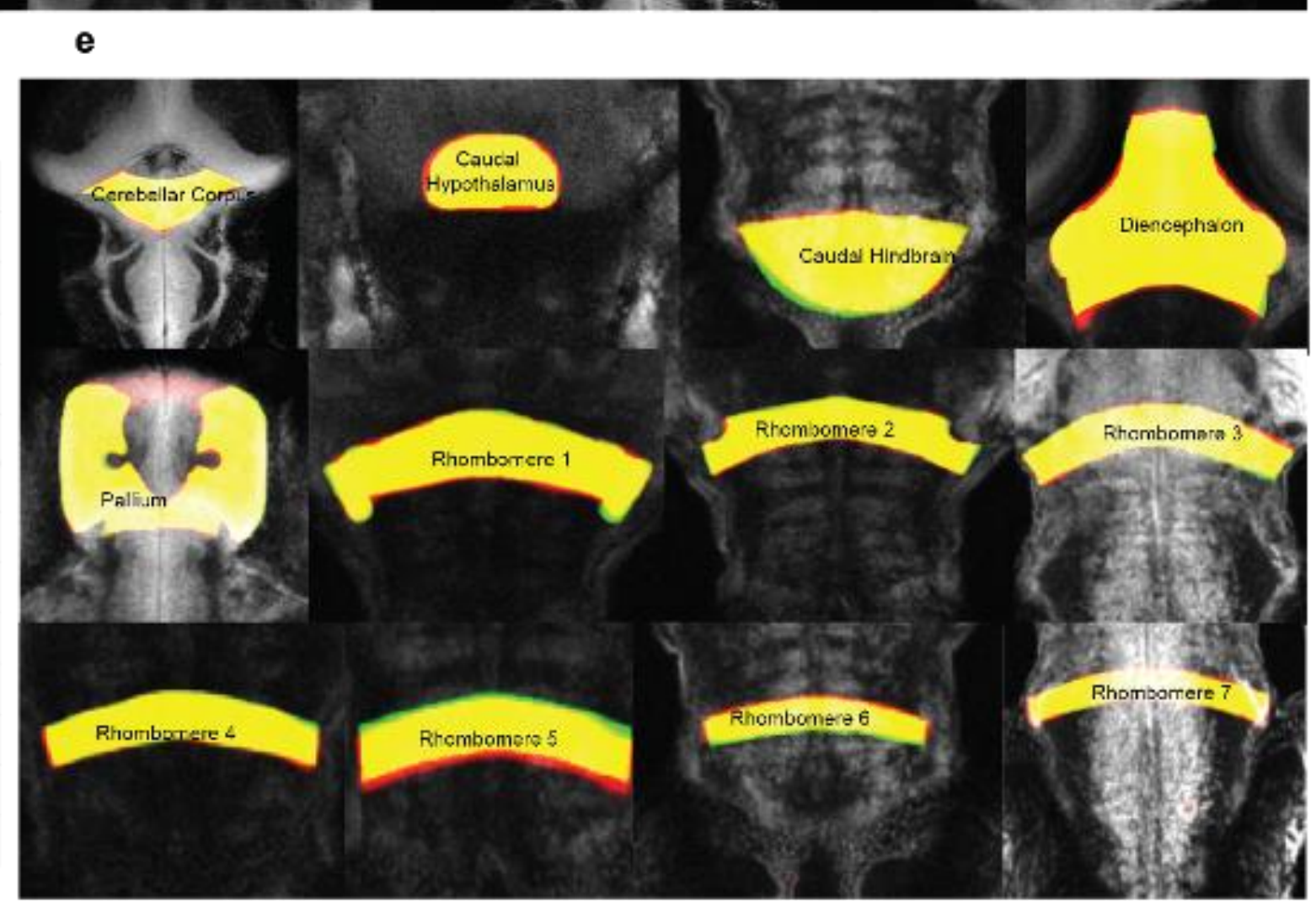

Supplementary Figure 3. Segmentation of brain regions using deep learning

(**a**) Comparison of segmentation results for binary classification of 8 regions with 20%, 25%, 30%, and 35% of images for training, respectively. For each region, a model with the least training data and a dice coefficient above 80% is selected (marked in red). (**b**) Training curves of binary cross-entropy losses and dice scores for the selected models. (**c**) Slice views showing the classifier prediction (red), ground truth (green) and overlap (yellow) for each of the initially annotated 8 regions using the selected model. (**d**) List of the remaining 13 regions and their respective thicknesses (the thickness unit is 1.5um). (**e**) Slice views showing the classifier prediction (red), ground truth (green) and overlap (yellow) for each region.

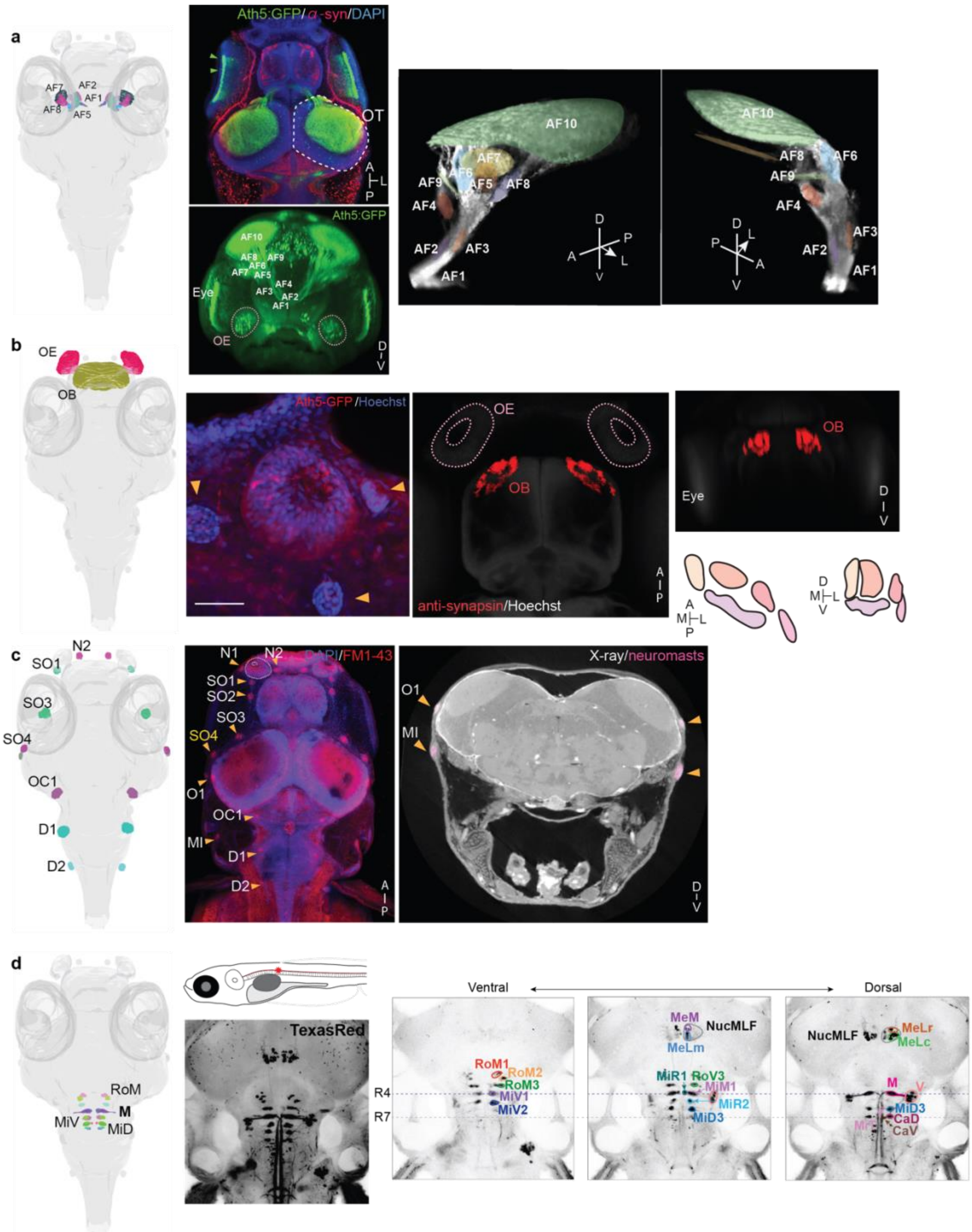

R4

R7

Supplementary Figure 4. MECE masks of sensorimotor pathways

(**a**) Retinotectal projection. (Second to left column) the Tg(*Ath5*) line labels retinal ganglion cell axonal projections and arborization fields (AFs). Anti-synapsin immunostaining allowed identification of AFs. OT: optic tectum. Green arrowheads indicate retinal ganglion cells labeled in the Tg(*Ath5*) line. A: anterior, P: posterior, L: lateral (top). Head view of a 3D reconstruction from confocal imaging of Tg(*Ath5*) larvae. Based on axonal projection patterns and positions, ten AFs were identified (bottom). The Tg line also labels retinal projections and cells in the olfactory epithelium (OE; pink dotted circles). D-V: dorso-ventral axis. (Middle and right columns) lateral views of AF1–9 and neuropil (AF10). (**b**) Olfactory system. (Second to left column) the OE is visualized by Tg(*Ath5*) and Hoechst staining (blue: Hoechst, red: Ath5-GFP). Orange arrowheads indicate surrounding neuromasts. Scale bar: 30 µm. (Right two columns) dorsal and frontal views of the olfactory bulb (OB) visualized by anti-synapsin immunostaining (red). OE neurons project to five olfactory glomeruli in each OB hemisphere. (**c**) Neuromast visualization. (Second to left column) dorsal view of neuromasts labeled with the FM1-43 membrane probe. Orange arrowheads indicate neuromast positions around the head; medaka-specific SO4 neuromasts are highlighted in yellow. N: nasal, SO: supraorbital, O: optic, OC: occipital, MI: middle, D: dorsal neuromasts. (Right) x-ray imaging reveals the protruding structure of neuromasts (pink). (**d**) Reticulospinal projection. (Second to left column) backfill injection into the spinal cord floor plate visualized reticulospinal neurons in the midbrain and hindbrain (bottom; dorsal view). (Right 3 panels) cellular regions of the reticulospinal system were identified based on their relative positions to Mauthner cells (M). Dotted lines indicate corresponding anterior-posterior positions relative to M (rhombomere 4, R4) and the most posterior middle-area cells (rhombomere 7, R7). Identified reticulospinal regions include M (Mauthner cell), RoM (rostral Mauthner cluster), MiM (middle region), MiV (ventral middle region), MiD (dorsal middle region), MiT (T-shaped axon middle region), MiR (rostral middle region), MeM (medial mesencephalic projection), MeLm (lateral-medial mesencephalic projection), MeLr (rostral mesencephalic projection), MeLc (caudal mesencephalic projection), RoV3 (ventral rhombencephalic projection), CaD (dorsal caudal region), CaV (ventral caudal region), NucMLF (nucleus of the medial longitudinal fasciculus), and V (vestibular cluster).

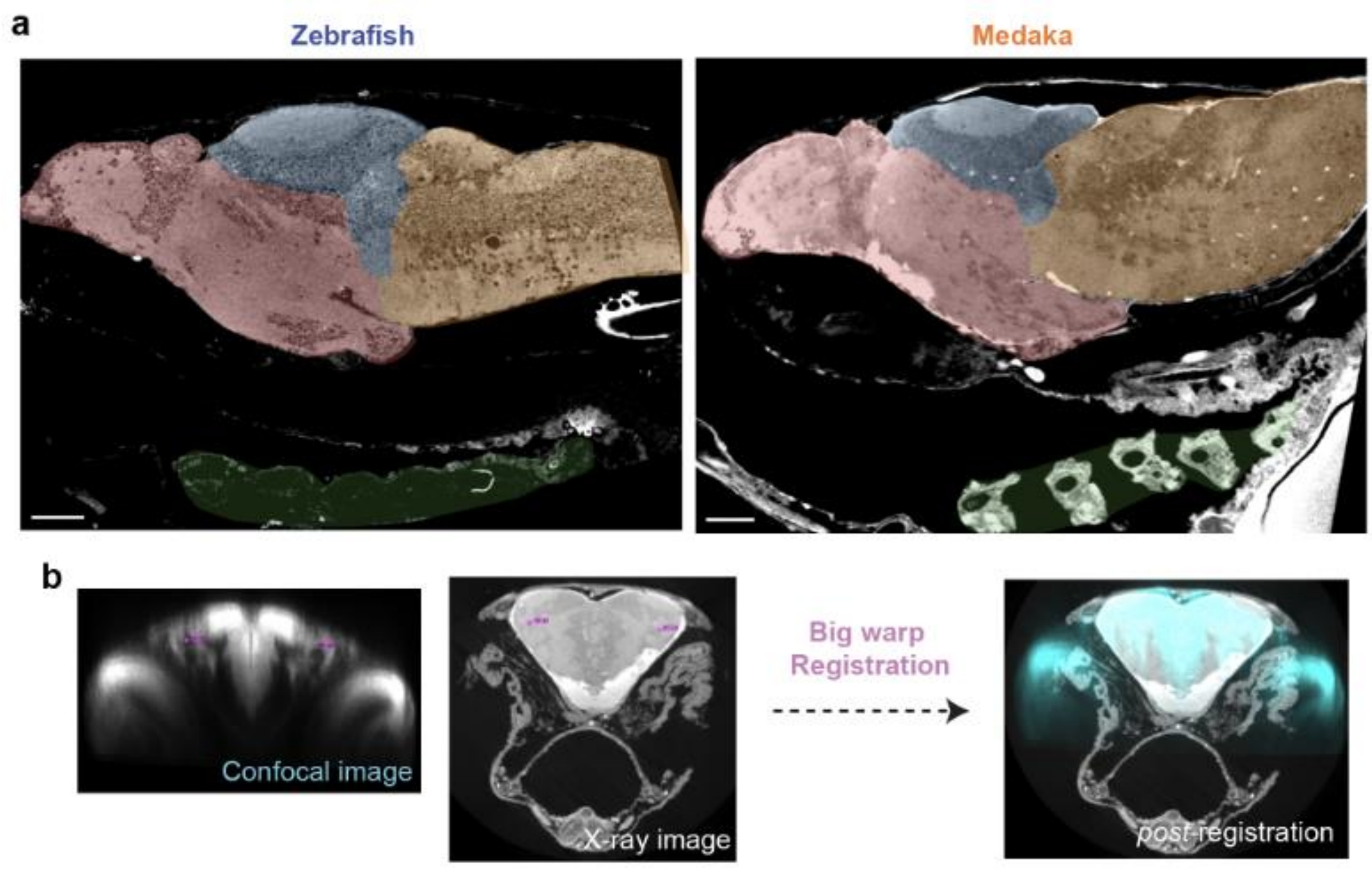

Supplementary Figure 5. X-ray imaging of fish larvae

(**a**) Sagittal sections of larval zebrafish and medaka radiographs. The forebrain (red), midbrain (blue), hindbrain (yellow) and gills (green) are labelled. Scale bar: 50μm.

(**b**) The confocal image stack was registered to the X-ray image stack to align two image stacks from both modalities by large warp registration. Warped confocal stacks were labelled in light blue. Medaka images are shown as an example.

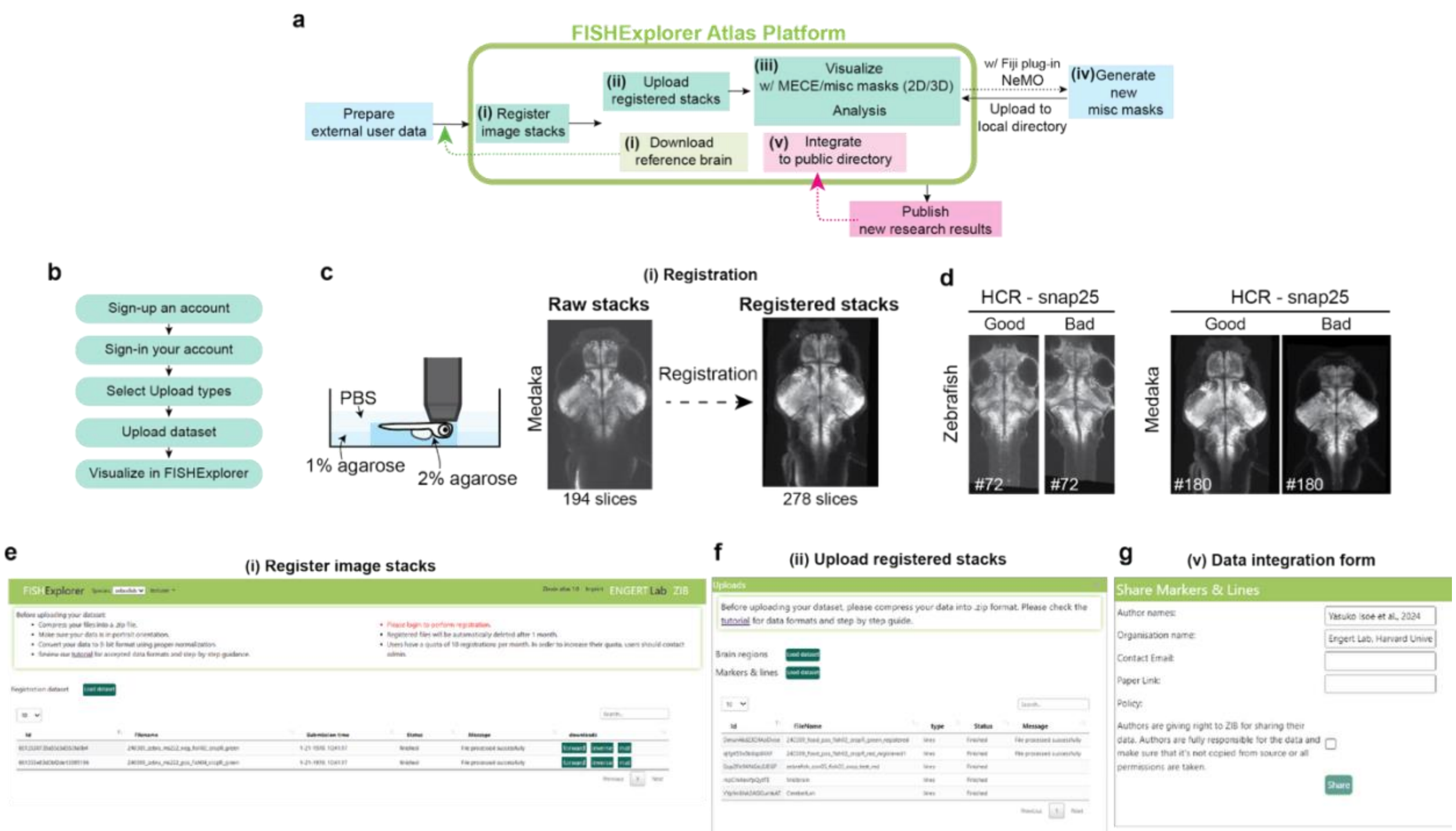

Supplementary Figure 6. Registration protocol

(**a**) Workflow for analysis of own data: After preparing image stacks, (i) users can download the reference brain stacks and Python scripts to register their dataset onto the reference stacks. (ii) The registered brain stacks can then be uploaded to the local private website of their private account and (iii) users can visualise them with existing MECE and MISC masks. (iv) Users can also draw and generate new MISC masks using the Fiji plug-in NeMO and upload these masks to their private account. (v) When a paper is published in a peer-reviewed journal with the user's dataset, the new image stacks and MISC masks can be double-checked by us and added to the public markers & lines and brain region database, respectively, at the user's request. The blue boxes in the chart indicate activities that users perform in their own workspace, while the green boxes indicate the activities that users can perform in the atlas platform.

(**b**) Steps in the FishExplorer atlas platform for uploading custom datasets.

(**c**) To register image stacks to the reference brain, it is important to embed the fish without tilting. Image stacks can be registered using ANTs, CMTK and bigstream (https://github.com/JaneliaSciComp/bigstream).

(**d**) Example images of good and bad registered samples. Number indicated the plane number.

(**e**) Examples of well and poorly registered datasets. The number on the bottom of each image indicates the slice number in the registered stacks.

(**f**) When logged-in users press the "Upload" button, the "Upload window" will show up. Users can then upload brain regions or line datasets; the progress of uploading is displayed in the window.

(**g**) We encourage users to integrate their image stacks and MISC regions after they have analysed their data with the Brain Atlas platform and created a publication. To do this, we ask users to

complete an online form and send a request for integration to the platform maintainer. After validation and curation, the image stacks and MISC masks will get approval and will be integrated into the public directory.

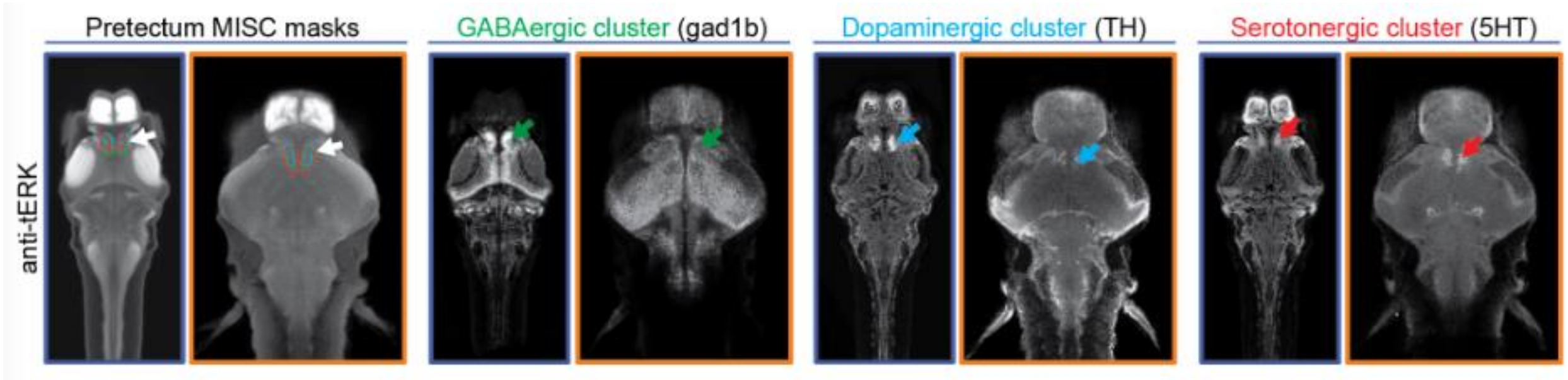

Supplementary Figure 7. Comparative expression of neurotransmitters in pretectum MISC masks

Pretectum MISC masks are shown on the anti-tERK staining image; White arrowheads indicate the location of pretectum MISC masks in zebrafish and medaka. GABAergic pretectum cluster is indicated by green arrowheads, dopaminergic pretectum cluster is indicated by blue arrowheads, and serotonergic pretectum cluster is indicated by red arrowheads.

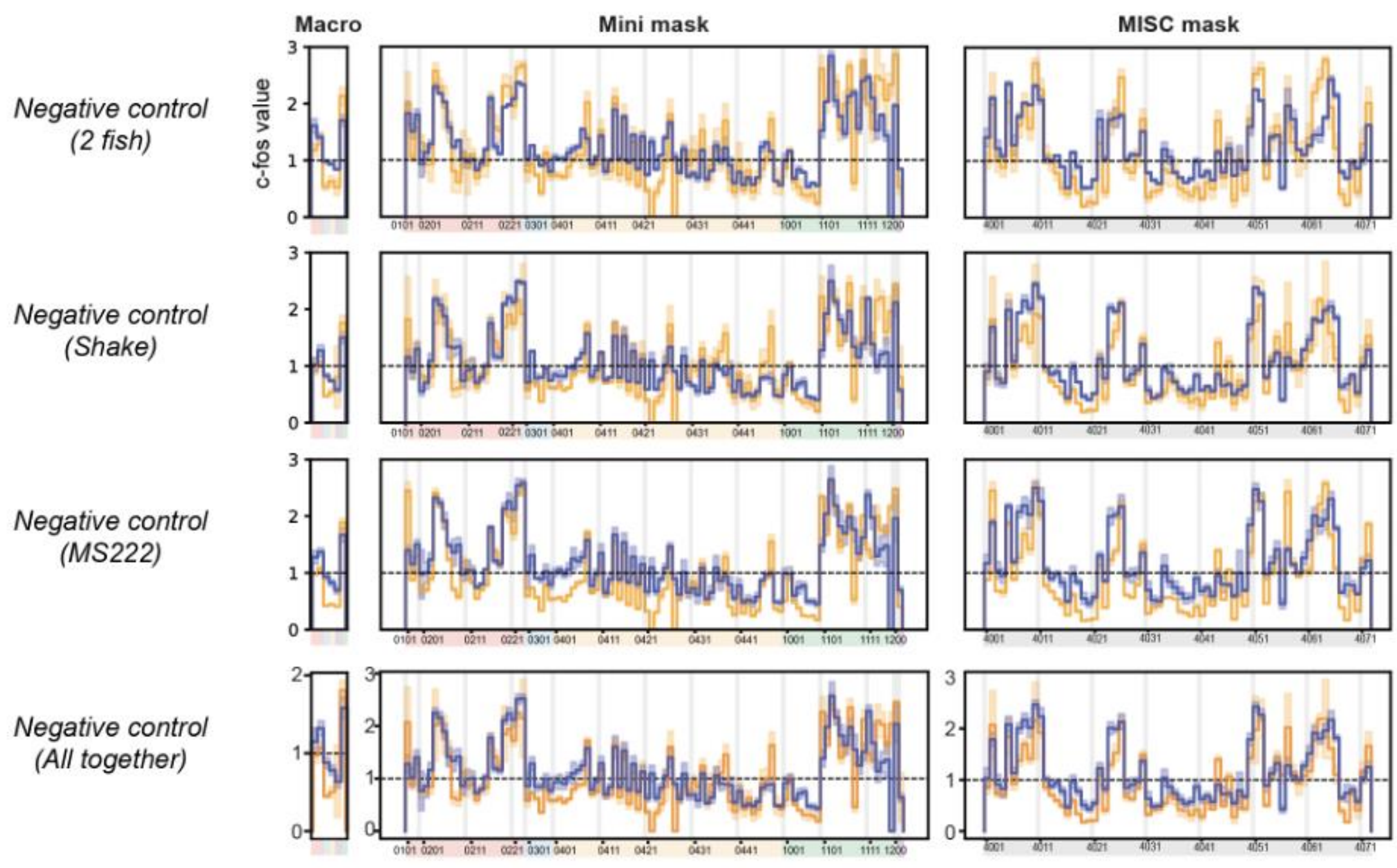

Supplementary Figure 8. Negative controls in *c-fos* expression analysis and MISC masks in the telencephalon.

Negative controls in *c-fos* expression analysis in different conditions. From the top to the bottom: 2 fish condition, Shake condition, MS222 condition and the sum of all the negative controls. Blue: zebrafish, orange: medaka. Shades indicate the standard deviation. *c-fos* value in arbitrary units are shown. Source data are provided as a Source Data file.

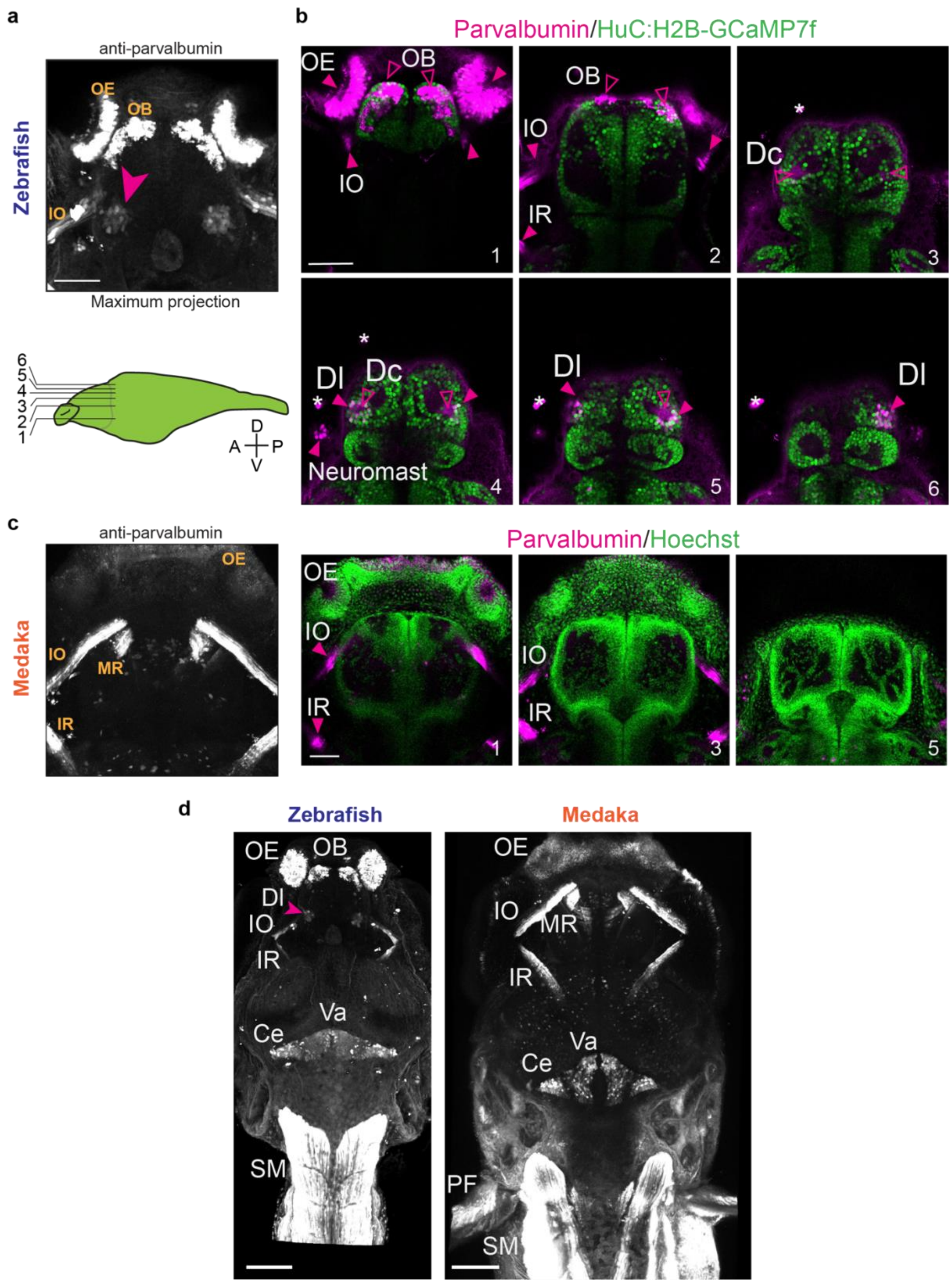

Supplementary Figure 9. Parvalbumin expression in the brain.

(**a**) **Top:** Maximum-intensity projection of anti-parvalbumin (PV) immunostaining in the larval zebrafish telencephalon. The pink arrowhead indicates PV signals in the pallium. Strong PV expression is observed in the olfactory epithelium (OE), olfactory bulb (OB), inferior oblique muscle (IO), and inferior rectus muscle (IR). **Bottom:** Schematic lateral view of the larval zebrafish brain. Horizontal lines indicate the imaging planes shown in **(b)**. Scale bar, 50 µm. A, anterior; P, posterior; D, dorsal; V, ventral.
(b) PV expression at different horizontal planes in the larval zebrafish brain. Magenta, PV; green, Tg(HuC: H2B-GCaMP7f). Imaging plane positions are indicated in the lower right corner of each image. Filled arrowheads indicate cell bodies, and open arrowheads indicate axon fibers. Asterisks indicate nonspecific signals. Dc, central zone of the pallium; Dl, lateral zone of the pallium; IR, inferior rectus muscle. Scale bar, 50 µm.
(c) Left: Maximum-intensity projection of anti-PV immunostaining in the larval medaka telencephalon. MR, medial rectus muscle. **Right:** PV expression at different horizontal planes in the larval medaka brain. Magenta, PV; green, Hoechst. Imaging plane positions are indicated in the lower right corner of each image. Scale bar, 50 µm.
(d) Maximum-intensity projections of anti-PV immunostaining in whole brains of larval zebrafish (left) and medaka (right). The magenta arrowhead indicates PV signal in the zebrafish Dl region. The regions that express PV are labeled. Ce, corpus cerebelli; IO, inferior oblique muscle; IR, inferior rectus muscle; MR, medial rectus muscle; OE, olfactory epithelium; OB, olfactory bulb; PF, pectoral fin; SM, skeletal muscle; Va, valvula cerebelli. Scale bar, 100 µm.

Supplementary Table

Supplementary Table 1. MECE masks

MECE mask IDs, names of the MECE mask IDs, the method that are used to define the masks and references are shown. Tg: transgenic line. Anti-: immunostaining. HCR: *in situ* hybridization.

ID	Macro MECE mask	Mini MECE mask	Methods	References	Notes
	Eye	Hoechst, Tg(Ath5:GFP), Tg(HuC:DsRed)		Different layers are labeled differently by three stainings (not shown in the figure).	Not included in the analysis of volume comparison
0100	Telencephalon				
0200	Diencephalon				
0300	Midbrain (Mesencephalon)				
0400	Hindbrain (Rhombencephalon)				
0500	Spinal cord		Tg(HuC:DsRed), Anti-5 HT, Anti-TH, HCR-ChAT		Tg(vglut:GFP) positive signals in muscles
1000	Ganglia				
0101		Olfactory bulb	Tg(HuC:DsRed), anti-synapsin	(Miyasaka N et al. 2009)	
0102		Pallium	Tg(HuC:DsRed), Tg(vglut:GFP), vglut (HCR)		
0103		Subpallium	Strong HuC:DsRed, gad1b (HCR)	(Okuyama et al. 2013)	
0201		Pineal	Anti-5HT, Islet-1 (HCR)	(Lillesaar 2011)	
0202		Habenula	Tg(HuC:DsRed)	(Aizawa H et al. 2005)	

0203		Optic tract accessory, optic tectum	Tg(Ath5:GFP)	(Robles, Laurell, and Baier 2014)	The boundary between this region and the stratum periventricular is not very clear. Anti 5-HT positive signals around the anterior part of the diencephalon are used to set the boundaries of this region.
0204		Arborization field 1 (AF1)	Tg(Ath5:GFP), Anti-Synapsin	(Robles, Laurell, and Baier 2014)	
0205		Arborization field 1 (AF2)	Tg(Ath5:GFP), Anti-Synapsin	(Robles, Laurell, and Baier 2014)	
0206		Arborization field 1 (AF3)	Tg(Ath5:GFP), Anti-Synapsin	(Robles, Laurell, and Baier 2014)	
0207		Arborization field 1 (AF4)	Tg(Ath5:GFP), Anti-Synapsin	(Robles, Laurell, and Baier 2014)	
0208		Arborization field 1 (AF5)	Tg(Ath5:GFP), Anti-Synapsin	(Robles, Laurell, and Baier 2014)	
0209		Arborization field 1 (AF6)	Tg(Ath5:GFP), Anti-Synapsin	(Robles, Laurell, and Baier 2014)	
0210		Arborization field 1 (AF7)	Tg(Ath5:GFP), Anti-Synapsin	(Robles, Laurell, and Baier 2014)	Outermost part of AF
0211		Arborization field 1 (AF8)	Strong Anti-Synapsin	(Robles, Laurell, and Baier 2014)	
0212		Arborization field 1 (AF9)	Tg(Ath5:GFP), Anti-Synapsin	(Robles, Laurell, and Baier 2014)	
0213		Pretectum	Anti-5-HT, no Tg(HuC:DsRed) signal, gad1b (HCR) cluster	(Antinucci, Folgueira, and Bianco 2019) (Lillesaar 2011)	Connect to AF7 in zebrafish, direct connect or next to AF

0214		Dorsal thalamus	Tg (HuC:DsRed) positive cells in the rostral dorsal thalamus, non-HuC positive cells in the caudal dorsal thalamus. Gad1b negative	(Mueller 2012) (Haehnel-Taguchi et al. 2018)	
0215		Ventral thalamus	Tg (HuC:DsRed) positive cells in the caudal ventral thalamus, Gad1b (HCR) positive	(Mueller 2012) (Haehnel-Taguchi et al. 2018)	
0216		Preoptic area	Anti-TH, Tg(HuC:DsRed)	(Barrios JP et al. 2020)	Anterior to the posterior tuberculum, posterior to subpallium
0217		Posterior tuberculum	Anti-TH, Anti 5-HT, Tg(HuC:DsRed)	(Heap et al. 2018), (Schweitzer et al. 2012) ("Evolution of the Posterior Tuberculum and Preglomerular Nuclear Complex," n.d.) (Lillesaar 2011)	
0218		Preglomerular complex	Presynapsin-positive signals, Tg(HuC:DsRed)	(Bloch et al. 2020), (Wullimann and Umea Salugo 2020), (Kato, Yamada, and Yamamoto 2011)	The Preglomerular complex includes PGl, lateral preglomerular nucleus; PGm, medial preglomerular nucleus.
0219		Rostral hypothalamus	Tg(HuC:DsRed), Anti-5-HT	(Lillesaar 2011)	

0220		Intermediate hypothalamus	Tg(HuC:DsRed), Hoechst, Anti-5 HT clusters in the middle	(Lillesaar 2011)	Ventral to the preglomerular complex. Area between the rostral and caudal hypothalamus.
0221		Diffuse nucleus of the intermediate hypothalamus	Weak Tg(HuC:DsRed), Hoechst	(Heap et al. 2018)	
0222		Caudal hypothalamus	Tg(Ath5:GFP), Anti-5-HT	(Lillesaar 2011)	
0223		Pituitary	Tg(Anti-5HT)	(Lillesaar 2011)	
0301		Torus Longitudinalis	Hoechst	(DeMarco E et al. 2021)	
0302		Torus Semicircularis	Anti-synapsin, Anti-5-HT innervation, vglut(HCR)	(Vanwallegh em G et al. 2017)	
0303		Tectum Neuropil	Projection from Tg(Ath5:GFP), no Hoechst signals	(Del Bene F et al. 2010)	
0304		Tectm Stratum periventricular	Tg(Ath5:GFP), Hoechst	(Del Bene F et al. 2010)	
0305		Tegmentum	Anti-TH (ventral area), Anti-ChAT cluster	(Clemente D et al. 2004)	Bridging between the Torus Semicircular. Posterior to the preglomerular complex
0306		Tegmentum Oculomotor nucleus nIII	Tg(HuC:DsRed), Tg(vglut:GFP), islet-1(HCR)	(Higashijima, Hotta, and Okamoto 2000)	
0307		Tegmentum NucMLF	Backfill	(Thiele, Donovan, and Baier 2014)	
0401		Cerebellum	Tg(HuC:DsRed)	(Bae YK et al. 2009)	
0402		Cerebellum Valvula Cerebelli	Anti-5HT innervation	(Bae YK et al. 2009)	
0403		Cerebellum Cerebellar Corpus		(Bae YK et al. 2009)	
0404		Cerebellum Granular Eminence	Tg(HuC:DsRed)	(Bae YK et al. 2009)	

040 5		Cerebellum Medial caudal lobe	Tg(HuC:DsRe d), Hoechst	(Bae YK et al. 2009)	
040 6		Rhombomere 1	Hoechst		
040 7		Raphe superior	Anti-5-HT	(Teraoka H et al. 2004; Lillesaar 2011)	Rhombomer e 1
040 8		Interpeduncular nucleus	Tg(HuC:DsRe d)	(Aizawa H et al. 2005)	Rhombomer e 1
040 9		Locus Coeruleus	Anti-TH	(Tay TL et al. 2011)	Rhombomer e 1, Noradrenerg ic neurons labeled by Anti-TH
041 0		Rhombomere 2	Hoechst		
041 1		Raphe superior1	Anti-5-HT	(Teraoka H et al. 2004; Lillesaar 2011)	Rhombomer e 1, 2
041 2		Locus Coeruleus 1	Anti-TH	(Tay TL et al. 2011)	Rhombomer e 2, Noradrenerg ic neurons labeled by Anti-TH
041 3		Rhombomere 3	Hoechst		
041 4		Raphe inferior	Anti-5-HT	(Teraoka H et al. 2004; Lillesaar 2011)	Rhombomer e 3
041 5		Rhombomere 4	Hoechst		Mauthner cell locates in Rhombomer e 4
041 6		Raphe inferior 1	Anti-5-HT	(Teraoka H et al. 2004; Lillesaar 2011)	Rhombomer e 4
041 7		Rhombomere 5	Hoechst		
041 8		Raphe inferior 2	Anti-5-HT	(Teraoka H et al. 2004; Lillesaar 2011)	Rhombomer e 5
041 9		Rhombomere 6	Hoechst		
042 0		Raphe inferior 3	Anti-5-HT	(Teraoka H et al. 2004; Lillesaar 2011)	Rhombomer e 6
042 1		Rhombomere 7	Hoechst		
042 2		Raphe inferior 4	Anti-5-HT	(Teraoka H et al. 2004; Lillesaar 2011)	Rhombomer e 7

0423		Caudal Hindbrain			
0424		Caudal Hindbrain Area postrema	Anti-TH	(Ma PM. 1997)	
0425		Caudal Hindbrain Inferior Olive	Tg(HuC:DsRed)	(Felix et al., n.d.)	
0426		Caudal Hindbrain Lateral reticular nucleus	Anti-TH	(Ma PM. 1997)	
0427		RoL-R1	Backfill	(Metcalfe, Mendelson, and Kimmel 1986)	Rhombomere 1
0428		RoL2	Backfill	(Metcalfe, Mendelson, and Kimmel 1986)	Rhombomere 2
0429		RoM1	Backfill	(Metcalfe, Mendelson, and Kimmel 1986)	Rhombomere 1
0430		RoM2	Backfill	(Metcalfe, Mendelson, and Kimmel 1986)	Rhombomere 2
0431		RoM3	Backfill	(Metcalfe, Mendelson, and Kimmel 1986)	Rhombomere 3
0432		RoL3	Backfill	(Metcalfe, Mendelson, and Kimmel 1986)	Rhombomere 3
0433		RoV3	Backfill	(Metcalfe, Mendelson, and Kimmel 1986)	Rhombomere 3
0434		Mauthner	Backfill	(Metcalfe, Mendelson, and Kimmel 1986)	Rhombomere 4
0435		Mauthner_cell_Axon_Cap	Anti-synapsin	(Bierman, Zottoli, and Hale 2009)	Rhombomere 4
0436		MiV1	Backfill	(Metcalfe, Mendelson, and Kimmel 1986)	Rhombomere 4
0437		MiM1	Backfill	(Metcalfe, Mendelson, and Kimmel 1986)	Rhombomere 4
0438		MiR1	Backfill	(Metcalfe, Mendelson, and Kimmel 1986)	Rhombomere 4
0439		MiR2	Backfill	(Metcalfe, Mendelson, and Kimmel 1986)	Rhombomere 5

0440		MiD2	Backfill	(Metcalfe, Mendelson, and Kimmel 1986)	Medaka doesn't have MiD2. Rhombomere 5
0441		MiV2	Backfill	(Metcalfe, Mendelson, and Kimmel 1986)	Rhombomere 5
0442		MiD3	Backfill	(Metcalfe, Mendelson, and Kimmel 1986)	Rhombomere 6
0443		MiT	Backfill	(Metcalfe, Mendelson, and Kimmel 1986)	Rhombomere 6
0444		CaD	Backfill	(Metcalfe, Mendelson, and Kimmel 1986)	Rhombomere 7
0445		CaV	Backfill	(Metcalfe, Mendelson, and Kimmel 1986)	Rhombomere 7
0446		Spinal Backfill Vestibular Population	Backfill	(Metcalfe, Mendelson, and Kimmel 1986)	
0447		Tangential Vestibular nucleus		(Metcalfe, Mendelson, and Kimmel 1986)	
0448		Tangential Vestibular nucleus1	Backfill	(Metcalfe, Mendelson, and Kimmel 1986)	
0449		Medial Vestibular nucleus	Backfill	(Metcalfe, Mendelson, and Kimmel 1986)	
0450		Medial Vestibular nucleus1	Backfill	(Metcalfe, Mendelson, and Kimmel 1986)	
1001		Oculomotor nucleus nIII	Tg(vglut:GFP), islet-1 (HCR)	(Higashijima, Hotta, and Okamoto 2000)	Rhombomere 1, cranial nerve
1002		Oculomotor nucleus nIV	Tg(vglut:GFP), islet-1 (HCR)	(Higashijima, Hotta, and Okamoto 2000)	Rhombomere 1, cranial nerve
1003		Anterior_Cluster_of_nV_Trigenimal_Motorneruons	Islet-1 (HCR)	(Higashijima, Hotta, and Okamoto 2000)	
1004		Posterior_Cluster_of_nV_Trigenimal_motor_neruons	Islet-1 (HCR)	(Higashijima, Hotta, and Okamoto 2000)	

1005		VII_Facial_Motor_and_octavolateralis_efferent_neuron	Islet-1 (HCR)	(Higashijima, Hotta, and Okamoto 2000)	
1006		VII_Facial_Motor_and_octavolateralis_efferent_neuron1	Islet-1 (HCR)	(Higashijima, Hotta, and Okamoto 2000)	
1007		VII_Facial_Motor_and_octavolateralis_efferent_neuron	Islet-1 (HCR)	(Higashijima, Hotta, and Okamoto 2000)	
1008		Caudal Hindbrain X-vagus motor neuron cluster	Anti-TH, islet-1 (HCR)	(Higashijima, Hotta, and Okamoto 2000)	Cranial nerve (X)
1101		Olfactory epithelium	Tg(vglut:GFP), Hoechst, Tg(Ath5:GFP)		
1102		Facial sensory ganglion	Tg(HuC:DsRed)	(Higashijima, Hotta, and Okamoto 2000)	
1103		Facial Glossopharyngeal ganglion (glX)	Hoechst, islet-1(HCR)	(Cox et al. 2011)	Connected to Cranial nerve (IX)
1104		Statoacoustic ganglion (Vlll)	Tg (vglut:GFP)	(Higashijima, Hotta, and Okamoto 2000)	The sensory ganglion of the ear. Connected to Cranial nerve (Vlll)
1105		Trigeminal ganglion	Tg(Ath5:GFP), Hoechst	(Pan YA et al. 2012)	Connected to Cranial nerve (V)
1106		Anterior lateral line ganglion	Tg(Ath5:GFP), Hoechst	(Haehnel-Taguchi et al. 2018; Manuel et al. 2021)	
1107		*Posterior lateral line ganglion*	Hoechst	(Metcalfe WK et al. 1985)	
1108		Lateral_line_Neuromast_D1	FM1-34, anti-TH	(Haehnel-Taguchi et al. 2018; Manuel et al. 2021)	
1109		Lateral_line_Neuromast_D2	FM1-34, anti-TH	(Haehnel-Taguchi et al. 2018; Manuel et al. 2021)	
1110		Lateral_line_Neuromast_N	FM1-34, anti-TH	(Haehnel-Taguchi et al. 2018; Manuel et al. 2021)	

1111		Lateral_line_Neuromast_O1	FM1-34, anti-TH	(Haehnel-Taguchi et al. 2018; Manuel et al. 2021)	
1112		Lateral_line_Neuromast_OC1	FM1-34, anti-TH	(Haehnel-Taguchi et al. 2018; Manuel et al. 2021)	
1113		Lateral_line_Neuromast_SO1	FM1-34, anti-TH	(Haehnel-Taguchi et al. 2018; Manuel et al. 2021)	
1114		Lateral_line_Neuromast_SO2	FM1-34, anti-TH	(Haehnel-Taguchi et al. 2018; Manuel et al. 2021)	
1115		Lateral_line_Neuromast_SO3	FM1-34, anti-TH	(Haehnel-Taguchi et al. 2018; Manuel et al. 2021)	
1116		Lateral_line_Neuromast_SO4	FM1-34, anti-TH	(Haehnel-Taguchi et al. 2018; Manuel et al. 2021)	Zebrafish doesn’t have SO4
1200		Vagus ganglion (gX)	Hoechst , islet-1(HCR)	(Higashijima, Hotta, and Okamoto 2000)	Connected to Cranial nerve (X)

Supplementary Table 2. Mini masks - defined with expression patterns.

Misc mask IDs, names of the misc mask IDs, the method that are used to define the masks and references are shown. Tg: transgenic line. Anti-: immunostaining. HCR: *in situ* hybridization.

ID	Gene	Methods	MISC region name	Notes	Reference
4001	Glutamatergic neuronal cluster	HCR slc17a6b_vglut2	Forebrain - Telencephalon - Vglut2 rind	Signal location in medaka matched to that in zebrafish	(Satou et al. 2013; Filippi et al 2014)
4002	Glutamatergic neuronal cluster	HCR slc17a6b_vglut2	Forebrain - Telencephalon - Subpallial Vglut2 Cluster	Signal location in medaka matched to that in zebrafish	(Satou et al. 2013; Filippi et al 2014)
4003	Glutamatergic neuronal cluster	HCR slc17a6b_vglut2	Forebrain - Diencephalon - Left Habenula Vglut2 Cluster	Signal location in medaka matched to that in zebrafish	(Satou et al. 2013; Filippi et al 2014)
4004	Glutamatergic neuronal cluster	HCR slc17a6b_vglut2	Forebrain - Diencephalon - Right Habenula Vglut2 Cluster	Signal location in medaka matched to that in zebrafish	(Satou et al. 2013; Filippi et al 2014)
4005	Glutamatergic neuronal cluster	HCR slc17a6b_vglut2	Forebrain - Diencephalon - Preoptic area Vglut2 cluster	Signal location in medaka matched to that in zebrafish	(Satou et al. 2013; Filippi et al 2014)
4006	Glutamatergic neuronal cluster	HCR slc17a6b_vglut2	Forebrain - Diencephalon - Medial vglut2 cluster	Signal location in medaka matched to that in zebrafish	(Satou et al. 2013; Filippi et al 2014)
4007	Glutamatergic neuronal cluster	HCR slc17a6b_vglut2	Forebrain - Diencephalon - Hypothalamus Vglut2 Cluster 1	Signal location in medaka matched to that in zebrafish	(Satou et al. 2013; Filippi et al 2014)
4008	Glutamatergic neuronal cluster	HCR slc17a6b_vglut2	Forebrain - Diencephalon - Hypothalamus Vglut2 Cluster 2	Signal location in medaka matched to that in zebrafish	(Satou et al. 2013; Filippi et al 2014)
4009	Glutamatergic neuronal cluster	HCR slc17a6b_vglut2	Forebrain - Diencephalon - Hypothalamus Vglut2 Cluster 3	Signal location in medaka matched to that in zebrafish	(Satou et al. 2013; Filippi et al 2014)

4011	Glutamatergic neuronal cluster	HCR slc17a6b_vglut2	Forebrain - Diencephalon - Hypothalamus Vglut2 Cluster 5	Signal location in medaka matched to that in zebrafish	(Satou et al. 2013; Filippi et al 2014)
4012	Glutamatergic neuronal cluster	HCR slc17a6b_vglut2	Forebrain - Diencephalon - Hypothalamus Vglut2 Cluster 6	Signal location in medaka matched to that in zebrafish	(Satou et al. 2013; Filippi et al 2014)
4013	Glutamatergic neuronal cluster	HCR slc17a6b_vglut2	Midbrain - Vglut2 cluster 1	Signal location in medaka matched to that in zebrafish	(Satou et al. 2013; Filippi et al 2014)
4014	Glutamatergic neuronal cluster	HCR slc17a6b_vglut2	Hindbrain - Cerebellum-Vglut2 enriched areas	Signal location in medaka matched to that in zebrafish	(Satou et al. 2013; Filippi et al 2014)
4015	Glutamatergic neuronal cluster	HCR slc17a6b_vglut2	Hindbrain - Vglut2 cluster 1	Signal location in medaka matched to that in zebrafish	(Satou et al. 2013; Filippi et al 2014)
4016	Glutamatergic neuronal cluster	HCR slc17a6b_vglut2	Hindbrain - Vglut2 cluster 2	Signal location in medaka matched to that in zebrafish	(Satou et al. 2013; Filippi et al 2014)
4017	Glutamatergic neuronal cluster	HCR slc17a6b_vglut2	Hindbrain - Vglut2 cluster 3	Signal location in medaka matched to that in zebrafish	(Satou et al. 2013; Filippi et al 2014)
4018	Glutamatergic neuronal cluster	HCR slc17a6b_vglut2	Hindbrain - Vglut2 cluster 4	Signal location in medaka matched to that in zebrafish	(Satou et al. 2013; Filippi et al 2014)
4019	Glutamatergic neuronal cluster	HCR slc17a6b_vglut2	Hindbrain - Vglut2 Stripe 1	Signal location in medaka matched to that in zebrafish	(Satou et al. 2013; Filippi et al 2014)
4020	Glutamatergic neuronal cluster	HCR slc17a6b_vglut2	Hindbrain - Vglut2 Stripe 2	Signal location in medaka matched to that in zebrafish	(Satou et al. 2013; Filippi et al 2014)
4021	Glutamatergic neuronal cluster	HCR slc17a6b_vglut2	Hindbrain - Vglut2 Stripe 3	Signal location in medaka matched to that in zebrafish	(Satou et al. 2013; Filippi et al 2014)
4022	Glutamatergic neuronal cluster	HCR slc17a6b_vglut2	Hindbrain - Vglut2 Stripe 4	Signal location in medaka matched to that in zebrafish	(Satou et al. 2013; Filippi et al 2014)
4023	GABAergic neuronal cluster	HCR GAD1b	Forebrain - Telencephalon - Subpallial Gad1b cluster	Signal location in medaka matched to that in zebrafish	(Satou et al. 2013; Filippi et al 2014)
4024	GABAergic neuronal cluster	HCR GAD1b	Forebrain - Diencephalon - Pretectal Gad1b Cluster	Signal location in medaka matched to that in zebrafish	(Satou et al. 2013; Filippi et al 2014)

4025	GABAergic neuronal cluster	HCR GAD1b	Forebrain - Diencephalon - Hypothalamus Gad1b Cluster 1	Signal location in medaka matched to that in zebrafish	(Satou et al. 2013; Filippi et al 2014)
4026	GABAergic neuronal cluster	HCR GAD1b	Forebrain - Diencephalon - Hypothalamus Gad1b Cluster 2	Signal location in medaka matched to that in zebrafish	(Satou et al. 2013; Filippi et al 2014)
4027	GABAergic neuronal cluster	HCR GAD1b	Forebrain - Diencephalon - Hypothalamus Gad1b Cluster 3 Sparse	Signal location in medaka matched to that in zebrafish	(Satou et al. 2013; Filippi et al 2014)
4028	GABAergic neuronal cluster	HCR GAD1b	Cerebellum enriched areas	Signal location in medaka matched to that in zebrafish	(Satou et al. 2013; Filippi et al 2014)
4029	GABAergic neuronal cluster	HCR GAD1b	Hindbrain - Gad1b Cluster 1	Signal location in medaka matched to that in zebrafish	(Satou et al. 2013; Filippi et al 2014)
4030	GABAergic neuronal cluster	HCR GAD1b	Hindbrain - Gad1b Cluster 2	Signal location in medaka matched to that in zebrafish	(Satou et al. 2013; Filippi et al 2014)
4031	GABAergic neuronal cluster	HCR GAD1b	Hindbrain - Gad1b Cluster 3	Signal location in medaka matched to that in zebrafish	(Satou et al. 2013; Filippi et al 2014)
4032	GABAergic neuronal cluster	HCR GAD1b	Hindbrain - Gad1b Cluster 4	Signal location in medaka matched to that in zebrafish	(Satou et al. 2013; Filippi et al 2014)
4033	GABAergic neuronal cluster	HCR GAD1b	Hindbrain - Gad1b Cluster 5	Signal location in medaka matched to that in zebrafish	(Satou et al. 2013; Filippi et al 2014)
4034	GABAergic neuronal cluster	HCR GAD1b	Hindbrain - Gad1b Cluster 6	Signal location in medaka matched to that in zebrafish	(Satou et al. 2013; Filippi et al 2014)
4035	GABAergic neuronal cluster	HCR GAD1b	Hindbrain - Gad1b Cluster 7	Signal location in medaka matched to that in zebrafish	(Satou et al. 2013; Filippi et al 2014)
4036	GABAergic neuronal cluster	HCR GAD1b	Hindbrain - Gad1b Cluster 8	Signal location in medaka matched to that in zebrafish	(Satou et al. 2013; Filippi et al 2014)
4037	GABAergic neuronal cluster	HCR GAD1b	Hindbrain - Gad1b Cluster 9	Signal location in medaka matched to that in zebrafish	(Satou et al. 2013; Filippi et al 2014)
4038	GABAergic neuronal cluster	HCR GAD1b	Hindbrain - Gad1b Cluster 10	Signal location in medaka matched to that in zebrafish	(Satou et al. 2013; Filippi et al 2014)

4039	GABAergic neuronal cluster	HCR GAD1b	Hindbrain - Gad1b Cluster 11	Signal location in medaka matched to that in zebrafish	(Satou et al. 2013; Filippi et al 2014)
4040	GABAergic neuronal cluster	HCR GAD1b	Hindbrain - Gad1b Cluster 12	Signal location in medaka matched to that in zebrafish	(Satou et al. 2013; Filippi et al 2014)
4041	GABAergic neuronal cluster	HCR GAD1b	Hindbrain - Gad1b Cluster 13	Signal location in medaka matched to that in zebrafish	(Satou et al. 2013; Filippi et al 2014)
4042	GABAergic neuronal cluster	HCR GAD1b	Hindbrain - Gad1b Cluster 14	Signal location in medaka matched to that in zebrafish	(Satou et al. 2013; Filippi et al 2014)
4043	GABAergic neuronal cluster	HCR GAD1b	Hindbrain - Gad1b Cluster 15	Signal location in medaka matched to that in zebrafish	(Satou et al. 2013; Filippi et al 2014)
4044	GABAergic neuronal cluster	HCR GAD1b	Hindbrain - Gad1b Cluster 16	Signal location in medaka matched to that in zebrafish	(Satou et al. 2013; Filippi et al 2014)
4045	GABAergic neuronal cluster	HCR GAD1b	Hindbrain - Gad1b Cluster 17	Signal location in medaka matched to that in zebrafish	(Satou et al. 2013; Filippi et al 2014)
4046	GABAergic neuronal cluster	HCR GAD1b	Hindbrain - Gad1b Cluster 18	Signal location in medaka matched to that in zebrafish	(Satou et al. 2013; Filippi et al 2014)
4047	GABAergic neuronal cluster	HCR GAD1b	Hindbrain - Gad1b Cluster 19	Signal location in medaka matched to that in zebrafish	(Satou et al. 2013; Filippi et al 2014)
4048	GABAergic neuronal cluster	HCR GAD1b	Hindbrain - Gad1b Cluster 20	Signal location in medaka matched to that in zebrafish	(Satou et al. 2013; Filippi et al 2014)
4049	Serotonergic neuronal cluster	Anti-5HT	Forebrain - Diencephalon - Pineal - 5HT Cluster	Signal location in medaka matched to that in zebrafish	(McLean DL, Fetcho JR, 2004; Lillesaar C, 2011)
4050	Serotonergic neuronal cluster	Anti-5HT	Forebrain - Diencephalon - Pretectum - 5HT Cluster	Signal location in medaka matched to that in zebrafish	(McLean DL, Fetcho JR, 2004; Lillesaar C, 2011)
4051	Serotonergic neuronal cluster	Anti-5HT	Forebrain - Diencephalon - Posterior Tuberculum - 5HT Cluster	Signal location in medaka matched to that in zebrafish	(McLean DL, Fetcho JR, 2004; Lillesaar C, 2011)

4052	Serotonergic neuronal cluster	Anti-5HT	Forebrain - Diencephalon - Intermediate Hypothalamus - 5HT Cluster	Signal location in medaka matched to that in zebrafish	(McLean DL, Fetcho JR, 2004; Lillesaar C, 2011)
4053	Serotonergic neuronal cluster	Anti-5HT	Forebrain - Diencephalon - Caudal Hypothalamus - 5HT Cluster	Signal location in medaka matched to that in zebrafish	(McLean DL, Fetcho JR, 2004; Lillesaar C, 2011)
4054	Serotonergic neuronal cluster	Anti-5HT	Hindbrain - Cerebellar Corpus - 5HT Cluster	Signal location in medaka matched to that in zebrafish	(McLean DL, Fetcho JR, 2004; Lillesaar C, 2011)
4055	Serotonergic neuronal cluster	Anti-5HT	Hindbrain - Raphe Inferior - 5HT Cluster	Signal location in medaka matched to that in zebrafish	(McLean DL, Fetcho JR, 2004; Lillesaar C, 2011)
4056	Serotonergic neuronal cluster	Anti-5HT	Hindbrain - Raphe Superior - 5HT Cluster	Signal location in medaka matched to that in zebrafish	(McLean DL, Fetcho JR, 2004; Lillesaar C, 2011)
4057	Serotonergic neuronal cluster	Anti-5HT	Hindbrain - Caudal Hindbrain - 5HT Cluster	Signal location in medaka matched to that in zebrafish	(McLean DL, Fetcho JR, 2004; Lillesaar C, 2011)
4058	Dopaminergic neuronal cluster	Anti-TH	Forebrain - Telencephalon - Olfactory bulb - TH cluster	Signal location in medaka matched to that in zebrafish	(Tay et al. 2011; Schweitzer et al. 2012; Matsui 2017)
4059	Dopaminergic neuronal cluster	Anti-TH	Forebrain - Telencephalon - Subapallium - TH cluster	Signal location in medaka matched to that in zebrafish	(Tay et al. 2011; Schweitzer et al. 2012; Matsui 2017)
4060	Dopaminergic neuronal cluster	Anti-TH	Forebrain - Diencephalon - Ventral Thalamic Periventricular Posterior Tuberculum - TH cluster 1	Signal location in medaka matched to that in zebrafish	(Tay et al. 2011; Schweitzer et al. 2012; Matsui 2017)
4061	Dopaminergic neuronal cluster	Anti-TH	Forebrain - Diencephalon - Posterior Tuberculum - TH cluster 2	Signal location in medaka matched to that in zebrafish	(Tay et al. 2011; Schweitzer et al. 2012; Matsui 2017)
4062	Dopaminergic neuronal cluster	Anti-TH	Forebrain - Diencephalon - Hypothalamus - TH cluster 3	Signal location in medaka matched to that in zebrafish	(Tay et al. 2011; Schweitzer et al. 2012; Matsui 2017)

4063	Dopaminergic neuronal cluster	Anti-TH	Forebrain - Diencephalon - Posterior Tuberculum Hypothalamus - TH cluster 4 5	Signal location in medaka matched to that in zebrafish	(Tay et al. 2011; Schweitzer et al. 2012; Matsui 2017)
4064	Dopaminergic neuronal cluster	Anti-TH	Forebrain - Diencephalon - Hypothalamus - TH cluster 6	Signal location in medaka matched to that in zebrafish	(Tay et al. 2011; Schweitzer et al. 2012; Matsui 2017)
4065	Dopaminergic neuronal cluster	Anti-TH	Forebrain - Diencephalon - Caudal Hypothalamus - TH cluster 7	Signal location in medaka matched to that in zebrafish	(Tay et al. 2011; Schweitzer et al. 2012; Matsui 2017)
4066	Dopaminergic neuronal cluster	Anti-TH	Forebrain - Diencephalon - Anterior Preoptic Area - TH cluster	Signal location in medaka matched to that in zebrafish	(Tay et al. 2011; Schweitzer et al. 2012; Matsui 2017)
4067	Dopaminergic neuronal cluster	Anti-TH	Forebrain - Diencephalon - Preoptic Area - TH cluster	Signal location in medaka matched to that in zebrafish	(Tay et al. 2011; Schweitzer et al. 2012; Matsui 2017)
4068	Dopaminergic neuronal cluster	Anti-TH	Forebrain - Diencephalon - Pretectum - TH cluster	Signal location in medaka matched to that in zebrafish	(Tay et al. 2011; Schweitzer et al. 2012; Matsui 2017)
4069	Noradrenergic neuronal cluster	Anti-TH	Hindbrain - Caudal Hindbrain Small clusters - TH cluster	Signal location in medaka matched to that in zebrafish	(Tay et al. 2011; Schweitzer et al. 2012; Matsui 2017)
4070	Noradrenergic neuronal cluster	Anti-TH	Hindbrain - Locus Coeruleus - TH cluster	Signal location in medaka matched to that in zebrafish	(Tay et al. 2011; Schweitzer et al. 2012; Matsui 2017)
4071	Noradrenergic neuronal cluster	Anti-TH	Hindbrain - Medulla Oblongata - TH cluster	Signal location in medaka matched to that in zebrafish	(Tay et al. 2011; Schweitzer et al. 2012; Matsui 2017)
4072	Oxytocinergic neuronal cluster	HCR Oxt	Forebrain - Diencephalon - Posterior Tuberculum - Oxt cluster	Signal location in medaka matched to that in zebrafish	(Wee CL et al 2019)
4073	Oxytocinergic neuronal cluster	HCR Oxt	Forebrain - Diencephalon - Preoptic Area - Oxt cluster	Signal location in medaka matched to that in zebrafish	(Wee CL et al 2019)

Supplementary Table 3. Mini masks - defined anatomically.

Misc mask IDs, names of the misc mask IDs, the method that are used to define the masks and references are shown. Tg: transgenic line. Anti-: immunostaining. HCR: *in situ* hybridization.

**ID**	**Misc mask**	**Visualizing Methods**	**References**	**Overlapping MECE masks**	**Notes**
3001	Dl	Anti:Synapsin, Tg(HuC:DsRed), anti:parvalbumin	(Isoe et al., 2023, Muller et al 2011)		(zebrafish) Parvalbumin positive signals in cell bodies in the posterior half of the pallium. Using this compartment boudaries, the Dl region is define in the pallium.
3002	Dm	Anti:Synapsin, Tg(HuC:DsRed)	(Isoe et al., 2023)	Pallium	
3003	Dc	Anti:Synapsin, Tg(HuC:DsRed), anti:parvalbumin	(Isoe et al., 2023, Muller et al 2011)	Pallium	(zebrafish) Parvalbumin positive signals in axon bundle in the posterior half of the pallium. Using this compartment boudaries, the Dc region is define in the pallium.
3004	Dp	Anti:Synapsin, Tg(HuC:DsRed)	(Isoe et al., 2023)	Pallium	
3005	Dd	Strong Anti:Synapsin, Tg(HuC:DsRed)	(Isoe et al., 2023)	Pallium	(medaka) Strong signals of Anti:Synapsin signals
3006	Dm4	Strong Anti:Synapsin, Tg(HuC:DsRed)	(Isoe et al., 2023)	Pallium	(medaka) Strong signals of Anti:Synapsin signals (not shown).
3007	Migrated area 4 (M4)	Tg(HuC:DsRed)	(Wullimann 2022; Mueller, Wullimann, and Guo 2008)	Pallium	HuC positive region lateral to the anterior commissure
3008	Olfactory glomeruli	Anti:Synapsin	(Miyasaka et al. 2014)	Olfactory bulb	Supp.fig.1(iv)
3009	Anterior Commissure	Anti:Synapsin		Optic tract accessory, optic tectum	(not shown)

3010	Post Optic Commissure	Tg(Ath5:GFP)	(Mueller, Wullimann, and Guo 2008)	Optic tract accessory, Preoptic area	(not shown)
3011	Optic Commissure	Tg(Ath5:GFP)		Optic tract accessory	(not shown)
3012	Parapineal	Islet1 HCR, Anti: 5-HT	(Ishikawa et al. 2015; Signore et al. 2009)	Habenula	(not shown)
3013	Nucleus Isthmi	Tg(HuC:DsRed)	(Henriques et al. 2019)	midbrain	(not shown)

Supplementary Table 4. Labeling protocol.

Detail of labeling methods and number of fish used for the experiments are shown. Some of the zebrafish data was cited from a previous study.

Method	Label	Method - detail	Number of fish
Immunostaining			
	tERK	1:250, p44/42 MAPK (Erk1/2) Mouse Ab, 4696S, Cell Signalling Technologies, Lot.L34F12	11
	TH	1:250, AB1532, EMD Millipore Corp, Lot. 3328928	5
	5-HT	1:250, cat.20080, rabbit ab, Immunostar, Lot. 1907001	5
	Synapsin	1:250, Mouse ab, 106011, Synaptic systems, Lot. 1-26	5
	Parvalbumin	1:2000, Mouse ab, P3088-100UL, Sigma-Aldrich, Lot. 0000397786	5 (zebrafish), 6 (medaka)
Transgenic line			
	Ath5:GFP	From the Wittbrodt lab	5
	vglut:GFP	From the Higashijima lab	4
	HuC:DsRed	From NBRP medaka (Okuyama et al. 2013)	11
in situ hybridization			
	Oxt	Molecular Instruments	6
	ChAT	Molecular Instruments	6
	Gad1b	Molecular Instruments	10
	Vglut2 (slc17a6b_vglut2)	Molecular Instruments	10
	snap25	Molecular Instruments	20
	islet-1	Molecular Instruments	4
	hcrt	Molecular Instruments	4
Dyes			
	Texas Red dye	Cat: D3329, Invitrogen	5
	Hoechst	1:1000, Hoechst 33342, #40046 biotum.com	11
	FM1-34	Cat: T3163, Invitrogen	5

Supplementary Table 5. Quantitative comparison of 2D U-net and inter-slice interpolation.

Detailed comparison of both methods across regions by measuring intersection over union (IOU).

Regions	2D U-net IOU	Inter-slice interpolation IOU
Forebrain	94.33	96.41
Midbrain	95.26	96.53
Hindbrain	95.56	96.88
Stratum Periventiculare	91.48	93.84
