# Supplementary material for: A cross-species atlas platform for comparative neuroscience in teleost fish
Source: Nat Commun. 2026 Sep 25;17:11114. doi: 10.1038/s41467-026-77879-1 (PMC13696724; doi:10.1038/s41467-026-77879-1)
Supplement: Supplementary file 2 [file 41467_2026_77879_MOESM2_ESM.pdf]

nature portfolio

Corresponding author(s): Yasuko Isoe

Last updated by author(s): Aug 19, 2026

Reporting Summary

Nature Portfolio wishes to improve the reproducibility of the work that we publish. This form provides structure for consistency and transparency in reporting. For further information on Nature Portfolio policies, see our Editorial Policies and the Editorial Policy Checklist.

Statistics

For all statistical analyses, confirm that the following items are present in the figure legend, table legend, main text, or Methods section.

n/a	Confirmed	
☐	☒	The exact sample size (*n*) for each experimental group/condition, given as a discrete number and unit of measurement
☐	☒	A statement on whether measurements were taken from distinct samples or whether the same sample was measured repeatedly
☐	☒	The statistical test(s) used AND whether they are one- or two-sided<br>*Only common tests should be described solely by name; describe more complex techniques in the Methods section.*
☒	☐	A description of all covariates tested
☒	☐	A description of any assumptions or corrections, such as tests of normality and adjustment for multiple comparisons
☒	☐	A full description of the statistical parameters including central tendency (e.g. means) or other basic estimates (e.g. regression coefficient) AND variation (e.g. standard deviation) or associated estimates of uncertainty (e.g. confidence intervals)
☐	☒	For null hypothesis testing, the test statistic (e.g. *F*, *t*, *r*) with confidence intervals, effect sizes, degrees of freedom and *P* value noted<br>*Give P values as exact values whenever suitable.*
☒	☐	For Bayesian analysis, information on the choice of priors and Markov chain Monte Carlo settings
☒	☐	For hierarchical and complex designs, identification of the appropriate level for tests and full reporting of outcomes
☒	☐	Estimates of effect sizes (e.g. Cohen's *d*, Pearson's *r*), indicating how they were calculated

Our web collection on statistics for biologists contains articles on many of the points above.

Software and code

Policy information about availability of computer code

Data collection	The imaging stacks were taken by using Zeiss microscopy software Zen.
Data analysis	The imaging stacks were analyzed by ANTS and custom-made python codes. A custom-made Fiji plugin is made for making brain region masks, which is available from the following link: https://github.com/zibneuro/fishexplorer/tree/master/NeMO<br>The website atlas platform is available from the following link: https://fishexplorer.zib.de/sandbox/<br>The tutorial is available from the following link: https://fishexplorer.zib.de/sandbox/tutorial/image-registration.html

For manuscripts utilizing custom algorithms or software that are central to the research but not yet described in published literature, software must be made available to editors and reviewers. We strongly encourage code deposition in a community repository (e.g. GitHub). See the Nature Portfolio guidelines for submitting code & software for further information.

Data

Policy information about availability of data

All manuscripts must include a data availability statement. This statement should provide the following information, where applicable:

- Accession codes, unique identifiers, or web links for publicly available datasets
- A description of any restrictions on data availability
- For clinical datasets or third party data, please ensure that the statement adheres to our policy

The FishExplorer platform provides access to medaka reference brain stacks and atalas imaging data, zebrafish reference and atlas imaging data, MECE and MISC masks, gene-expression imaging datasets, and neurotransmitter imaging datasets. The c-fos image data are available through the international open-access repository Dryad (DOI: 10.5061/dryad.zw3r228hw). FishExplorer also provides a tutorial describing the image-registration parameters and workflow, together with the Python script required for registration.

Research involving human participants, their data, or biological material

Policy information about studies with human participants or human data. See also policy information about sex, gender (identity/presentation), and sexual orientation and race, ethnicity and racism.

Reporting on sex and gender	N.A.
Reporting on race, ethnicity, or other socially relevant groupings	N.A.
Population characteristics	N.A.
Recruitment	N.A.
Ethics oversight	N.A.

Note that full information on the approval of the study protocol must also be provided in the manuscript.

Field-specific reporting

Please select the one below that is the best fit for your research. If you are not sure, read the appropriate sections before making your selection.

- [x] Life sciences
- [ ] Behavioural & social sciences
- [ ] Ecological, evolutionary & environmental sciences

For a reference copy of the document with all sections, see nature.com/documents/nr-reporting-summary-flat.pdf

Life sciences study design

All studies must disclose on these points even when the disclosure is negative.

Sample size	No statistical method was used to predetermine sample size. Sample sizes were chosen based on the availability of technically successful biological replicates and the reproducibility of staining and imaging across individuals.
Data exclusions	Staining was always done with either pan-neuronal signal or nucleus staining. Samples with insufficient pan-neuronal or nuclear reference staining for reliable registration or normalization were excluded from quantitative analysis; exclusion was based on reference-signal quality rather than the experimental signal of interest.
Replication	The staining was done with different batches.
Randomization	For the c-fos experiments, samples were randomized across experimental conditions
Blinding	For the c-fos experiments, sample identities were blinded during staining and imaging. Experimental conditions were revealed after image acquisition and before quantitative analysis.

Reporting for specific materials, systems and methods

We require information from authors about some types of materials, experimental systems and methods used in many studies. Here, indicate whether each material, system or method listed is relevant to your study. If you are not sure if a list item applies to your research, read the appropriate section before selecting a response.

Materials & experimental systems

n/a	Involved in the study
☐	☒ Antibodies
☒	☐ Eukaryotic cell lines
☒	☐ Palaeontology and archaeology
☐	☒ Animals and other organisms
☒	☐ Clinical data
☒	☐ Dual use research of concern
☒	☐ Plants

Methods

n/a	Involved in the study
☒	☐ ChIP-seq
☒	☐ Flow cytometry
☒	☐ MRI-based neuroimaging

Antibodies

Antibodies used

anti-tERK p44/42 MAPK (Erk1/2) Mouse Ab, Cat.4696S, 1:250, Cell Signalling Technologies, Lot.L34F12
anti-TH, Cat. AB1532, 1:250, EMD Millipore Corp, Lot. 3328928
anti-5-HT, cat.20080, rabbit ab, 1:250, Immunostar, Lot. 1907001
anti-Synapsin, Mouse ab, Cat. 106011, 1:250, Synaptic systems, Lot 1-26
anti-Parvalbumin, Mouse ab, Cat. P3088-100UL, 1:2000, Sigma-Aldrich, Lot 0000397786

Validation

anti-tERK: https://www.cellsignal.com/products/primary-antibodies/p44-42-mapk-erk1-2-l34f12-mouse-mab/4696?srsltid=AfmBOoqDuFZnpnAk0B-DsnolduyUTz0FIAPwuQVFFO6Smrpfrsfr-nJN
anti-5-HT: https://www.immunostar.com/product/5-ht-serotonin-rabbit-antibody/
anti-Synapsin: https://sysy.com/product/106011
anti-Parvalbumin: https://www.sigmaaldrich.com/US/en/product/sigma/p3088?srsltid=AfmBOoqfL9pRS-bq6dWHQQMu2rmaQ-F7PRhpg6xBOev6G0nQIO1-BYcX

Animals and other research organisms

Policy information about studies involving animals; ARRIVE guidelines recommended for reporting animal research, and Sex and Gender in Research

Laboratory animals

The medaka Tg(vglut:GFP) line was generated in the Higashijima lab and provided by NIBB. The medaka Tg(Ath5:GFP) line was generated by the Wittbrodt lab. The medaka Tg(HuC:loxp-DsRed-loxp-GFP) in ST3 mutant background was generated by crossing medaka Tg(HuC:loxp-DsRed-loxp-GFP) with ST3 mutants. The medaka Tg (ST3-eyesBlack) was established by crossing ST3 mutants and wildtype (drR), then embryos were screened for the absence of autofluorescence around the head, but with pigment cells in the eyes. The zebrafish nacre mutant was used for immunostaining and in situ hybridization. Zebrafish: 6-7 days post fertilization; Medaka fish: 8-9 days post fertilizationwere used.

Wild animals

The study did not involve wild animals.

Reporting on sex

Sex was not considered in the study.

Field-collected samples

The study didn't involve samples collected from the field.

Ethics oversight

All experiments followed institution IACUC protocols as determined by the Harvard University Faculty of Arts and Sciences standing committee on the use of animals in research and teaching. The animal experimentation protocols, 25-03 and 22-04 were submitted and approved by this institution’s animal care and use committee (IACUC). This institution has Animal Welfare Assurances on file with the Office for Laboratory Animal Welfare (OLAW), D16-00358 (A3593-01).

Note that full information on the approval of the study protocol must also be provided in the manuscript.

Plants

Seed stocks

N.A.

Novel plant genotypes

N.A.

Authentication

N.A.
