# Supplementary material for: A cross-species atlas platform for comparative neuroscience in teleost fish
Source: Nat Commun. 2026 Sep 25;17:11114. doi: 10.1038/s41467-026-77879-1 (PMC13696724; doi:10.1038/s41467-026-77879-1)
Supplement: Supplementary file 3 [file 41467_2026_77879_MOESM3_ESM.pdf]

nature portfolio

Peer Review File

A cross-species atlas platform for comparative neuroscience in teleost fish

Corresponding Author: Dr Yasuko Isoe

This file contains all reviewer reports in order by version, followed by all author rebuttals in order by version.

Version 1:

Reviewer comments:

Reviewer #1

(Remarks to the Author)
Reviewer Comments to the Authors
The manuscript presents FishExplorer, an online platform that facilitates data comparisons between medaka and zebrafish. The platform could potentially be expanded to other stages and species. The development of a comparative neuroanatomical platform such as FishExplorer is an exciting and much-needed step forward for the field. The platform represents an important conceptual advance for comparative neuroanatomy and evolutionary studies, offering the potential to analyse brains of different species through a structured approach. The fact that the platform is a public resource that may be extended to additional species, markers, and stages is highly valuable. If fully realized, it could enable broad applications across the neuroscience community. The authors are admirably transparent about the experimental challenges they encountered, such as differences in antibody staining quality and issues in image registration. This openness, while raising concerns, also reflects a realistic assessment of current limitations.
We have several concerns that we believe should be addressed to strengthen the manuscript and the utility of the platform. At present, the platform is somewhat of a work in progress with high potential utility in the future but more limited at present.
1. Platform Functionality
o Analysis tools do not seem available to the user which will discourage use and adoption by the community. Authors should comment on this more openly in the manuscript.
o Registration and brain region mask tools are not in a single pipeline (ImageJ and Python), making it much harder for users to perform these tasks as part of a streamlined process. This will also hamper adoption by the community. Please comment on this in the manuscript.
o There is currently no way for users to create new reference brains within the platform. This limits extending the platform to other species and stages. Please clarify whether such tools will be developed and how. If not, please discuss this as a shortfall of the platform.
2. Species Choice
o There is no consistency in the way the evolutionary distance between zebrafish and medaka is described. In some places, the species are said to be “not too distant,” while elsewhere they are referred to as “phylogenetically distant” (Lines 277-278; also “large evolutionary distance between the two species” in Line 706). Given the phylogenetic distance between these two species is quite large, it seems the choice was primarily based on data availability. If this is the case, it is entirely acceptable. In any case, the justification for selecting these species should be clear and consistent throughout the text.
3. Anatomical Model
o The MECE masks are based on the columnar model, which is considered outdated by most in the field of comparative neuroanatomy (see Mario Wulliman’s and Luis Puelles' literature on the topic). Authors should refer to the neuromeric model and explain why the columnar approach was used. If possible, outline how your system could incorporate neuromeric segmentation in the future. This should not be difficult, as it can be seen using the mapzebrain (diencephalon) as an example.
4. Data Curation
o The manuscript refers to curation of masks based on peer-reviewed literature, but there is no explanation of how this will be managed. Will external experts be able to contribute or edit masks? How will quality control be ensured?
5. Neuroanatomical Validation
o It is not clear in the text which markers were used for medaka. We think this is in Table 4, but we are not completely sure. Authors should show clearly which markers were used for medaka only.
o The identification of pallial subdivisions in the telencephalon, other than Dp, seems speculative. The location of Dp has

been clearly shown in zebrafish larva (Miyasaka et al., 2014) and agrees with what is shown in the manuscript. However, without appropriate markers (gene expression, connections, etc.) other subdivisions, such as Dm, Dc and Dl, cannot be reliably annotated. Please provide additional support for the pallial subdivisions provided and/or soften the claims.
6. Quantitative Comparisons
o While the manuscript suggests that cross-species comparisons can be done within FishExplorer, we understand that tools for measurements are not yet available. Please clarify what types of analysis can be done through the platform and what must be done offline.
o How was quantitative data on signal intensity and size of brain region extracted from the Fishexplorer platform? This needs to be included in the methods.
o We understand that the platform does not generate the Manhattan Plots. Based on the text, it seems this sort of analysis will be easy once data has been registered to the reference brains or generated by the platform. Please clarify this point.
7. Experimental Limitations
o The issues faced by the authors themselves in staining, imaging, registering and analysing data are a real concern for potential users of this platform. Authors point at issues with the data, such us not very intense staining for ventral cells in the brain, and complications in the normalisation and image analysis procedures (Lines 388- 404). Authors highlight differences in 5HT expression (Lines 325-328) but acknowledge they may stem from staining variability between the two species. Authors point again the issue that differences between specimens may reflect experimental variability rather than true biological differences, is highlighted in lines 427-435. How can users overcome these issues? How can users control for or correct discrepancies? Consider providing guidance or tools to assist with normalization.
o The methods section should describe how signal intensity and region size were quantified, particularly as this is central to the results (e.g., Manhattan plots). Also explain how the fluorescent expression was normalized.
Minor Points
o Line 42: should say brain, not cerebral. And maybe better to say nuclei, rather than subregions.
o Lines 52-63: We agree that this is a good proposal to analyse in detail brain evolution and diversity. However, as it is written now, it seems that the platform provides tools for performing all these steps, which is not the case. Hence, authors should rephrase this paragraph for clarity.
o Line 66–67: The phrase “allow for a quantitative and direct cross-species comparison” is misleading if the platform itself does not provide the tools needed for such analyses.
o Lines 70-78: Authors could add another reason why teleosts are such a great group for studies of brain evolution, and that is the large number of species (more than 30,000) adapted to very different ecological niches. This provides the opportunity to study closely related species that are adapted to different environments or that display slightly different behaviours or brain modifications (see Ito et al., 2007).
o Lines 139-163 description of how IDs are decided is currently in Results, figure legend and methods. Could be briefly included in Results and, a more detailed version, in methods. The visual on this in the figures is ok, so potentially think about how to make this standalone.
o Line 194: The X-ray dataset does not appear to load. Please clarify its purpose and how it is used.
o Line 492: in Figure 5d, why do different scales for zebrafish and medaka are used in y-axis (while the same scale is used in f)?
o Line 523–524: The shaker assay needs further justification. It appears to be a general stress test rather than a specific sensory behaviour assay. Consider referencing or describing its validation.
o Line 545–550: The use of MS-222 requires clearer explanation regarding dosage. Also, could it have potential effects on stress circuits? Could confound behavioral or anatomical results?
o Lines 693-698: seems very speculative.
o Lines 723-725: authors note that Dd has been identified in adult medaka but not in larval zebrafish. This is true, but there is plenty of zebrafish literature describing Dd in the adult zebrafish (e.g. Yañez et al., 2022). Consider incorporating reference to Dd in the adult zebrafish.
o Line 986: Feeding assay is listed in Methods but not discussed in the main text or figures.
o Tables and Figures: Some table legends are missing (or I could not find them). Table 1’s final column is misaligned. Clarify what “HCR” stands for in Table 4.
o Table 1: Authors talk about “marker-gene expression pattern”, but this does not seem the right term, as they used gene markers but also neurotransmitters, synaptic vesicles, etc. So “expression patterns” seems better term. I could not find a table legend.
o Supplementary Figure 1 iv: Try to include this figure in the main text. It is crucial to show that the MECE masks have been carefully done based on detailed neuronatomical analysis.

(Remarks on code availability)

Reviewer #2

(Remarks to the Author)
I co-reviewed this manuscript with one of the reviewers who provided the listed reports. This is part of the Nature Communications initiative to facilitate training in peer review and to provide appropriate recognition for Early Career Researchers who co-review manuscripts.

(Remarks on code availability)

Reviewer #3

(Remarks to the Author)
I co-reviewed this manuscript with one of the reviewers who provided the listed reports. This is part of the Nature Communications initiative to facilitate training in peer review and to provide appropriate recognition for Early Career Researchers who co-review manuscripts.

(Remarks on code availability)

Reviewer #4

(Remarks to the Author)
Vohra et al. present a multimodal brain atlas platform for larval medaka that is integrated with the previously established and widely used zebrafish Z Brain atlas. To construct the medaka atlas, the authors developed a manual segmentation tool and a machine-learning–assisted segmentation pipeline. They created a hierarchical nomenclature for the segmentation masks and then used them to compare anatomical features of the brains of both teleosts. Furthermore, they compared brain activity elicited by different stimuli using an immediate early gene approach.

The resources and tools described here will be highly valuable to the community, especially the integrated platform that could facilitate comparative cross-species studies and serve as a pool for anatomy and molecular and functional data. I recommend this paper for publication, provided that the following issues can be addressed in a revised version:

Major issues:

Definition and homology of brain regions
Cross-species comparisons of anatomy, molecular markers, or functional activity require a rigorous, transparent definition of each region, and a clear rationale for how homology was established. For example, Figure 6 reports greater activation of the pallial Dm and Dd in medaka than in zebrafish following social stimuli. Given the evolutionary divergence and resulting telencephalic rearrangements in these species, it is unclear how Dm and Dd were delineated, and why they are considered homologous. If these assignments rely on published molecular markers or gene-expression patterns, this should be explicitly referenced. Likewise, the suggestion in line 438 that serotonergic signaling might be more prevalent in zebrafish seems premature without stronger evidence.
The authors do provide a table summarizing how individual regions were annotated. However, this table is split across multiple pages, making it unfortunately difficult to parse. Moreover, the descriptions it contains are relatively brief and don’t fully clarify the criteria that were used to assign label identities. This is of particular importance for key regions that the authors claim show differences between the species, such as Dm and Dd, and are therefore central to the paper’s conclusions. For such comparisons to have explanatory value, the authors should include the data that were used to identify these regions in each species (anatomical, molecular, or functional). Otherwise, the observed differences may reflect differences in anatomical labeling itself rather that a true biological divergence. Given that cross-species comparisons are a central aim of this paper, the challenge of defining and mapping homologous brain regions across species should be discussed explicitly. If regional labels were assigned primarily by expert judgement, that limitation should be acknowledged and its impact on the conclusions carefully considered. The authors mention that many brain regions are defined using expert judgment and refer to a supplementary table summarizing this, but this is presented as a practical matter, not discussed as a limitation or scientific challenge.

Details of the machine-learning segmentation pipeline
To further address the issue of the anatomical comparisons, the authors developed a machine learning-assisted brain segmentation pipeline. However, the entire description of this process appears to be incomplete. How was the training data created and augmented? Was this done only on the high-level anatomical areas or further extended to the individual brain areas? Lines 899 and 814: what is meant by segmenting 20% of brain areas? Is it 20% of slices or is it 20% of regions? If the former, then the authors should discuss how the output compares to the conventional morphological inter-slice interpolation.

Statistical treatment of cross-species comparisons
All cross-species comparisons in this paper, except for negative control comparisons in Figs. 5b and 6c, are presented as comparisons of two mean values between the reference brains of medaka and zebrafish, without reporting the sample variability or conducting any kind of significance testing. This leaves open the possibility that some of the reported differences reflect random variation. Please report the N numbers in the figure legends, including measures of variability across fish, and perform statistical tests (e.g., ANOVA with post-hoc comparisons) to support the claims. One area where this is important is the comparisons of the functional activity with c-Fos. It is known that c-Fos expression following a stimulus can be quite variable, and making comparisons based on the mean normalized c-Fos mRNA expression for each condition could mask these differences. The authors should include the individual staining used for these comparisons.

Figures:
General: Several figures include screen captures with very small, often unreadable text. Even the high-resolution figures at the end are not legible when printed. Please consider increasing font size or labeling clarity. The same goes for brain region labels (e.g. Figure 2d).
Figure 1: please adjust the positioning of the panels to clearly separate them.

Figures 1i and S1-vi a: Label (iv) appears to be missing.
Figure 1e: blue-orange color-code has overlapping meanings: it corresponds to both zebrafish-medaka and hindbrain-midbrain dichotomy, which may be confusing. Please consider using a different pair of colors for on of the dichotomies.
Figure S1-iii: Please define how "thickness" was computed (number of slices?), specify units in panel d, and clarify what distinguishes panels c and e.
Figure 2d: Please explain R1–R7 in the legend (rhombomeres).
Figure 5 and line 523: Why did the authors choose 60 minutes as the shaker stimulus duration for c-Fos expression cut-off if multiple studies indicate peak expression around 30 min? Habituation effects could arise that when combined with the c-Fos degradation would decrease the detected signal.
Figure 5 and line 541: MS-222 experiments: how long were fish in MS-222? Was the concentration calibrated to render fish non-reactive to stimuli? In case, like the authors described, of potentially insufficient anesthetic effect on medaka, how could one compare the activity between the two fish if one is not anesthetized completely and other is?
Figures 5 and 6: add scale bars and, more importantly, a color bar for the delta c-Fos plots. The negative controls are also presented in grayscale, while the difference is in a colormap.

Minor issues:
— Line 74: "easy extrapolation": Please consider removing the word "easy" or rephrasing more cautiously
— Lines 129-131 / Fig. 2: It seems like there are only two levels of hierarchy created in the brain regions: macro- and mini-masks. Later (Line 259) it seems that the mini-masks have their own hierarchy. Furthermore, in the atlas viewer itself there are more than two levels. Please clarify to avoid confusion.
— Lines 151 and 965: typo "vasculatore"
— Lines 235-237: The color-coding of the diagram is only partially explained in the legend. Also, why is "prepare external user data" not shown in blue?
— Line 322: what is meant by normalizing in this context? Please add an explanation.
— Line 327: whole tissue stainings in general have a high autofluorescence problem. If a claim can't be made because of these artefacts, consider moving speculation to discussion or adding staining data to allow the reader to assess the differences themselves.
— Lines 341-466: This section is lengthy and includes highly interpretive content that would fit better in the Discussion. Please consider streamlining the Results and moving speculative elements to the Discussion.
— Line 492: consider using the word "independent" instead of "orthogonal" to avoid confusion.
— Line 698: consider providing references to support the claim about zebrafish evolving in murky water.

(Remarks on code availability)

Reviewer #5

(Remarks to the Author)
Vohra et al describe a cross species platform for the analysis of brains in teleosts. The manuscript is excellent and the website and other resources promise to considerably further the field of comparative neuroanatomy. Overall, this is an excellent piece of work. I mostly have suggestions for clarification and improvements. The biggest suggested improvement I have is the incorporation of measures of variation and statistical tests to help the reader better understand the size of the effects, their reliability, and their variation.

I would also like to applaud the discussion of the challenges associated with the imaging and staining techniques used in the paper (lines 388-404; 426-435). These sorts of candid discussions are usually missing in papers. It is refreshing to see some of the technical challenges associated with this type of work made explicit. This is particularly helpful for trainees so that they have a better understanding of challenges and limitations.

Major points
While the presentation of the data using step plots through the paper is clear and intuitive, they lack a measurement of variation across animals (i.e., error bars or ribbons). With respect to the cfos data, it is stated (line 480) that variance between individuals is 'low'. But what does that mean? Indeed, if I'm understanding the data correctly, it appears that there is a significant amount of variation between individuals in some experiments (e.g., Figure 5d-1-3). It is not clear how much variation there is in other experiments in the paper (e.g., figures 2-4, 6).

It is also not clear how 'significance' is determined aside from figure 5 that uses z-scores. For example, on line 272 it's stated that the hindbrain is larger in zebrafish than medaka. However, as best I can tell no statistics were used to make this comparison, and there are no measurements of effect sizes, and no corrections for multiple comparisons. Thus, it is difficult for the reader to assess how large, reliable or variable the findings are.

Minor points
Line 82. A sentence or two more clearly defining the different natural environments of medaka and zebrafish (beyond just higher tolerance to salinity) would be helpful for the reader to better appreciate the common and distinct aspects of these species.

Line 86. The reference for Ishikawa et al has 'n.d.' for the date. This should be updated.
The number of animals used for each experiment should be included in each of the figure captions.

Line 269 and figure captions. The plots would be better described as ‘step plots’ not ‘Manhattan plots’.

Line 556. There is a stray ‘(‘.

Line 692. This point needs a citation.

While I appreciate the challenge of ‘age matching’ zebrafish and medaka, the discussion should include the point that some of the differences observed may be due to different rates of development of different parts of the brain. There is no guarantee that all parts of the brain or body develop at the same rate in zebrafish and medaka (or any two species for that matter) and thus that there may be no such timepoint where everything is ‘matched’. This is an important caveat for this type of developmental comparative work in general.

Related to the above point, I would be interested to see a small expansion of the discussion as to how adult and juvenile brains may be incorporated into this resource. Being mature, adult brains may provide a more stable basis for comparative work. How would your resource handle the drastically different sizes, number of brain regions, and need to use different histological techniques like tissue clearing that can affect brain size or morphology.

The website is well designed and easy to use. My only suggestion would be to consider adding a functionality that allows the user to directly compare images from different species. An option for overlays would be great, but I don’t know how feasible that would be since different species wouldn’t necessarily be in the same coordinate space. However, an option for side-by-side viewing would make comparative analysis easier instead of needing to switch between images. As best I could tell, this functionality was not there.

(Remarks on code availability)
The website appears useable. I did not try uploading new data to it however.

Version 2:

Reviewer comments:

Reviewer #1

(Remarks to the Author)
Further Reviewer 1 Comments to Author following rebuttal:

(3) There is currently no way for users to create new reference brains within the platform. This limits extending the platform to other species and stages. Please clarify whether such tools will be developed and how. If not, please discuss this as a shortfall of the platform.
> Indeed, the current platform is not compatible with - nor intended for - the ingestion and analysis of species other than zebrafish and medaka. In its current form, it lacks the functionality for cross-developmental analysis

There are still references in the paper that do make it sound more like that is what they envisage it being used for, some examples:
Line 100-101 The platform’s database can be expanded in many ways, e.g. in terms of tissue types, biological species and developmental stages.
Line 112 multi-species atlas
Line132 if we include more species
So we think the authors need a clearer statement on this point.

(8) The identification of pallial subdivisions in the telencephalon, other than Dp, seems speculative. The location of Dp has been clearly shown in zebrafish larva (Miyasaka et al., 2014) and agrees with what is shown in the manuscript. However, without appropriate markers (gene expression, connections, etc.) other subdivisions, such as Dm, Dc and Dl, cannot be reliably annotated. Please provide additional support for the pallial subdivisions provided and/or soften the claims.
> Thank you! We now provide additional support for the pallial subdivisions with DAPI staining and other stainings in Figure 6a.

We think that the authors’ response does not address the core of the concern, although we appreciate their effort in adding new analyses. The use of DAPI (and HuC) and the other general stainings does not provide sufficient evidence for the identity of pallial subdivisions. The additional markers used by the authors (GFAP, vglut2a, synapsin and HuC) provide valuable information on the general organization of the telencephalon; however, they are not specific to pallial domains and therefore do not constitute evidence for their identity. None of these markers can discriminate between Dm, Dc and Dl. As stated in the original comment, without appropriate markers (e.g. domain-specific gene expression and/or tract-tracing data), the annotation of Dm, Dc and Dl remains speculative. The authors should therefore either provide markers supporting the identity of these pallial domains or explicitly soften the terminology throughout the manuscript (e.g. “putative Dm/Dc/Dl based

on topography") and clearly state that these assignments are tentative in the Results and Discussion. In its current form, Figure 7 and the associated text still imply a level of anatomical certainty that is not supported by the data.

Another issue concerns the discussion of the dorsal pallium (Dd). The adult medaka study cited (Isoe et al.) convincingly demonstrates the existence of an epigenetically and clonally distinct dorsal pallial compartment in medaka. However, the statement that such a region "has not been confirmed to exist in zebrafish larvae" may be misleading. The absence of evidence in zebrafish does not necessarily imply the absence of a homologous structure. Rather, it may reflect methodological limitations, as a detailed study of the larval zebrafish pallium comparable to that in medaka is still missing, as well as unresolved issues of nomenclature and homology in the teleost telencephalon.
A dorsal pallial domain (Dd) has been described in adult zebrafish-based connectivity (Yáñez et al., 2022), and in multiple other teleost species (Braford et al., 2009; Burmeister et al., 2009; Demski et al., 2013). Therefore, from a comparative and evolutionary perspective, it seems plausible a Dd-like domain may also be present in zebrafish, including at larval stages, but has not yet been adequately characterized. Accordingly, the current lack of evidence in zebrafish larvae should be interpreted with caution, and the wording in the manuscript should reflect this uncertainty.

>>Note: This data is in Fig 7 not Fig 6a. Delineated areas are very difficult to see in the transerve sections, so I perhaps make these images bigger.

Minor points associated with this comment
(a) Reference needs updating. At the moment, anatomical delineation of most pallial areas seem to be based on a preprint:

1260-1261 33. Isoe, Y. et al. Epigenetically distinct synaptic architecture in clonal compartments in the teleostean dorsal pallium. Preprint at https://doi.org/10.1101/2022.10.01.510385.

Reference should be updated, as doi doesn't work and it is now published https://elifesciences.org/articles/85093

(b) Line 619-620 "defined by the anatomical 619 boundaries labeled by
transgenic lines"

No transgenic lines or any other markers are listed in the Table for telencephalon for the pallial areas apart from synapsin staining and references Isoe et al, 2023 (see separate note about updating this reference).

(C) Line 592-593 Labeling synapse density using a presynaptic marker, synapsin, which is ubiquitously expressed in all vertebrates, suggests different compartment patterns of computation in the telencephalon between the two species

We don't see synapsin as an available label on the Fishexplorer atlas and needs more explanation on how that marker would differentiate between subdomains.

Minor Comments on additional text

Additional text Lines 753-756

Lines755-756 but we also share the recipes and protocols for registration to reference brains, and mask generation for other species and developmental stages (see 'Cross-species analysis' tutorial in the FishExplorer).

We couldn't initially find this as it is labelled "cross-section analysis" on the tutorial webpage list.

Notes on the utility of the platform itself

Having tried the explorer on several different computers, it is still not problematic for visualizing expression patterns. The images used throughout the paper showing maximum projections of zstacks are not viewable using the viewer - in 3D view it is still a horizontal slice not really 3D. It should be made clear that these images are not from the viewer but from raw data.

(1) Analysis tools do not seem available to the user which will discourage use and adoption by
the community. Authors should comment on this more openly in the manuscript.
> We agree that this was a serious omission. We have now added a dedicated analysis section
to the platform where users can generate Manhattan plots in various configurations. (Please
have a look at the FishExplorer and Comparative analysis tutorial
(https://fishexplorer.zib.de/sandbox/)).

The analysis tool doesn't allow us to download anything after comparing expression patterns for different brain regions. The ideas are good, but the implementation is still problematic.

(Remarks on code availability)

Reviewer #2

(Remarks to the Author)

I co-reviewed this manuscript with one of the reviewers who provided the listed reports. This is part of the Nature Communications initiative to facilitate training in peer review and to provide appropriate recognition for Early Career Researchers who co-review manuscripts.

(Remarks on code availability)

Reviewer #3

(Remarks to the Author)
I co-reviewed this manuscript with one of the reviewers who provided the listed reports. This is part of the Nature Communications initiative to facilitate training in peer review and to provide appropriate recognition for Early Career Researchers who co-review manuscripts.

(Remarks on code availability)

Reviewer #4

(Remarks to the Author)
The authors addressed the majority of our comments in the response, and we appreciate the effort they put into the revision. That said, we find that several points are not yet fully addressed in the revised manuscript. We ask the authors for the following minor, but necessary changes:

>> (29) Definition and homology of brain regions

The text addresses our concerns; however, please include high-resolution versions of the synapsin/H2B:GFP magenta/green overlays in Fig. 7b. In the current figure, the resolution is not sufficient to clearly evaluate the overlap between synapsin antibody staining and the Dd mask.

>> (30) Likewise, the suggestion in line 438 that serotonergic signaling might be more prevalent in zebrafish seems premature without stronger evidence.
> We agree. We now discuss explicitly that these differences might be due to staining issues
(page 34, line 715-720).

The Discussion now explains the challenges of cross-dataset normalisation more adequately, which is helpful. However, the Results text in the lines 436-443 –

"...suggesting that serotonergic signalling in motor modulation may be more prevalent in zebrafish."; "Perhaps the modulation of visual signals is under tighter dopaminergic control in this species."; …, suggesting a possibly enhanced role of glial processing, which is known to be under the influence of noradrenergic inputs from nearby hindbrain regions."

… still needs to be revised to match this. As written, the Results section continues to make claims about differential serotonergic, dopaminergic, and noradrenergic signalling, but these are later qualified (and potentially undermined) in the Discussion due to the risk of misinterpretation. In our view, such claims should not be presented as part of the findings/Results, especially if they are subsequently framed as potentially unreliable.

Please retain the quantification in the Results, but move any speculative or interpretive statements to the Discussion, where the limitations can be appropriately addressed alongside them.

>> Lines 899 and 814: what is meant by segmenting 20% of brain areas? Is it 20% of slices or is it 20% of regions? If the former, then the authors should discuss how the output compares to the conventional morphological inter-slice interpolation.
> It is the former. We find that the classifier provides slightly smoother transitions than a
conventional interpolation. However, we don't think the difference is critical. We now state and
discuss this in the Methods section (page 42, line 906-907).

The revised lines 906-907 – "From the 9 fully labelled binary-segmented brain regions, we generated training data with 20%, 25%, 30% and 35% of slices using the deep learning panel of the Fiji plug-in, NeMO" – do not address the second question about the comparison with the morphological inter-slice interpolation.

>> (38) Figure S1-iii: Please define how "thickness" was computed (number of slices?), specify
units in panel d, and clarify what distinguishes panels c and e.
> The thickness is indeed computed as the number of slices, which we now specify in the table. We have combined a, c, d and e into a unified table and panel.

The thickness is still not defined in the (now) Figure S2-iiid panel or its caption, and the difference between panels c and e has not been clarified.
Please indicate thickness in defined units (micrometers) and modify the caption to: “List of the remaining 13 regions and their respective thicknesses in micrometers.”
In addition, please revise the captions as follows:
Panel c: “…for each of the initially annotated 8 regions using the selected mode.”
Panel e: “…for each of the remaining 13 regions annotated.”

>> (41) Figure 5 and line 541: MS-222 experiments: how long were fish in MS-222? Was the concentration calibrated to render fish non-reactive to stimuli? In case, like the authors described, of potentially insufficient anesthetic effect on medaka, how could one compare the activity between the two fish if one is not anesthetized completely and other is?
> This is a valid concern. However, it is non-trivial to rigorously assess depth of anesthesia in a larval zebrafish, beyond careful monitoring its swimming activity. For a clean comparison across species we matched the protocols. All fish were immersed in 160 micrograms per milliliter MS-222 (standard dose) for 60 minutes. We found that generally as little as 30 seconds of exposure is sufficient to completely anesthetize a 7dpf larval zebrafish (reference: Leyden et al. 2022; Barabási et al. 2024). For Medaka we used the same protocol because they match in size and the general physiology is similar. We further confirmed that medaka didn’t show any movements in this condition. A more explicit test for anesthesia in medaka would require concurrent brain wide imaging, an approach that is beyond the scope of this study. As such, the depth of anesthesia in medaka is not confirmed. We now state this limitation in the text (page 35, line 731-732).

The revised lines 731-732 in the Discussion “ In medaka, we found activation in different areas of the brain, which might indicate a different chemical sensation than in zebrafish.” do *not* state or discuss any limitations and the Results contain several references to “general” or “full” anaesthesia.

We ask the authors to remove references to “under general anaesthesia”, “full anaesthesia”, interpretations (“puzzling”) and speculative statements (neuromasts as chemical sensors) from the Results. Additionally, please correct the tautological phrasing in lines 730-731 (the “consistent with previous findings” statement appears twice in the same sentence) and discuss limitations of comparing anaesthesia levels across species.

Please also check the MS-222 concentrations in the revised manuscript; you state you used the standard dose of 160 ug/mL (matching the literature) but the manuscript states 60 ug/mL multiple times (line 472, line 1005).

(Remarks on code availability)

Reviewer #5

(Remarks to the Author)
The author's have done a great job responding to reviewer comments and updating their paper and website. They have addressed all of my concerns; however, it looks like there are no error bars on some of the images, e.g., Figures 4 and 5 for 5-HT, TH, and Oxt.

(Remarks on code availability)

Version 3:

Reviewer comments:

Reviewer #1

(Remarks to the Author)
Thanks to the authors for their additional revisions. We are happy with the manuscript to be published in its current state but do have some suggestions for the authors to consider. These primarily relate to the pallial subdomain comparative section. In the light of the new results, we still have some concerns about figure 7 and the related sections:

Authors comment>>> Thank you. Now we performed an anti-parvalbumin immunohistochemistry experiment, inspired by aprevious report on labeling the pallium subdivisions in adult zebrafish (Mueller T et al. 2011). In this study, parvalbumin-positive cells are observed in Dl and Dc in the adult brain. In our new experiment with larvae, as shown in the new version of Figure 7 and Supplementary Figure 7, we detected very strong signals in the olfactory bulb and olfactory epithelium. We also observed an parvalbumin expression in the pallium as well as signals in cell bodies of Dl and axon fibers of the Dc. This result aligns with the staining result in adult zebrafish (Mueller T. et al. 2011). We confirm that the signal boundaries are consistent with the current MISC masks of larval zebrafish. (Figure 7: pages 19-20, lines 611-618)

The authors appropriately selected a marker (parvalbumin-PV) based on the adult literature to visualize Dl and Dc in the larva. However, they only apply it in zebrafish. Given that the manuscript compares two species, it would be desirable to

analyse PV in medaka as well.

Regarding PV expression, the data does not appear align very well with the zebrafish masks,. Supplementary Figure 7a shows PV expression located rather caudally, with a relatively large gap between the PV+ expression in the Dl and olfactory bulb. Based on this PV data, the authors have refined labels in synapsin expression (fig. 7c), but not the pallial masks. For instance, Dl mask extends much more than the Dl PV+ area (comparison between Supplementary figure7a and Fig. 7b). These mis-matches weaken confidence in the proposed anatomical subdivisions. A single marker is sometimes insufficient to define pallial boundaries and we suggest that the authors should explicitly soften the terminology throughout the manuscript (e.g., “putative Dm/Dc/Dl based on topography”) and clearly state in both the Results and Discussion that pallial masks are tentative.

>>> “we found that medaka display stronger synapsin-positive signals in the dorsal area of the pallium (Dd, ID: 3005), which is consistent with the expression pattern in the adult pallium (Mueller T et al. 2011) (Supplementary Fig. 7”

In what sense the reported expression pattern is consistent with Mueller et al. as that study did not analyze synapsin or any other synaptic marker and indeed, according to Mueller et al., a distinct Dd subdivision is absent in zebrafish.

>>> We also added the imaging stacks in FishExplorer.
We couldnt find this data in FishExplorer please check if anti-Parvalbumin and anti-synapsin date are present.

Thank you for your insightful and careful comments! We have increased the size of the images. We
concur that greater care should be exercised when describing the dorsal pallium in larval zebrafish. We have expanded the Discussion Section to include descriptions of the potential Dd region in larval zebrafish as follows: “Also the connectivity study in adult zebrafish across the compartments in the pallium and other brain regions support the idea of existence of a unique anatomical compartment in the dorsal pallium (Yáñez et al., 2022). In addition, previous works in multiple other teleost species strongly suggest the dorsal pallium is conserved across teleost (Braford et al., 2009; Burmeister et al., 2009; Demski et al., 2013). Therefore, from a comparative and evolutionary perspective, it seems plausible that a Dd-like domain is present in zebrafish, including at larval stages, however it needs more careful characterization.” (page 23, lines 757-750).

The authors may want to comment on how the possibility that Dd exists in zebrafish affects the interpretation of their results for the telencephalon and the comparison between zebrafish and medaka.

(Remarks on code availability)

Reviewer #2

(Remarks to the Author)
I co-reviewed this manuscript with one of the reviewers who provided the listed reports. This is part of the Nature Communications initiative to facilitate training in peer review and to provide appropriate recognition for Early Career Researchers who co-review manuscripts.

(Remarks on code availability)

Reviewer #3

(Remarks to the Author)
I co-reviewed this manuscript with one of the reviewers who provided the listed reports. This is part of the Nature Communications initiative to facilitate training in peer review and to provide appropriate recognition for Early Career Researchers who co-review manuscripts.

(Remarks on code availability)

Reviewer #4

(Remarks to the Author)
Thank you for the revision. Our comments were addressed.

(Remarks on code availability)

Open Access This Peer Review File is licensed under a Creative Commons Attribution 4.0 International License, which permits use, sharing, adaptation, distribution and reproduction in any medium or format, as long as you give appropriate credit to the original author(s) and the source, provide a link to the Creative Commons license, and indicate if changes were made.
In cases where reviewers are anonymous, credit should be given to 'Anonymous Referee' and the source.
The images or other third party material in this Peer Review File are included in the article's Creative Commons license, unless indicated otherwise in a credit line to the material. If material is not included in the article's Creative Commons license and your intended use is not permitted by statutory regulation or exceeds the permitted use, you will need to obtain permission directly from the copyright holder.
To view a copy of this license, visit https://creativecommons.org/licenses/by/4.0/

REVIEWER COMMENTS

We appreciate all the comments from the reviewers. Here we wrote comments in blue letters, and the corresponding changes are highlighted in yellow and red in "manuscript_highlighted_revised_version.pdf". The page number and Line number in this document correspond to those in "manuscript_highlighted_revised_version.pdf".

Reviewer #1 (Remarks to the Author):

Reviewer Comments to the Authors
The manuscript presents FishExplorer, an online platform that facilitates data comparisons between medaka and zebrafish. The platform could potentially be expanded to other stages and species. The development of a comparative neuroanatomical platform such as FishExplorer is an exciting and much-needed step forward for the field. The platform represents an important conceptual advance for comparative neuroanatomy and evolutionary studies, offering the potential to analyse brains of different species through a structured approach. The fact that the platform is a public resource that may be extended to additional species, markers, and stages is highly valuable. If fully realized, it could enable broad applications across the neuroscience community. The authors are admirably transparent about the experimental challenges they encountered, such as differences in antibody staining quality and issues in image registration. This openness, while raising concerns, also reflects a realistic assessment of current limitations.

We have several concerns that we believe should be addressed to strengthen the manuscript and the utility of the platform. At present, the platform is somewhat of a work in progress with high potential utility in the future but more limited at present.

1. Platform Functionality
(1) Analysis tools do not seem available to the user which will discourage use and adoption by the community. Authors should comment on this more openly in the manuscript.

> We agree that this was a serious omission. We have now added a dedicated analysis section to the platform where users can generate Manhattan plots in various configurations. (Please have a look at the FishExplorer and Comparative analysis tutorial (https://fishexplorer.zib.de/sandbox/)).

(2) Registration and brain region mask tools are not in a single pipeline (ImageJ and Python), making it much harder for users to perform these tasks as part of a streamlined process. This will also hamper adoption by the community. Please comment on this in the manuscript.

> We agree that we hadn't been clear enough in the manuscript about the exact nature of the services we offer, and of how we envision the platform to be used. We have now clarified this and also integrated registration functionality into the platform. (Please have a look at FishExplorer (https://fishexplorer.zib.de/sandbox/)).

The core and integral service we offer and the main benefit to the community relates to visualization and depository services. Registration to reference brains, mask generation and in-depth analysis of the data are services we did not plan to offer and support, but rather recipes and protocols we publish and share (relying on well-developed and well-documented open source software).
However, given the feedback we received, we have decided to include registration into the service package - albeit to a limited extent. The preferred operation continues to be for users to complete registration in house and then upload the registered stacks. We are committed to helping and advising if there are problems and obstacles, but we will not offer registration as a routine and default service.

“Mask generation”, however, differs significantly from “stack registration” and the two processes are not directly related to each other. One process can be performed completely independently of the other. While registration is a required process for integrating a user’s volume into the Atlas, mask generation is not.
We believe that the proposed solution using ImageJ and a Python command-line approach for registration is an appropriate solution and that running these to operations on separated platforms does not diminish user-friendliness.
We have clarified this in the manuscript, and we have improved and augmented the tutorials to make the user’s job easier.

To streamline the mask generation process, we have integrated the registration script as an ImageJ plugin. In addition, we created a step-by-step tutorial that explains to users how to install ANTs, set up the parameters, and run the script via the ImageJ plugin.
We have further streamlined the whole process and made all elements available for download on GitHub. The process still consists of separate elements, including ImageJ (for the mask tool), ANTS for registration, and Python for analysis. The Github package should now make it easier for users to coordinate these various elements and integrate them into their workflow.
We have also updated the Tutorials; the text is now much clearer (please have a look in FishExplorer).

We have also integrated the registration functionality into the Fishexplorer platform using our standard registration script. Users can register up to 10 datasets per month. Once the registration process of their custom dataset is completed, users will be notified via email. A detailed tutorial regarding online registration is also provided, along with the necessary details.

(3) There is currently no way for users to create new reference brains within the platform. This limits extending the platform to other species and stages. Please clarify whether such tools will be developed and how. If not, please discuss this as a shortfall of the platform.

> Indeed, the current platform is not compatible with - nor intended for - the ingestion and analysis of species other than zebrafish and medaka. In its current form, it lacks the functionality for cross-developmental analysis.

We anticipate that the next generation of our platform will be able to accommodate such collaborative efforts.
Users, who would like to create and upload a new reference brain of a different species or developmental stage are encouraged to contact the corresponding author and join our efforts for the next iteration.
We have now added an explanation in the discussion that lays out our concrete plans more clearly (page 33, line 687-692).
If the desire is to create and integrate alternative reference brains for 1 week old medaka or zebrafish, then this is very straightforward. You can just use the existing infrastructure to upload stacks, register them into our coordinates, and call that stack “Reference Brain”, and use it accordingly.

2. Species Choice
(4) There is no consistency in the way the evolutionary distance between zebrafish and medaka is described. In some places, the species are said to be “not too distant,” while elsewhere they are referred to as “phylogenetically distant” (Lines 277-278; also “large evolutionary distance between the two species” in Line 706). Given the phylogenetic distance between these two species is quite large, it seems the choice was primarily based on data availability. If this is the case, it is entirely acceptable. In any case, the justification for selecting these species should be clear and consistent throughout the text.

> Thank you very much for making this point! It is very helpful.
We now consistently refer to medaka and zebrafish as “phylogenetically distant” (page 4, line 73-74; page 13, line 271).

3. Anatomical Model
(5) The MECE masks are based on the columnar model, which is considered outdated by most in the field of comparative neuroanatomy (see Mario Wulliman’s and Luis Puelles' literature on the topic). Authors should refer to the neuromeric model and explain why the columnar approach was used. If possible, outline how your system could incorporate neuromeric segmentation in the future. This should not be difficult, as it can be seen using the mapzebrain (diencephalon) as an example.

> We acknowledge that there is generally a tension between defining MECE masks based on anatomy and function on the one hand (columnar), and based on gene expression and developmental trajectories on the other (neuromeric).
Here, we tried to compromise between those two approaches and may have erred too much on the side of a columnar representation.
We now mention this explicitly in the manuscript and also highlight the explicit examples where we follow the more modern neuromeric approach (page 32, line 645-651).
In the discussion we now highlight the respective advantages of the two orthogonal approaches and we point the reader who is more focused on development and transcription to MAPZebrain as an alternative resource (page 33, line 652-656).

In the discussion, we also point out that having complementary approaches - and a variety of different platforms that prioritize different aspects - are of use to a diverse population of potential users (page 33, line 675-677).

4. Data Curation

(6) The manuscript refers to curation of masks based on peer-reviewed literature, but there is no explanation of how this will be managed. Will external experts be able to contribute or edit masks? How will quality control be ensured?

> This is an excellent point. We realize that we have not been clear enough about this process.
We have clarified in the text that there is a clear distinction between MISC masks and MECE masks.
The process for MISC masks does not require much curation and quality control beyond publication in a peer-reviewed journal. Once this has happened, all newly published MISC masks are automatically transferred to the publicly accessible part of the FishExplorer.
MECE masks are not subject to routine update by the community. Curation and updating of MECE regions will require a much more substantial reorganization of the database and will likely have to involve a 'consortium' of experts. This process should be accompanied by a conference proceeding or a publication.
We have now added this language to the discussion and Supplementary Figure 2-vi**.** (page 33, line 687-692).

5. Neuroanatomical Validation

(7) It is not clear in the text which markers were used for medaka. We think this is in Table 4, but we are not completely sure. Authors should show clearly which markers were used for medaka only.

> Thank you! We agree that this wasn't clear. We have now replaced the single column "Number of Fish" with two columns labeled "Number of zebrafish" and "Number of medaka", respectively.

(8) The identification of pallial subdivisions in the telencephalon, other than Dp, seems speculative. The location of Dp has been clearly shown in zebrafish larva (Miyasaka et al., 2014) and agrees with what is shown in the manuscript. However, without appropriate markers (gene expression, connections, etc.) other subdivisions, such as Dm, Dc and Dl, cannot be reliably annotated. Please provide additional support for the pallial subdivisions provided and/or soften the claims.

> Thank you! We now provide additional support for the pallial subdivisions with DAPI staining and other stainings in Figure 6a.

6. Quantitative Comparisons

(9) While the manuscript suggests that cross-species comparisons can be done within FishExplorer, we understand that tools for measurements are not yet available. Please clarify what types of analysis can be done through the platform and what must be done offline.

> Thank you.
We have added a new feature to the platform that allows the user to quantitatively compare region size and signal intensity across species via "Manhattan plots”. We have also added explanations that make it clear that users can perform more sophisticated analyses with bespoke local approaches (Python or Matlab) (page 45, line 987-993). The necessary processed datafiles, i.e the extracted arrays underlying the “Manhattan plots” are now readily available for download. (Please have a look on the “Analysis” window in FishExplorer).

(10) How was quantitative data on signal intensity and size of brain region extracted from the Fishexplorer platform? This needs to be included in the methods.

> Yes indeed, thank you.
This is taken care of by the newly added “Analysis” functionality. All relevant data of interest is extracted through simple ‘multiplication’ or ‘integration’ with the ‘mask’ volumes. This is now described in the Methods section (page 46, line 994-1000).

(11) We understand that the platform does not generate the Manhattan Plots. Based on the text, it seems this sort of analysis will be easy once data has been registered to the reference brains or generated by the platform. Please clarify this point.

> Yes, it did not.
But as mentioned above, the Manhattan plot functionality is now integrated into the platform. (Please have a look on the “Analysis” window in FishExplorer).

7. Experimental Limitations
(12) The issues faced by the authors themselves in staining, imaging, registering and analysing data are a real concern for potential users of this platform. Authors point at issues with the data, such as not very intense staining for ventral cells in the brain, and complications in the normalisation and image analysis procedures (Lines 388-404). Authors highlight differences in 5HT expression (Lines 325-328) but acknowledge they may stem from staining variability between the two species. Authors point again to the issue that differences between specimens may reflect experimental variability rather than true biological differences, is highlighted in lines 427-435. How can users overcome these issues? How can users control for or correct discrepancies? Consider providing guidance or tools to assist with normalization.

> Thank you. That’s very useful.
We have now moved a lot of examples of such issues from the results section to a new paragraph in the discussion, where we outline explicitly all potential pitfalls and traps (page 34, line 715-720). We agree that this is critical and important. We acknowledge that there is no easy, straightforward solution to these shortcomings besides careful controls and critical evaluations. The fundamental problem here is not related to atlas platforms, but rather to labeling and staining approaches and their quantification. This issue is beyond the scope of this manuscript.

(13) The methods section should describe how signal intensity and region size were quantified, particularly as this is central to the results (e.g., Manhattan plots). Also explain how the fluorescent expression was normalized.

> This is related to the point (10) above.
We believe the newly added “Analysis” functionality now takes care of this. There, all relevant data of interest are extracted by a simple ‘multiplication’ or ‘integration’ with the ‘mask’ volumes. This is now described in the Methods section. In the same section, we now also describe how fluorescent expression was normalized (page 45, line 987-993). In brief, we normalize based on average brain fluorescence, and we correct for intensity drops due to imaging depth. We have added details to the Methods section.

Minor Points
(14) Line 42: should say brain, not cerebral. And maybe better to say nuclei, rather than subregions.

> Agreed. Done. (page 3, line 46)

(15) Lines 52-63: We agree that this is a good proposal to analyse in detail brain evolution and diversity. However, as it is written now, it seems that the platform provides tools for performing all these steps, which is not the case. Hence, authors should rephrase this paragraph for clarity.

> We completely agree. We have now dramatically shortened that paragraph and limited our statements to arguments relevant to our manuscript (page 3, line 53-55; page 5, line 96-107).

(16) Line 66–67: The phrase “allow for a quantitative and direct cross-species comparison” is misleading if the platform itself does not provide the tools needed for such analyses.

> This is true. However, we believe that the added analysis functionality resolves this issue.

(17) Lines 70-78: Authors could add another reason why teleosts are such a great group for studies of brain evolution, and that is the large number of species (more than 30,000) adapted to very different ecological niches. This provides the opportunity to study closely related species that are adapted to different environments or that display slightly different behaviours or brain modifications (see Ito et al., 2007).

> Thank you! We have taken the liberty of adopting the reviewer’s language verbatim into the introduction (page 3, line 64-67).

(18) Lines 139-163 description of how IDs are decided is currently in Results, figure legend and methods. Could be briefly included in Results and, a more detailed version, in methods. The visual on this in the figures is ok, so potentially think about how to make this standalone.

> Thank you! We moved most of that stuff to the Methods section. It reads much better now (page 6, line 130-143).

(19) o Line 194: The X-ray dataset does not appear to load. Please clarify its purpose and how it is used.
> Oops. This has been fixed. The X-ray dataset can now be loaded. We used the X-ray dataset to identify neuromasts and it also proved useful for the visualisation of all non-neuronal tissues, such as heart and gills.

(20) Line 492: in Figure 5d, why do different scales for zebrafish and medaka are used in y-axis (while the same scale is used in f)?

> We used the different y-scales to facilitate inspection of the zebrafish panels in Figure 5d, and while in order to facilitate cross-species comparison, the same scale of y-axis is used in Figure 5f.

(21) Line 523–524: The shaker assay needs further justification. It appears to be a general stress test rather than a specific sensory behaviour assay. Consider referencing or describing its validation.

> We agree that shaking likely induces stress in addition to vestibular and sensory stimulation. We have now added to the text that shaker assay can cause "inducing moderate levels of stress" and provided some motivation (page 25, line 523-524).

(22) Line 545–550: The use of MS-222 requires clearer explanation regarding dosage. Also, could it have potential effects on stress circuits? Could confound behavioral or anatomical results?

> We added information on the dosage of MS-222 in the Results section (page 46, line 1005-1006).
We also included a brief discussion of the potential stress-inducing effects by MS-222 and how these effects might impact behavioral and neurophysiological studies.

(23) Lines 693-698: seems very speculative.

> Completely agree. We took that out.

(24) Lines 723-725: authors note that Dd has been identified in adult medaka but not in larval zebrafish. This is true, but there is plenty of zebrafish literature describing Dd in the adult zebrafish (e.g. Yañez et al., 2022). Consider incorporating reference to Dd in the adult zebrafish.

> Thank you. We now reference Yañez et al., 2022, Bartoszek et al. 2021 and Mueller et al. 2011, and we have added a discussion on the adult zebrafish Dd region (page 35, line 738-740).

(25) Line 986: Feeding assay is listed in Methods but not discussed in the main text or figures.

> Oops again. Thanks for pointing this out. We apologize! We had the feeding assay in the original project but then removed it because the c-fos activity was inconsistent and didn't pass our quality control.

(26) Tables and Figures: Some table legends are missing (or I could not find them). Table 1's final column is misaligned. Clarify what "HCR" stands for in Table 4.

> We apologize for this mistake! We have added all table legends in the revised manuscript.
In Table 4, "HCR" (hybrid chain reaction) is replaced with "in situ hybridization". HCR is explained in the Methods section in more detail (page 38, line 798-808).

(27) Table 1: Authors talk about "marker-gene expression pattern", but this does not seem the right term, as they used gene markers but also neurotransmitters, synaptic vesicles, etc. So "expression patterns" seems better term. I could not find a table legend.

> Thank you! We agree.
We added the table legend and removed the "marker gene" prefix in the text. "Expression patterns" is much better.

(28) Supplementary Figure 2iv: Try to include this figure in the main text. It is crucial to show that the MECE masks have been carefully done based on detailed neuroanatomical analysis.

> Thank you very much for pointing this out! We agree and have followed the reviewer's suggestion. (Please have a look at Figure 2).

Reviewer #2 (Remarks to the Author):

I co-reviewed this manuscript with one of the reviewers who provided the listed reports. This is part of the Nature Communications initiative to facilitate training in peer review and to provide appropriate recognition for Early Career Researchers who co-review manuscripts.

Reviewer #3 (Remarks to the Author):

I co-reviewed this manuscript with one of the reviewers who provided the listed reports. This is part of the Nature Communications initiative to facilitate training in peer review and to provide appropriate recognition for Early Career Researchers who co-review manuscripts.

Reviewer #4 (Remarks to the Author):

Vohra et al. present a multimodal brain atlas platform for larval medaka that is integrated with the previously established and widely used zebrafish Z Brain atlas. To construct the medaka atlas, the authors developed a manual segmentation tool and a machine-learning–assisted segmentation pipeline. They created a hierarchical nomenclature for the segmentation masks and then used them to compare anatomical features of the brains of both teleosts. Furthermore, they compared brain activity elicited by different stimuli using an immediate early gene approach.

The resources and tools described here will be highly valuable to the community, especially the integrated platform that could facilitate comparative cross-species studies and serve as a pool for anatomy and molecular and functional data. I recommend this paper for publication, provided that the following issues can be addressed in a revised version:

Major issues:

(29) Definition and homology of brain regions
Cross-species comparisons of anatomy, molecular markers, or functional activity require a rigorous, transparent definition of each region, and a clear rationale for how homology was established. For example, Figure 6 reports greater activation of the pallial Dm and Dd in medaka than in zebrafish following social stimuli. Given the evolutionary divergence and resulting telencephalic rearrangements in these species, it is unclear how Dm and Dd were delineated, and why they are considered homologous. If these assignments rely on published molecular markers or gene-expression patterns, this should be explicitly referenced.

> Thank you. We agree that we did not make our justification for the anatomical delineation of Dm and Dd sufficiently clear. We now show additional data (transverse optical sections of the telencephalon with labelings) that justify our claims for homology (new panels in Figure 7). Also, we identified the Dm and Dd areas based on a study described in a previous study that quantified the expression patterns of transcription factors. We have now made this clearer in the discussion (page 35, line 736-738).

(30) Likewise, the suggestion in line 438 that serotonergic signaling might be more prevalent in zebrafish seems premature without stronger evidence.

> We agree. We now discuss explicitly that these differences might be due to staining issues (page 34, line 715-720).

(31) The authors do provide a table summarizing how individual regions were annotated. However, this table is split across multiple pages, making it unfortunately difficult to parse.

> We have reformatted the Excel table to resolve this issue.

Moreover, the descriptions it contains are relatively brief and don't fully clarify the criteria that were used to assign label identities. This is of particular importance for key regions that the authors claim show differences between the species, such as Dm and Dd, and are therefore central to the paper's conclusions. For such comparisons to have explanatory value, the authors should include the data that were used to identify these regions in each species (anatomical, molecular, or functional). Otherwise, the observed differences may reflect differences in anatomical labeling itself rather than a true biological divergence.

> We agree. We have now added much more information to Table 3 and state explicitly all references and staining methods.

Given that cross-species comparisons are a central aim of this paper, the challenge of defining and mapping homologous brain regions across species should be discussed explicitly. If regional labels were assigned primarily by expert judgement, that limitation should be acknowledged and its impact on the conclusions carefully considered. The authors mention that many brain regions are defined using expert judgment and refer to a supplementary table summarizing this, but this is presented as a practical matter, not discussed as a limitation or scientific challenge.

> We agree. We have now added text to the discussion (page 34, line 658-660), where we detail the challenges of comparing MECE and MISC regions across species. We also explicitly discuss the confounding factors that arise from relying on expert judgement. There we also point out that this is a universal and general problem of defining and mapping homologous brain regions. We also explicitly discuss the different approaches of columnar and neuromeric approaches (page 31, line 643-644).

(32) Details of the machine-learning segmentation pipeline

To further address the issue of the anatomical comparisons, the authors developed a machine learning-assisted brain segmentation pipeline. However, the entire description of this process appears to be incomplete. How was the training data created and augmented? Was this done only on the high-level anatomical areas or further extended to the individual brain areas?

> Training data was created by manually segmenting 20 MECE masks as shown in the supplementary Figure 2-iii. We found that these high level anatomical areas were sufficient for training purposes. We now describe this more extensively in the Methods section (page 41, line 894-925).

Lines 899 and 814: what is meant by segmenting 20% of brain areas? Is it 20% of slices or is it 20% of regions? If the former, then the authors should discuss how the output compares to the conventional morphological inter-slice interpolation.

> It is the former. We find that the classifier provides slightly smoother transitions than a conventional interpolation. However, we don't think the difference is critical. We now state and discuss this in the Methods section (page 42, line 906-907).

(33) Statistical treatment of cross-species comparisons
All cross-species comparisons in this paper, except for negative control comparisons in Figs. 5b and 6c, are presented as comparisons of two mean values between the reference brains of medaka and zebrafish, without reporting the sample variability or conducting any kind of significance testing. This leaves open the possibility that some of the reported differences reflect random variation. Please report the N numbers in the figure legends, including measures of variability across fish, and perform statistical tests (e.g., ANOVA with post-hoc comparisons) to support the claims. One area where this is important is the comparisons of the functional activity with c-Fos. It is known that c-Fos expression following a stimulus can be quite variable, and making comparisons based on the mean normalized c-Fos mRNA expression for each condition could mask these differences. The authors should include the individual staining used for these comparisons.

> We agree. We have now included, wherever helpful, variance, p-values in addition to the mean.
We now provide complete datasets for all examples and all fish (that is the data underlying all Manhattan plots) for visualization and downloading. We hope this will make it easier for readers to conduct their own analyses.

Figures:
(34) General:Several figures include screen captures with very small, often unreadable text. Even the high-resolution figures at the end are not legible when printed. Please consider increasing font size or labeling clarity. The same goes for brain region labels (e.g. Figure 2d).

> As suggested, we have modified the figures so that the smallest font size is 6 and we have also tried to make the labelings clearer.

(35) Figure 1: please adjust the positioning of the panels to clearly separate them.

> We have rearranged the panels.

(36)Figures 1i and S1-vi a: Label (iv) appears to be missing.

> Thank you very much for pointing this out. We added (iv) in both figures.

(37) Figure 1e: blue-orange color-code has overlapping meanings: it corresponds to both zebrafish-medaka and hindbrain-midbrain dichotomy, which may be confusing. Please consider using a different pair of colors for one of the dichotomies.

> We agree that the color combination was confusing. To resolve this issue, we have changed the pair of colors in the hindbrain-midbrain to cyan and yellow respectively.

(38) Figure S1-iii: Please define how “thickness” was computed (number of slices?), specify units in panel d, and clarify what distinguishes panels c and e.

The thickness is indeed computed as the number of slices, which we now specify in the table. We have combined a, c, d and e into a unified table and panel.

(39) Figure 2d: Please explain R1–R7 in the legend (rhombomeres).

> We have included it in the legend, as suggested.

(40) Figure 5 and line 523: Why did the authors choose 60 minutes as the shaker stimulus duration for c-Fos expression cut-off if multiple studies indicate peak expression around 30 min? Habituation effects could arise that when combined with the c-Fos degradation would decrease the detected signal.

> This was an error on our part. We assumed that longer stimulation would provide a better signal. We agree with the reviewer that 30 minute exposure might have been a better protocol, however, since we detected significant signals with our approach, we believe that potential habituation effects are small. We now point this out explicitly in the Methods section (page 46, line 1008-1009).

(41) Figure 5 and line 541: MS-222 experiments: how long were fish in MS-222? Was the concentration calibrated to render fish non-reactive to stimuli? In case, like the authors described, of potentially insufficient anesthetic effect on medaka, how could one compare the activity between the two fish if one is not anesthetized completely and other is?

> This is a valid concern. However, it is non-trivial to rigorously assess depth of anesthesia in a larval zebrafish, beyond careful monitoring its swimming activity.
For a clean comparison across species we matched the protocols.
All fish were immersed in 160 micrograms per milliliter MS-222 (standard dose) for 60 minutes. We found that generally as little as 30 seconds of exposure is sufficient to completely anesthetize a 7dpf larval zebrafish (reference: Leyden et al. 2022; Barabási et al. 2024). For Medaka we used the same protocol because they match in size and the general physiology is similar. We further confirmed that medaka didn’t show any movements in this condition. A more explicit test for anesthesia in medaka would require concurrent brain wide imaging, an approach that is beyond the scope of this study. As such, the depth of anesthesia in medaka is not confirmed. We now state this limitation in the text (page 35, line 731-732).

(42) Figures 5 and 6: add scale bars and, more importantly, a color bar for the delta c-Fos plots. The negative controls are also presented in grayscale, while the difference is in a colormap.

> We agree. We now show the c-Fos with z-score color-coded values, which is reflected in the added color bars.

Minor issues:

(43) Line 74: “easy extrapolation”: Please consider removing the word “easy” or rephrasing more cautiously

> We agree. We removed the word “easy” (page 3, line 63).

(44) Lines 129-131 / Fig. 2: It seems like there are only two levels of hierarchy created in the brain regions: macro- and mini-masks. Later (Line 259) it seems that the mini-masks have their own hierarchy. Furthermore, in the atlas viewer itself there are more than two levels. Please clarify to avoid confusion.

> The reviewer is correct. This was confusing. There are indeed only two levels of hierarchy. We now changed "... are hierarchically nested.” to “... are nested”, which we hope resolves the confusion (page 12, line 249). Additionally, we modified the display in the atlas viewer, enlarging the icons for the five macro-masks (and the retina) to twice their size. The topologically stacked mini-masks (all MECE) all have the same icon size, representing the lack of nested hierarchy within that group.

(45) Lines 151 and 965: typo “vasculatore”

> Thanks for pointing this out. It is fixed.

(46) Lines 235-237: The color-coding of the diagram is only partially explained in the legend. Also, why is “prepare external user data” not shown in blue?

> Thank you. We changed the color of all the activities that users will perform in their workplace to light blue, and we updated the legend to explain the use of all colors (Figure 2e, Supplementary Figure 2-vi).

(47) Line 322: what is meant by normalizing in this context? Please add an explanation.

> Here, we normalized the average of fluorescent intensity in each region mask by the median intensity across all MECE masks. This explanation has been added to the Methods section (page 45, line 987-993).

(48) Line 327: whole tissue stainings in general have a high autofluorescence problem. If a claim can’t be made because of these artefacts, consider moving speculation to discussion or adding staining data to allow the reader to assess the differences themselves.

> We agree that high autofluorescence can often be an issue. We have now added an additional paragraph in the Discussion section where we address these confounds (page 34, line 715-717).

(49) Lines 341-466: This section is lengthy and includes highly interpretive content that would fit better in the Discussion. Please consider streamlining the Results and moving speculative elements to the Discussion.

> Thank you. We moved all components with speculative elements to the Discussion (page 35, line 717-720).

(50) Line 492: consider using the word “independent” instead of “orthogonal” to avoid confusion.

> Agreed! Done.

(51)— Line 698: consider providing references to support the claim about zebrafish evolving in murky water.

> Thanks for your comment. We have now added references for our claim about the divergent habitats (page 4, line 75-78):
(Engeszer et al. 2007; Spence et al. 2006) Zebrafish naturally inhabit slow-moving and murky waters.
(Hilgers and Schwarzer 2019) Medaka inhabit clearer waters with relatively fast-moving flow.

Reviewer #5 (Remarks to the Author):

Vohra et al describe a cross species platform for the analysis of brains in teleosts. The manuscript is excellent and the website and other resources promise to considerably further the field of comparative neuroanatomy. Overall, this is an excellent piece of work. I mostly have suggestions for clarification and improvements. The biggest suggested improvement I have is the incorporation of measures of variation and statistical tests to help the reader better understand the size of the effects, their reliability, and their variation.

I would also like to applaud the discussion of the challenges associated with the imaging and staining techniques used in the paper (lines 388-404; 426-435). These sorts of candid discussions are usually missing in papers. It is refreshing to see some of the technical challenges associated with this type of work made explicit. This is particularly helpful for trainees so that they have a better understanding of challenges and limitations.

> Thank you for these kind words! This is very much appreciated.

Major points

(52) While the presentation of the data using step plots through the paper is clear and intuitive, they lack a measurement of variation across animals (i.e., error bars or ribbons). With respect to the cfos data, it is stated (line 480) that variance between individuals is 'low'. But what does that mean? Indeed, if I'm understanding the data correctly, it appears that there is a significant amount of variation between individuals in some experiments (e.g., Figure 5d-1-3). It is not clear how much variation there is in other experiments in the paper (e.g., figures 2-4, 6).

> We wholeheartedly agree.
We have added individual fish plots to the Manhattan plots in the manuscript.
More importantly, we enhanced the platform's functionality to enable users to visualize each plot with either the mean, the mean plus the confidence interval, or with all the individual traces plotted on top of each other.
Users can now also download the data underlying each Manhattan plot for visualization and custom analyses.

(53) It is also not clear how 'significance' is determined aside from figure 5 that uses z-scores. For example, on line 272 it's stated that the hindbrain is larger in zebrafish than medaka. However, as best I can tell no statistics were used to make this comparison, and there are no measurements of effect sizes, and no corrections for multiple comparisons. Thus, it is difficult for the reader to assess how large, reliable or variable the findings are.

> We agree.
We have now included, throughout the manuscript and wherever helpful, variance and p-values in addition to the mean and z-scores (Figure 3, 4, 5, 6).

Minor points

(54) Line 82. A sentence or two more clearly defining the different natural environments of medaka and zebrafish (beyond just higher tolerance to salinity) would be helpful for the reader to better appreciate the common and distinct aspects of these species.

> Thank you! This is helpful. We agree and we have added an explanation of divergent habitats to the text, and added references supporting our claim about the divergent habitats (page 4, line 75-78).
(Engeszer et al. 2007; Spence et al. 2006) Zebrafish naturally inhabit slow-moving and murky waters.
(Hilgers and Schwarzer 2019) Medaka inhabit clearer waters with relatively fast-moving flow.

(55) Line 86. The reference for Ishikawa et al has 'n.d.' for the date. This should be updated.

> Thank you for your thorough reading. It was a typo and is fixed now.

(56) The number of animals used for each experiment should be included in each of the figure captions.

> We agree. We added the number of animals used in the experiments.

(57) Line 269 and figure captions. The plots would be better described as 'step plots' not 'Manhattan plots'.

> That's an interesting point. We have discussed the respective merits of these two terms for plots in some detail, and we haven't come to a cohesive conclusion.
Both have their merits. We believe that the main distinction is that "Manhattan plots" are used to highlight 'outstanding' data points that stick out above the skyline; whereas "Step plots" are used to describe a continuously varying function, which steps or transitions between discrete, but usually related, values.
We slightly prefer to stick with the Manhattan terminology for two reasons: First, it is mostly the 'skyscrapers' that we are interested in highlighting, rather than the more subtle variations in size or intensity, and second, there is no innate assumption that neighboring values ought to be related, as is generally assumed for step plots, where the 'step size' often carries some meaning. We think it is useful to avoid this implicit assumption, since the neighboring MECE regions in our plots share no natural relationships.

(58) Line 556. There is a stray '('.

> Thank you. We fixed the typo.

(59) Line 692. This point needs a citation.

> Agreed. We added a reference of cavefish's anatomical study that shows the optic tectum in cavefish, which is significantly smaller than surface fish (page 34, line 713).

(60) While I appreciate the challenge of 'age matching' zebrafish and medaka, the discussion should include the point that some of the differences observed may be due to different rates of development of different parts of the brain. There is no guarantee that all parts of the brain or body develop at the same rate in zebrafish and medaka (or any two species for that matter) and thus that there may be no such timepoint where everything is 'matched'. This is an important caveat for this type of developmental comparative work in general.
Related to the above point, I would be interested to see a small expansion of the discussion as to how adult and juvenile brains may be incorporated into this resource. Being mature, adult brains may provide a more stable basis for comparative work. How would your resource handle the drastically different sizes, number of brain regions, and need to use different histological techniques like tissue clearing that can affect brain size or morphology.

> Thank you, this is very helpful. We have added a discussion of the confounds and limitations of differentially developing brain regions within and across species.
We have also expanded the Discussion section, where we describe the planned expansion of the platform to incorporate additional species and developmental stages (page 33, line 696-700).
The differences in size and number of regions will be addressed by using several developmental 'standard' brains to anchor the developmental stages (e.g. 5dpf, 7dpf, 9dpf…….21 dpf) and then interpolating between them.

(62) The website is well designed and easy to use. My only suggestion would be to consider adding a functionality that allows the user to directly compare images from different species. An option for overlays would be great, but I don't know how feasible that would be since different species wouldn't necessarily be in the same coordinate space. However, an option for side-by-side viewing would make comparative analysis easier instead of needing to switch between images. As best I could tell, this functionality was not there.

> We agree! As the reviewer already pointed out, direct overlay across species is not informative since they live in different coordinate systems. For such analyses the Manhattan plot approach is more suitable. We have now implemented cross-species comparison 'Analysis' functionality in our platform. Currently, the cross-species analysis is limited to Manhattan Plots.
Direct comparison of images would be easier with side-by-side viewing in the same window. We have a deeper understanding of this issue. Indeed, showing side-by-side viewers is not an issue (as we mention below). However, technical difficulties arise when selecting brain regions, markers and lines, because the ontological trees of both species are different. Some regions or lines are missing in one species or the other. Technical advancements in mapping and visualization of such trees are needed to clearly show these differences in the same tree for both species. One can anticipate such challenges for other kinds of data, e.g., visualization of single cells. We are working on these challenges and anticipate that future generations of our platform will be able to accommodate side-by-side comparisons within the same window. For now, users have two options for direct comparison of images. First, open another Fishexplorer

window and select the comparand there. Second, download both Image files and compare them locally in Fiji or ImageJ.

Reviewer #5 (Remarks on code availability):

(63) The website appears useable. I did not try uploading new data to it however.
Thank you!

REVIEWER COMMENTS - Vohra et al.

We appreciate all of the reviewers' comments. In the following, we carefully address each comment in detail. In this document, our answers to the comments in this second revision are written in blue letters, our answers to the comments in the first revision are written in green letters, and the corresponding changes are highlighted in yellow in the revised manuscript "**manuscript_highlighted_revised_version2**.pdf". The page and line numbers in this document correspond to those in the revised manuscript.

Reviewer #1 (Remarks to the Author):

Further Reviewer 1 Comments to Author following rebuttal:

(3) There is currently no way for users to create new reference brains within the platform. This limits extending the platform to other species and stages. Please clarify whether such tools will be developed and how. If not, please discuss this as a shortfall of the platform.

> Indeed, the current platform is not compatible with - nor intended for - the ingestion and analysis of species other than zebrafish and medaka.
In its current form, it lacks the functionality for cross-developmental analysis

There are still references in the paper that do make it sound more like that is what they envisage it being used for, some examples:
Line 100-101 The platform's database can be expanded in many ways, e.g. in terms of tissue types, biological species and developmental stages.
Line 112 multi-species atlas
Line132 if we include more species
So we think the authors need a clearer statement on this point.

>>> Thanks. Yes, this is correct. The current state of the platform does not yet allow users to integrate different ages and other species. In the text (**page 3, lines 101-103**), we have made this clear by stating "In its present form it allows for the inclusion of new tissue types, and future versions should also allow the addition of different species and later developmental stages."

(8) The identification of pallial subdivisions in the telencephalon, other than Dp, seems speculative. The location of Dp has been clearly shown in zebrafish larva (Miyasaka et al., 2014) and agrees with what is shown in the manuscript. However, without appropriate markers (gene expression, connections, etc.) other subdivisions, such as Dm, Dc and Dl, cannot be reliably annotated. Please provide additional support for the pallial subdivisions provided and/or soften the claims.
> Thank you! We now provide additional support for the pallial subdivisions with DAPI staining and other stainings in Figure 6a.

We think that the authors' response does not address the core of the concern, although we appreciate their effort in adding new analyses. The use of DAPI (and HuC) and the other general stainings does not provide sufficient evidence for the identity of pallial subdivisions. The additional markers used by the authors (GFAP, vglut2a, synapsin and HuC) provide valuable information on the general organization of the telencephalon; however, they are not specific to pallial domains and therefore do not constitute evidence for their identity. None of these markers can discriminate between Dm, Dc and Dl. As stated in the original comment, without

appropriate markers (e.g. domain-specific gene expression and/or tract-tracing data), the annotation of Dm, Dc and Dl remains speculative. The authors should therefore either provide markers supporting the identity of these pallial domains or explicitly soften the terminology throughout the manuscript (e.g. "putative Dm/Dc/Dl based on topography") and clearly state that these assignments are tentative in the Results and Discussion. In its current form, Figure 7 and the associated text still imply a level of anatomical certainty that is not supported by the data.

>>> Thank you. Now we performed an anti-parvalbumin immunohistochemistry experiment, inspired by a previous report on labeling the pallium subdivisions in adult zebrafish (Mueller T et al. 2011). In this study, parvalbumin-positive cells are observed in Dl and Dc in the adult brain. In our new experiment with larvae, as shown in the new version of **Figure 7** and **Supplementary Figure 7**, we detected very strong signals in the olfactory bulb and olfactory epithelium. We also observed an parvalbumin expression in the pallium as well as signals in cell bodies of Dl and axon fibers of the Dc. This result aligns with the staining result in adult zebrafish (Mueller T. et al. 2011). We confirm that the signal boundaries are consistent with the current MISC masks of larval zebrafish. (**Figure 7: pages 19-20, lines 611-618**)

>>> We added the following sentence in the manuscript (**page 17, lines 582-588**):
"By quantifying the marker gene expression, we found that medaka display stronger synapsin-positive signals in the dorsal area of the pallium (Dd, ID: 3005), which is consistent with the expression pattern in the adult pallium (Mueller T et al. 2011) (**Supplementary Fig. 7**). In contrast, in zebrafish, we found that parvalbumin expresses in the cell bodies of the lateral area of the pallium (Dl) and in axon fibers in the central part of the pallium (Dc), which is consistent with the results of the anti-parvalbumin staining in adult zebrafish. Additionally, strong synapsin staining was detected in (Dc, ID: 3003)."

>>> We also added the imaging stacks in FishExplorer.

Another issue concerns the discussion of the dorsal pallium (Dd). The adult medaka study cited (Isoe et al.) convincingly demonstrates the existence of an epigenetically and clonally distinct dorsal pallial compartment in medaka. However, the statement that such a region "has not been confirmed to exist in zebrafish larvae" may be misleading. The absence of evidence in zebrafish does not necessarily imply the absence of a homologous structure. Rather, it may reflect methodological limitations, as a detailed study of the larval zebrafish pallium comparable to that in medaka is still missing, as well as unresolved issues of nomenclature and homology in the teleost telencephalon.
A dorsal pallial domain (Dd) has been described in adult zebrafish-based connectivity (Yáñez et al., 2022), and in multiple other teleost species (Braford et al., 2009; Burmeister et al., 2009; Demski et al., 2013). Therefore, from a comparative and evolutionary perspective, it seems plausible a Dd-like domain may also be present in zebrafish, including at larval stages, but has not yet been adequately characterized. Accordingly, the current lack of evidence in zebrafish larvae should be interpreted with caution, and the wording in the manuscript should reflect this uncertainty.

>>Note: This data is in Fig 7 not Fig 6a. Delineated areas are very difficult to see in the transverse sections, so I perhaps make these images bigger.

>>> Thank you for your insightful and careful comments! We have increased the size of the images. We concur that greater care should be exercised when describing the dorsal pallium in larval zebrafish. We have expanded the Discussion Section to include descriptions of the potential Dd region in larval zebrafish as follows: "Also the connectivity study in adult zebrafish across the compartments in the pallium and other brain

regions support the idea of existence of a unique anatomical compartment in the dorsal pallium (Yáñez et al., 2022). In addition, previous works in multiple other teleost species strongly suggest the dorsal pallium is conserved across teleost (Braford et al., 2009; Burmeister et al., 2009; Demski et al., 2013). Therefore, from a comparative and evolutionary perspective, it seems plausible that a Dd-like domain is present in zebrafish, including at larval stages, however it needs more careful characterization." (**page 23, lines 757-750**).

Minor points associated with this comment
(a) Reference needs updating. At the moment, anatomical delineation of most pallial areas seem to be based on a preprint:

1260-1261 33. Isoe, Y. et al. Epigenetically distinct synaptic architecture in clonal compartments in the teleostean dorsal pallium. Preprint at https://doi.org/10.1101/2022.10.01.510385.

Reference should be updated, as doi doesn't work and it is now published https://elifesciences.org/articles/85093

>>> Thank you very much! We have updated the reference (**page 43, line 1305**).

(b) Line 619-620 "defined by the anatomical boundaries labeled by transgenic lines"

No transgenic lines or any other markers are listed in the Table for telencephalon for the pallial areas apart from synapsin staining and references Isoe et al, 2023 (see separate note about updating this reference).

>>> Thanks! We have updated **Table 3** by adding transgenic lines that were utilized to delineate anatomical boundaries.

(C) Line 592-593 Labeling synapse density using a presynaptic marker, synapsin, which is ubiquitously expressed in all vertebrates, suggests different compartment patterns of computation in the telencephalon between the two species

We don't see synapsin as an available label on the Fishexplorer atlas and need more explanation on how that marker would differentiate between subdomains.

>>> You are right. Thank you! This was a mistake on our side. The FishExplorer atlas now includes anti-synapsin imaging stacks.

Minor Comments on additional text

Additional text Lines 753-756

Lines755-756 but we also share the recipes and protocols for registration to reference brains, and mask generation for other species and developmental stages (see 'Cross-species analysis' tutorial in the FishExplorer).

We couldn't initially find this as it is labelled "cross-section analysis" on the tutorial webpage list.

>>> Thank you very much for pointing this out! Now the name has been updated to "Cross-species analysis" on our platform website.

Notes on the utility of the platform itself

Having tried the explorer on several different computers, it is still not problematic for visualizing expression patterns. The images used throughout the paper showing maximum projections of zstacks are not viewable using the viewer - in 3D view it is still a horizontal slice not really 3D. It should be made clear that these images are not from the viewer but from raw data.

>>> We agree! We made it clear in the figure legends in **Figure 2, Figure 4, Figure 6, Supplementary Figure. 2-i**, that "Maximum projections of registered imaging stacks (generated outside of the platform) are shown".

(1) Analysis tools do not seem available to the user which will discourage use and adoption by the community. Authors should comment on this more openly in the manuscript.
> We agree that this was a serious omission. We have now added a dedicated analysis section to the platform where users can generate Manhattan plots in various configurations. (Please have a look at the FishExplorer and Comparative analysis tutorial (https://fishexplorer.zib.de/sandbox/)).

The analysis tool doesn't allow us to download anything after comparing expression patterns for different brain regions. The ideas are good, but the implementation is still problematic.

>>> We appreciate the reviewer's thorough testing of the platform and apologize for any inconvenience this issue has caused. We have fixed the issue with the download functionality, so it now works on the platform.

Reviewer #2 (Remarks to the Author):

I co-reviewed this manuscript with one of the reviewers who provided the listed reports. This is part of the Nature Communications initiative to facilitate training in peer review and to provide appropriate recognition for Early Career Researchers who co-review manuscripts.

Reviewer #3 (Remarks to the Author):

I co-reviewed this manuscript with one of the reviewers who provided the listed reports. This is part of the Nature Communications initiative to facilitate training in peer review and to provide appropriate recognition for Early Career Researchers who co-review manuscripts.

Reviewer #4 (Remarks to the Author):

The authors addressed the majority of our comments in the response, and we appreciate the effort they put into the revision. That said, we find that several points are not yet fully addressed in the revised manuscript. We ask the authors for the following minor, but necessary changes:

>> (29) Definition and homology of brain regions

The text addresses our concerns; however, please include high-resolution versions of the synapsin/H2B:GFP magenta/green overlays in Fig. 7b. In the current figure, the resolution is not sufficient to clearly evaluate the overlap between synapsin antibody staining and the Dd mask.

>>> Thank you. As the original image stacks did not have a uniform resolution, but a coarser resolution in the depth (dorsal-ventral) axis, $(\Delta x, \Delta y, \Delta z) = (1.0, 1.0, 2.0)$ (μm), the images in Figure 7a don't display the high-resolution. To support the evidence for the pallium MISC masks, we performed anti-parvalbumin immunostaining, drawing inspiration from a previous report on the adult zebrafish pallium (Mueller T et al. 2011). In the revised manuscript, we included the staining result in **Figure 7** (**pages 19-20, lines 612-619**) and **Supplementary Figure 7.**

>> (30) Likewise, the suggestion in line 438 that serotonergic signaling might be more prevalent in zebrafish seems premature without stronger evidence.
> We agree. We now discuss explicitly that these differences might be due to staining issues (page 34, line 715-720).

The Discussion now explains the challenges of cross-dataset normalisation more adequately, which is helpful. However, the Results text in the lines 436-443 –

"...suggesting that serotonergic signalling in motor modulation may be more prevalent in zebrafish."; "Perhaps the modulation of visual signals is under tighter dopaminergic control in this species."; …, suggesting a possibly enhanced role of glial processing, which is known to be under the influence of noradrenergic inputs from nearby hindbrain regions."

… still needs to be revised to match this. As written, the Results section continues to make claims about differential serotonergic, dopaminergic, and noradrenergic signalling, but these are later qualified (and potentially undermined) in the Discussion due to the risk of misinterpretation. In our view, such claims should not be presented as part of the findings/Results, especially if they are subsequently framed as potentially unreliable.

Please retain the quantification in the Results, but move any speculative or interpretive statements to the Discussion, where the limitations can be appropriately addressed alongside them.

>>> We appreciate your feedback! We agree that the text was misleading. We have moved all speculative or interpretive statements to the "Discussion" section, and also pointed out the limitations (**page 22, lines 713-720**).

>> Lines 899 and 814: what is meant by segmenting 20% of brain areas? Is it 20% of slices or is it 20% of regions? If the former, then the authors should discuss how the output compares to the conventional morphological inter-slice interpolation.
> It is the former. We find that the classifier provides slightly smoother transitions than a conventional interpolation. However, we don't think the difference is critical. We now state and discuss this in the Methods section (page 42, line 906-907).

The revised lines 906-907 – "From the 9 fully labelled binary-segmented brain regions, we generated training data with 20%, 25%, 30% and 35% of slices using the deep learning panel of the Fiji plug-in, NeMO" – do not address the second question about the comparison with the morphological inter-slice interpolation.

>>> We would like to thank the reviewer for bringing the method of morphological inter-slice interpolation to our attention. We incorporated this method in our Fiji plugin and compared it with the deep learning-based method (**page 27, lines 944-952**). The *Table−1* (below) provides a methodological comparison between 2D U-Net and inter-slice interpolation concerning algorithmic approach, required input data, output data, computation cost and use case. The *table−2* (below) provides quantitative comparison of 2D U-Net and inter-slice interpolation across several brain regions using intersection over union (IOU) as an evaluation metric. The results of both the methods are quite comparable. Both the algorithms work well when there is a single object in an image and perform more errors when the topology of the object changes from one-many in an image. This *Table−2* is added as **Table 5** in the manuscript.

Table −1: Methodological comparison

	**UNET**	**Inter-slice interpolation**
**Algorithmic approach**	It uses an encoder-decoder convolution neural network to classify each pixel in an image.	It uses an iterative dilation-based approach to determine the change of shape between the input slices.
**Required input data**	In order to train the model, it requires some images and their respective masks.<br>Testing: images<br>After training, only the images are needed as input.	It requires only a stack of masks as the input, arranged in a structured manner that maintains the order of the slices
**Output data**	Given a test image, it generates a predicted mask as output.	Given a stack of images with interspersed masks, it generates masks for all slices as output.
**Computation cost**	Expensive	Low
**Use case**	It can be used to extract anatomical structures from image stacks without having to label any slices in advance.	It can be used to extract anatomical structure from image stacks, if about every fifth image is labeled. .

Table 2: Quantitative comparison

**Region**	**Deep learning IOU (in %)**	**Inter-slice interpolation IOU (in %)**
**Forebrain**	94.33	96.41
**Midbrain**	95.26	96.53
**Hindbrain**	95.56	96.88

**Stratum Periventiculare**	91.48	93.84

>> (38) Figure S1-iii: Please define how "thickness" was computed (number of slices?), specify units in panel d, and clarify what distinguishes panels c and e.

> The thickness is indeed computed as the number of slices, which we now specify in the table. We have combined a, c, d and e into a unified table and panel.

The thickness is still not defined in the (now) Figure S2-iiid panel or its caption, and the difference between panels c and e has not been clarified.
Please indicate thickness in defined units (micrometers) and modify the caption to: "List of the remaining 13 regions and their respective thicknesses in micrometers." In addition, please revise the captions as follows: Panel c: "…for each of the initially annotated 8 regions using the selected mode." Panel e: "…for each of the remaining 13 regions annotated."

>>> Thank you for your comment! We have modified the caption as suggested. The unit of physical thickness in the medaka image stacks is 1.5 µm, which is now also explicitly stated in the caption (**page 33, lines 1094-1096**).

>> (41) Figure 5 and line 541: MS-222 experiments: how long were fish in MS-222? Was the concentration calibrated to render fish non-reactive to stimuli? In case, like the authors described, of potentially insufficient anesthetic effect on medaka, how could one compare the activity between the two fish if one is not anesthetized completely and other is?
> This is a valid concern. However, it is non-trivial to rigorously assess depth of anesthesia in a larval zebrafish, beyond careful monitoring its swimming activity. For a clean comparison across species we matched the protocols. All fish were immersed in 160 micrograms per milliliter MS-222 (standard dose) for 60 minutes. We found that generally as little as 30 seconds of exposure is sufficient to completely anesthetize a 7dpf larval zebrafish (reference: Leyden et al. 2022; Barabási et al. 2024). For Medaka we used the same protocol because they match in size and the general physiology is similar. We further confirmed that medaka didn't show any movements in this condition. A more explicit test for anesthesia in medaka would require concurrent brain wide imaging, an approach that is beyond the scope of this study. As such, the depth of anesthesia in medaka is not confirmed. We now state this limitation in the text (page 35, line 731-732).

The revised lines 731-732 in the Discussion " In medaka, we found activation in different areas of the brain, which might indicate a different chemical sensation than in zebrafish." do *not* state or discuss any limitations and the Results contain several references to "general" or "full" anaesthesia.

We ask the authors to remove references to "under general anaesthesia", "full anaesthesia", interpretations ("puzzling") and speculative statements (neuromasts as chemical sensors) from the Results. Additionally, please correct the tautological phrasing in lines 730-731 (the "consistent with previous findings" statement appears twice in the same sentence) and discuss limitations of comparing anaesthesia levels across species.

>>> We removed "puzzling" and the references relating to anesthesia from the Results.
Thank you! We have implemented all of the suggested changes (**page 22, lines 736-741**). Also in the text, we added that "Since comparing anesthetic levels across species is difficult, we need to be careful when adjusting the amount of MS222 when using it as an anaesthetic." (**Page 22, lines 740-741**)

Please also check the MS-222 concentrations in the revised manuscript; you state you used the standard dose of 160 ug/mL (matching the literature) but the manuscript states 60 ug/mL multiple times (line 472, line 1005).

>>> Thank you! We have double checked, and it was 100 µg/ml, which is the same as in the following reference:
Barabási, D.L., Schuhknecht, G.F.P. & Engert, F. Functional neuronal circuits emerge in the absence of developmental activity. *Nat Commun* 15, 364 (2024). (**page 14, line 464, page 29, line 1032**)
We apologize for the mistake.

Reviewer #5 (Remarks to the Author):

The authors have done a great job responding to reviewer comments and updating their paper and website. They have addressed all of my concerns; however, it looks like there are no error bars on some of the images, e.g., Figures 4 and 5 for 5-HT, TH, and Oxt.

>>> That is correct. For the zebrafish dataset of 5-HT, TH, and Oxt, we used data from Randlett et al. (2015) and only have the average image stacks**;** that is, we cannot provide error bars.
.

REVIEWER COMMENTS - Vohra et al.

We appreciate all of the reviewers' comments. In the following, we carefully address each comment in detail. In this document, our answers to the comments in this third revision are written in blue letters, and the corresponding changes are highlighted in yellow in the revised manuscript "**manuscript_highlighted_revised_version3**.pdf". The page and line numbers in this document correspond to those in the revised manuscript.

Reviewer #1 (Remarks to the Author)

Thanks to the authors for their additional revisions. We are happy with the manuscript to be published in its current state but do have some suggestions for the authors to consider. These primarily relate to the pallial subdomain comparative section. In the light of the new results, we still have some concerns about figure 7 and the related sections:

Authors comment>>> Thank you. Now we performed an anti-parvalbumin immunohistochemistry experiment, inspired by a previous report on labeling the pallium subdivisions in adult zebrafish (Mueller T et al. 2011). In this study, parvalbumin-positive cells are observed in Dl and Dc in the adult brain. In our new experiment with larvae, as shown in the new version of Figure 7 and Supplementary Figure 7, we detected very strong signals in the olfactory bulb and olfactory epithelium. We also observed an parvalbumin expression in the pallium as well as signals in cell bodies of Dl and axon fibers of the Dc. This result aligns with the staining result in adult zebrafish (Mueller T. et al. 2011). We confirm that the signal boundaries are consistent with the current MISC masks of larval zebrafish. (Figure 7: pages 19-20, lines 611-618)

The authors appropriately selected a marker (parvalbumin-PV) based on the adult literature to visualize Dl and Dc in the larva. However, they only apply it in zebrafish. Given that the manuscript compares two species, it would be desirable to analyse PV in medaka as well.

> Thank you for this constructive suggestion. In response, we performed anti-parvalbumin (PV) immunostaining in medaka larvae and have now included these data in **Supplementary Figure 7** (Page 41). As shown in **Supplementary Figure 7c**, we did not detect PV expression in the medaka telencephalon. To verify that the immunostaining worked properly, **Supplementary Figure 7d** shows robust PV immunoreactivity in the cerebellum, including the Purkinje cells of the Valvula cerebelli (Va) and Corpus cerebelli (CCe), as well as in skeletal muscles, consistent with previous reports. Thus, although the staining was successful, we did not observe PV-positive cells in the medaka pallium.

Because PV expression was absent from the medaka telencephalon, we did not include these data in **Figure 7**, which focuses on markers that delineate pallial subdivisions. Instead, we have added the medaka PV results to the Results section (Page 20, Lines 601–603), as follows:
"We also performed anti-PV staining in medaka larvae. Although strong PV immunoreactivity was detected in the cerebellum (Endo et al., 1985) and skeletal muscles (Leberer et al., 1986; Brownridge et al., 2009), no PV-positive cells were observed in the pallium (Supplementary Figure 7c)."

We have also incorporated this finding into the Discussion (Page 25, Lines 764–768) to highlight the interspecies difference:
"PV has been used as a marker of Dl and Dc in adult zebrafish, and we detected PV expression in the corresponding pallial regions of larval zebrafish. In contrast, PV expression was not detected in the larval medaka pallium. This finding highlights substantial interspecies differences in gene expression and cellular composition within the teleost pallium."

Here is the Supplementary figure 7 and its legend.

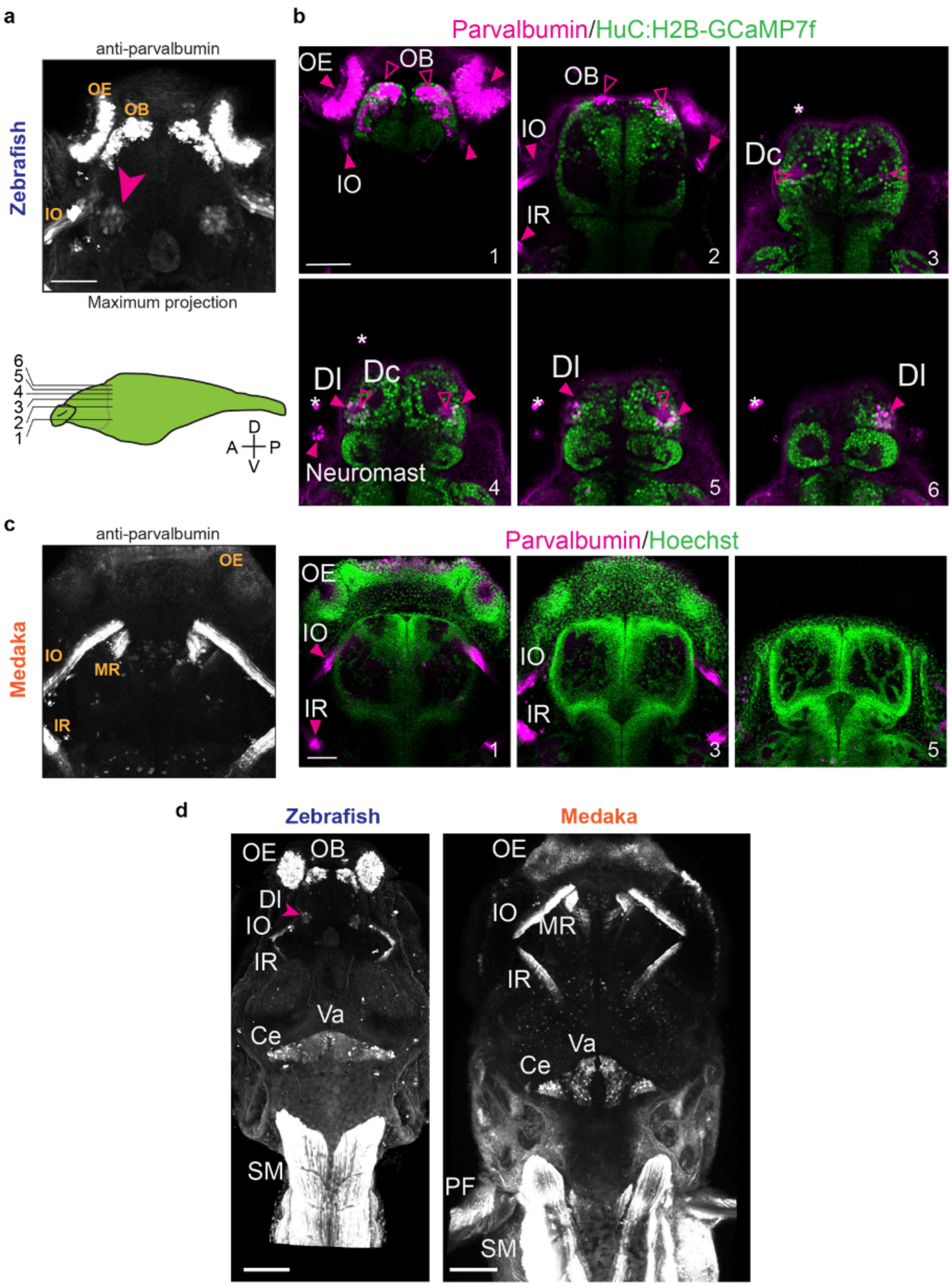

Supplementary Figure 7. Parvalbumin expression in the brain.

(**a**) **Top:** Maximum-intensity projection of anti-parvalbumin (PV) immunostaining in the larval zebrafish telencephalon. The pink arrowhead indicates PV signals in the pallium. Strong PV expression is observed in the olfactory epithelium (OE), olfactory bulb (OB), inferior oblique muscle (IO), and inferior rectus muscle (IR). **Bottom:** Schematic lateral view of the larval zebrafish brain. Horizontal lines indicate the imaging planes shown in **(b)**. Scale bar, 50 µm. A, anterior; P, posterior; D, dorsal; V, ventral.

(b) PV expression at different horizontal planes in the larval zebrafish brain. Magenta, PV; green, Tg(HuC: H2B-GCaMP7f). Imaging plane positions are indicated in the lower right corner of each image. Filled

arrowheads indicate cell bodies, and open arrowheads indicate axon fibers. Asterisks indicate nonspecific signals. Dc, central zone of the pallium; Dl, lateral zone of the pallium; IR, inferior rectus muscle. Scale bar, 50 μm.

(c) Left: Maximum-intensity projection of anti-PV immunostaining in the larval medaka telencephalon. MR, medial rectus muscle. **Right:** PV expression at different horizontal planes in the larval medaka brain. Magenta, PV; green, Hoechst. Imaging plane positions are indicated in the lower right corner of each image. Scale bar, 50 μm.

(d) Maximum-intensity projections of anti-PV immunostaining in whole brains of larval zebrafish (left) and medaka (right). The magenta arrowhead indicates PV signal in the zebrafish Dl region. The regions that express PV are labeled. Ce, corpus cerebelli; IO, inferior oblique muscle; IR, inferior rectus muscle; MR, medial rectus muscle; OE, olfactory epithelium; OB, olfactory bulb; PF, pectoral fin; SM, skeletal muscle; Va, valvula cerebelli. Scale bar, 100 μm.

Newly added references:

- Endo T, Kobayashi S, Onaya T. Parvalbumin in rat cerebrum, cerebellum and retina during postnatal development. Neurosci Lett. 1985 Oct 10;60(3):279-82. doi: 10.1016/0304-3940(85)90590-7. PMID: 3906443.

- Leberer E, Pette D. Neural regulation of parvalbumin expression in mammalian skeletal muscle. Biochem J. 1986 Apr 1;235(1):67-73. doi: 10.1042/bj2350067. PMID: 3741391; PMCID: PMC1146649.

- Brownridge P, de Mello LV, Peters M, McLean L, Claydon A, Cossins AR, Whitfield PD, Young IS. Regional variation in parvalbumin isoform expression correlates with muscle performance in common carp (Cyprinus carpio). J Exp Biol. 2009 Jan;212(Pt 2):184-93. doi: 10.1242/jeb.021857. PMID: 19112137.

Regarding PV expression, the data does not appear align very well with the zebrafish masks,. Supplementary Figure 7a shows PV expression located rather caudally, with a relatively large gap between the PV+ expression in the Dl and olfactory bulb. Based on this PV data, the authors have refined labels in synapsin expression (fig. 7c), but not the pallial masks. For instance, Dl mask extends much more than the Dl PV+ area (comparison between Supplementary figure7a and Fig. 7b). These mis-matches weaken confidence in the proposed anatomical subdivisions. A single marker is sometimes insufficient to define pallial boundaries and we suggest that the authors should explicitly soften the terminology throughout the manuscript (e.g., “putative Dm/Dc/Dl based on topography”) and clearly state in both the Results and Discussion that pallial masks are tentative.

> Thank you for this thoughtful comment. We agree that a few molecular markers are insufficient to define pallial boundaries with certainty, particularly given the complexity of the zebrafish pallium and the likely presence of multiple distinct cell populations. Furthermore, because we detected PV expression in the zebrafish pallium but not in the medaka pallium, the correspondence of pallial subdivisions between these two species remains uncertain. We therefore agree that the anatomical terminology should be more cautious.

Following the reviewer's suggestion, we have softened the terminology throughout the manuscript and now explicitly describe the pallial subdivisions as **putative Dm, Dc, and Dl regions based primarily on topography**. We also state clearly in both the Results and Discussion that these pallial masks are tentative and should be regarded as a working anatomical framework rather than definitive boundaries.

The following sentence has been added to the Results section (Page 19, Lines 588–591):

“The IDs of the new MISC masks defined in the telencephalon contain '3' in the seventh digit (**Fig. 7a**). Because only a limited number of molecular markers are currently available to define pallial subdivisions,

the boundaries of the putative Dm, Dc, and Dl regions are based primarily on topography and should be considered tentative. ”
Also in the **Figure 7** legend (Page 22, Lines 627–628):
“(**b**) MISC masks of putative pallial subregions. Their boundaries are based primarily on topography and informed by immunostaining and transgenic marker expression.”

We have also added the following statement to the Discussion (Page 25, Lines 769–772):
“Therefore, from a comparative and evolutionary perspective, a Dd-like domain may also be present in zebrafish, including at larval stages, although this will require further characterization. Therefore, the putative Dm, Dc, and Dl masks presented in this study are based primarily on topographical features and should be regarded as tentative anatomical assignments.".

>>> “we found that medaka display stronger synapsin-positive signals in the dorsal area of the pallium (Dd, ID: 3005), which is consistent with the expression pattern in the adult pallium (Mueller T et al. 2011) (Supplementary Fig. 7”
In what sense the reported expression pattern is consistent with Mueller et al. as that study did not analyze synapsin or any other synaptic marker and indeed, according to Mueller et al., a distinct Dd subdivision is absent in zebrafish.

> Thank you for pointing out this mistake. We apologize for the incorrect citation. The statement was intended to refer to **Isoe and Nakamura et al. (2023)** rather than **Mueller et al. (2011)**. While Mueller *et al.* did not examine synapsin expression or other synaptic markers, Isoe and Nakamura *et al.* analyzed the adult medaka telencephalon and reported distinct epigenetic transcriptional regulation of synapse-related genes in the dorsal pallial region. We have therefore replaced the incorrect citation with the appropriate reference throughout the manuscript.

>>> We also added the imaging stacks in FishExplorer.
We couldnt find this data in FishExplorer please check if anti-Parvalbumin and anti-synapsin date are present.
> Thank you for your comment. We have now for sure implemented anti-PV and anti-synapsin data in FishExplorer. Here is the screenshot of the imaging stack views in FishExplorer.

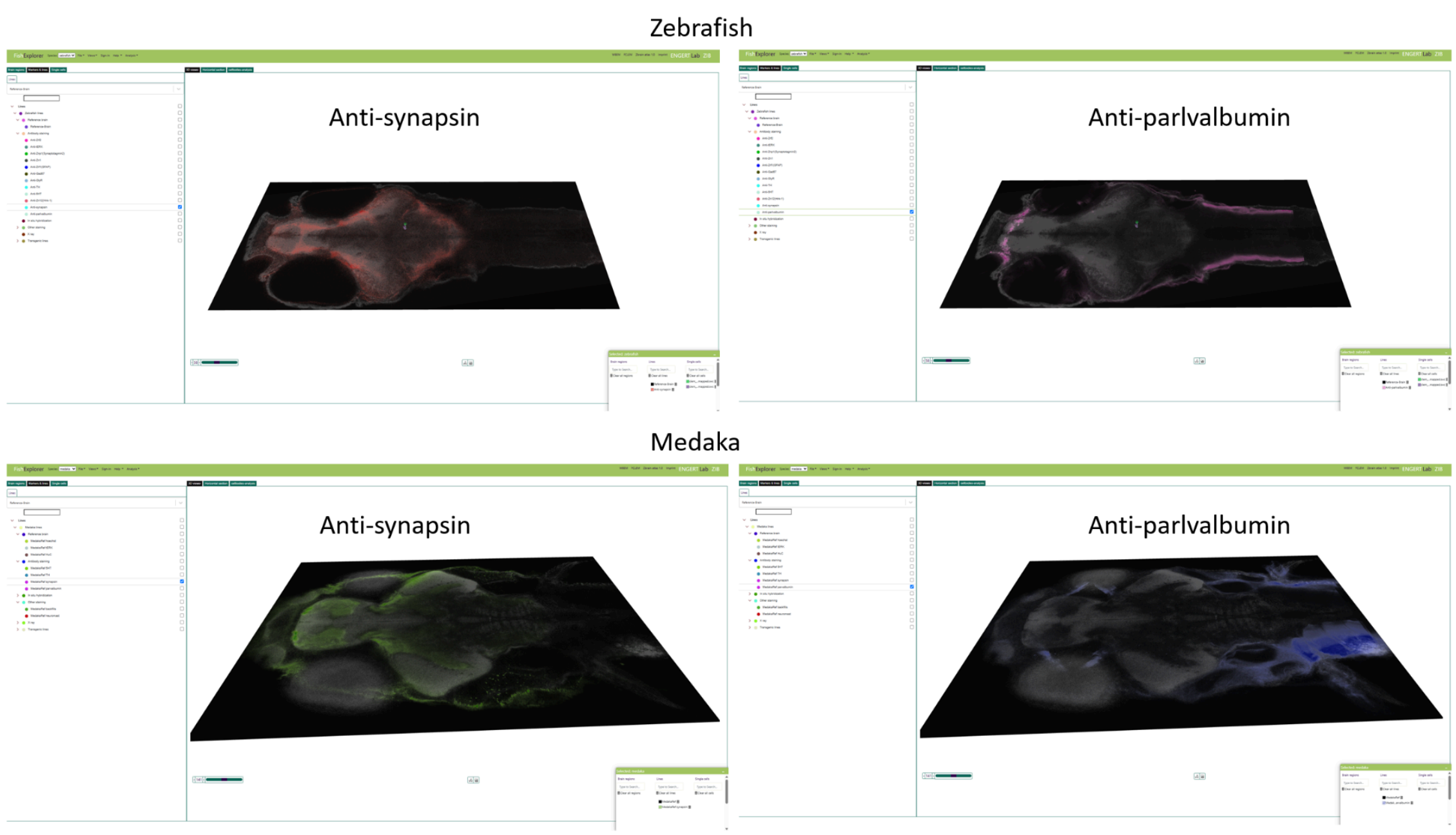

Thank you for your insightful and careful comments! We have increased the size of the images. We concur that greater care should be exercised when describing the dorsal pallium in larval zebrafish. We have expanded the Discussion Section to include descriptions of the potential Dd region in larval zebrafish as follows: “Also the connectivity study in adult zebrafish across the compartments in the pallium and other brain regions support the idea of existence of a unique anatomical compartment in the dorsal pallium (Yáñez et al., 2022). In addition, previous works in multiple other teleost species strongly suggest the dorsal pallium is conserved across teleost (Braford et al., 2009; Burmeister et al., 2009; Demski et al., 2013). Therefore, from a comparative and evolutionary perspective, it seems plausible that a Dd-like domain is present in zebrafish, including at larval stages, however it needs more careful characterization.” (page 23, lines 757-750).

The authors may want to comment on how the possibility that Dd exists in zebrafish affects the interpretation of their results for the telencephalon and the comparison between zebrafish and medaka.

> Thank you for this insightful comment. We agree that the possible existence of a Dd subdivision in zebrafish is an important consideration when interpreting our comparative analysis.

Importantly, we do not believe that this possibility changes the main conclusions of the present study. Our goal is not to establish definitive homology of pallial subdivisions between zebrafish and medaka, but rather to provide a quantitative anatomical framework that enables systematic comparison across species. Whether Dd is recognized as a separate subdivision or incorporated within another dorsal pallial domain does not affect the quantitative analysis itself; it primarily affects the interpretation and nomenclature of the corresponding masks.

To clarify this point, we have expanded the Discussion to state that “the current pallial masks represent tentative anatomical assignments based on the best available evidence and may be refined as additional molecular, developmental, and connectivity data become available. One advantage of the MISC framework is that it is designed to accommodate such refinements while preserving the quantitative analyses based on the atlas” (Page 25, Lines 772-776).

Reviewer #2 (Remarks to the Author):

I co-reviewed this manuscript with one of the reviewers who provided the listed reports. This is part of the Nature Communications initiative to facilitate training in peer review and to provide appropriate recognition for Early Career Researchers who co-review manuscripts.

Reviewer #3 (Remarks to the Author):

I co-reviewed this manuscript with one of the reviewers who provided the listed reports. This is part of the Nature Communications initiative to facilitate training in peer review and to provide appropriate recognition for Early Career Researchers who co-review manuscripts.

Reviewer #4 (Remarks to the Author):

Thank you for the revision. Our comments were addressed.

And in response to the editor's additional request, we have now clarified in the manuscript why error bars are not shown for the 5HT, TH, and Oxt analyses in Figures 4 and 5.

The glutamate and GABA analyses were performed using original imaging datasets in the lab, allowing us to quantify biological variability and include error bars. In contrast, the 5HT, TH, and Oxt datasets were obtained from the Z-Brain atlas (Randlett et al., 2015), for which only averaged image stacks are available and the original individual datasets are not publicly accessible. Consequently, measures of biological variability could not be calculated for these markers. We have now explicitly stated this in the figure legends and Methods.

In the figure legends, the following sentences were added (Page 11, Lines 343–345; Page 15, Lines 462–465;):
"For the 5HT, TH, and Oxt analyses, average image stacks from the Z-Brain atlas (Randlett et al., 2015) were used. Because the original individual datasets are not publicly available, biological variability could not be estimated and error bars are not shown for these markers."
In the Results section, we added the following (Page 10, Lines 318–319):
"For 5HT, TH, and Oxt, we analyzed the corresponding average image stacks from the Z-Brain atlas (Randlett et al., 2015)."
In the Methods section, the following sentences were added (Page 31, Lines 1053–1056):
"The 5HT, TH, and Oxt datasets were obtained from the Z-Brain atlas (Randlett et al., 2015). These datasets consist of averaged image stacks, and the original individual imaging datasets are not publicly available. Therefore, biological variability could not be estimated, and error bars are shown only for analyses based on original datasets (e.g., glutamate and GABA)."
